# The role of biocatalysis in the asymmetric synthesis of alkaloids – an update

**DOI:** 10.1039/d1ra04181a

**Published:** 2021-08-20

**Authors:** Emmanuel Cigan, Bettina Eggbauer, Joerg H. Schrittwieser, Wolfgang Kroutil

**Affiliations:** Institute of Chemistry, University of Graz, NAWI Graz, BioTechMed Graz, BioHealth Heinrichstrasse 28/II 8010 Graz Austria joerg.schrittwieser@uni-graz.at

## Abstract

Alkaloids are a group of natural products with interesting pharmacological properties and a long history of medicinal application. Their complex molecular structures have fascinated chemists for decades, and their total synthesis still poses a considerable challenge. In a previous review, we have illustrated how biocatalysis can make valuable contributions to the asymmetric synthesis of alkaloids. The chemo-enzymatic strategies discussed therein have been further explored and improved in recent years, and advances in amine biocatalysis have vastly expanded the opportunities for incorporating enzymes into synthetic routes towards these important natural products. The present review summarises modern developments in chemo-enzymatic alkaloid synthesis since 2013, in which the biocatalytic transformations continue to take an increasingly ‘central’ role.

## Abstract 2

This review article discusses developments in the chemo-enzymatic synthesis of alkaloids since 2013, showcasing how modern methods of organic synthesis and biocatalysis are combined to establish novel routes towards these important natural products.

## Graphical abstract

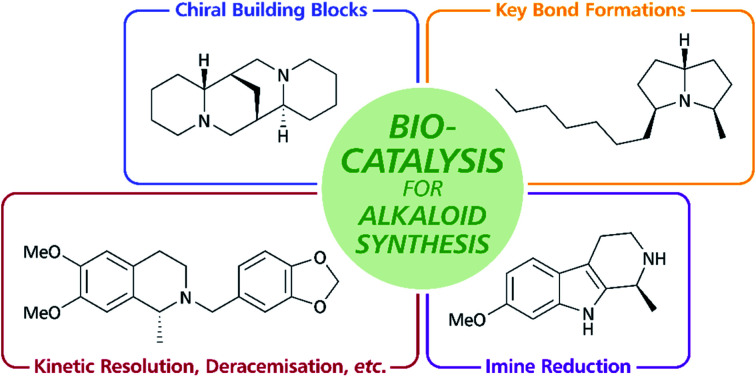

## 1 Introduction

The alkaloids are a large and structurally diverse group of nitrogen-containing secondary metabolites that are produced by a variety of organisms and that often possess potent biological activities.^1^ Although no universally accepted definition of the term ‘alkaloid’ has yet emerged,^2^ modern conceptions tend to be broad and include not only nitrogen compounds of basic character – the property from which the name was originally derived – but also amides, nitro, and nitroso compounds, while excluding primary metabolites such as amino acids, proteins, and porphyrins.^1*a*,*d*^ Between 25 000 and more than 40 000 known structures can be classified as alkaloids according to this broad definition, and examples of these natural products are found in almost all kingdoms of life.^1*a*–*d*^ The largest number of representatives, including those that are most widely known, originate from higher plants.

Humanity's interest in alkaloids is spurred mainly by the strong pharmacological effects that many of these compounds exhibit, and their use as medicinal and recreational drugs predates their identification by several thousand years.^1*c*,*f*,*g*^ Although most modern pharmaceuticals are synthetic, alkaloids continue to play an important role in present-day healthcare, and some have also found application in other fields, for instance, as flavour compounds or as chiral reagents in synthetic chemistry.^1*a*–*c*,1*e*–*g*^ Consequently, many alkaloids have significant commercial value (for examples, see Table 1) and are produced at volumes of several hundred to several thousand tonnes per year.

**Table 1:** Examples of commercially important alkaloids

Alkaloid	Applications	Major production methods	Annual production volume
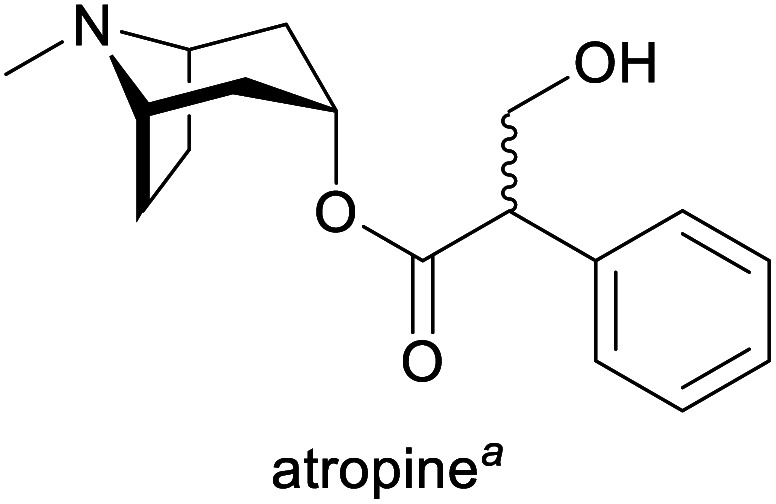	Treatment of organophosphate poisoning and bradycardia, as mydriatic and cycloplegic in ophthalmology, as preoperative medication (decrease of saliva production)^1*g*,3^	Isolation from *Atropa belladonna*, *Duboisia* and *Hyoscyamus* species^1*g*^	3.9 tonnes (2017)^4^
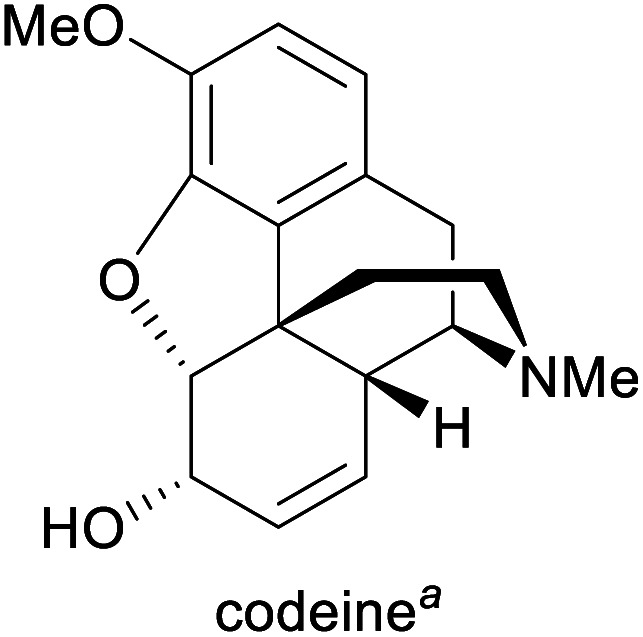	Pain treatment, cough suppression^1*g*,3^	Isolation from opium poppy, semisynthesis from morphine^1*g*^	308 tonnes (2018)^5^
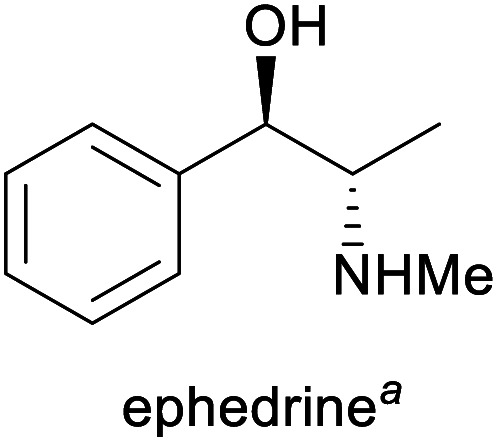	Prevention of hypotension during spinal anaesthesia,^3^ chiral auxiliary in asymmetric synthesis^6^	(Chemo-enzymatic) total synthesis, isolation from *Ephedra* plants^7^	1500–2000 tonnes^8^
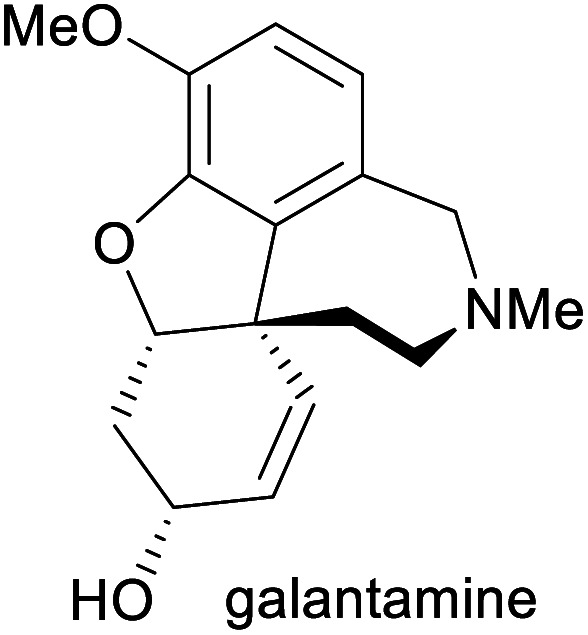	Treatment of Alzheimer's disease^9^	Total synthesis, isolation from daffodil bulbs^9,10^	3–4 tonnes (2014)^11^
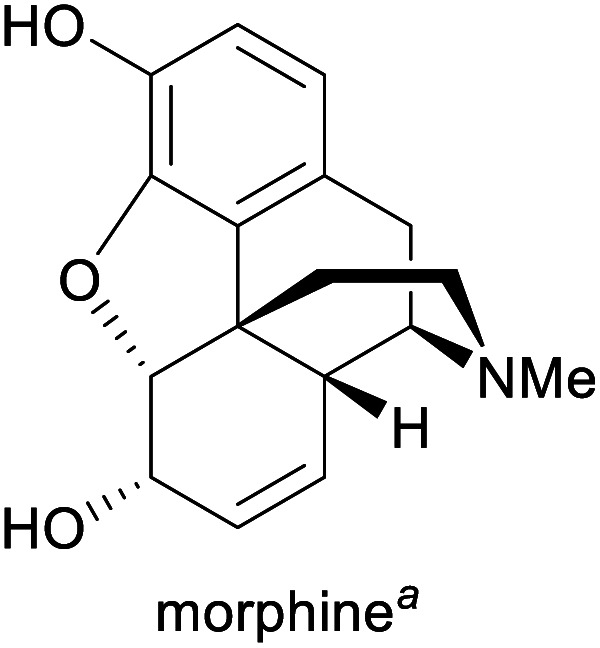	Pain treatment^1*g*,3^	Isolation from opium poppy^1*g*^	388 tonnes (2018)^5^
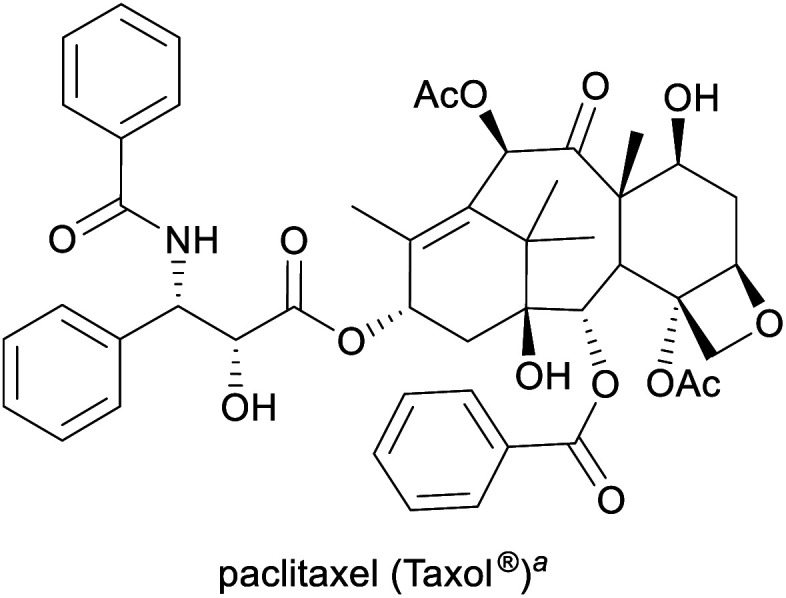	Cancer treatment^3,12^	Semisynthesis (from 10-deacetylbaccatin-III), plant cell fermentation^13^	2.6 tonnes (2017)^14^
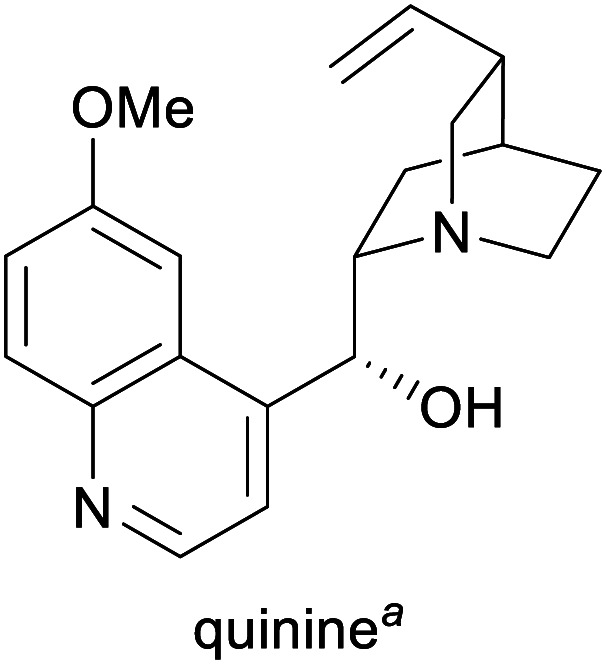	Treatment of malaria and babesiosis,^3,15^ as flavour compound,^1*g*^ chiral catalyst in asymmetric synthesis^16^	Isolation from cinchona bark^17^	300–500 tonnes^17^
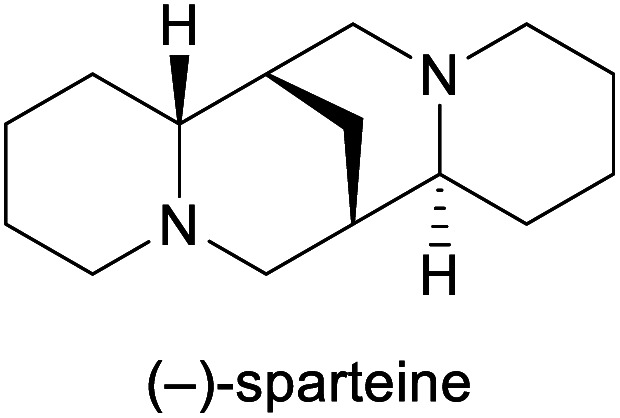	Chiral ligand in asymmetric synthesis^18^	Isolation from *Cytisus scoparius*^19^	

a Part of the World Health Organization's *Model List of Essential Medicines* (21st edn, 2019).^3^

Among the employed production methods, isolation from a natural source is still the most common, but examples of alkaloids exist that are prepared on industrial scale by total synthesis, semi-synthesis or plant cell fermentation. Each of these approaches has its benefits and limitations: Isolation and semi-synthesis, for instance, predominantly rely on plants as a raw material and are hence bound to seasonal availability of crops, climate and weather conditions, and natural variations in alkaloid content. Moreover, isolation yields are often low, and the commercial exploitation of endangered species can severely interfere with conservational efforts, as has been well documented for the cases of galantamine and paclitaxel.^10,13^ These factors, combined with cultivation of source plants in politically unstable regions of the earth, can lead to unpredictable supply situations. One recent example that has disconcerted the chemistry community is the one of (−)-sparteine, a quinolizidine alkaloid used as chiral ligand, which became entirely unavailable for some time in the early 2010s and remains scarce for reasons that are still unclear.^19^ Total synthesis, on the other hand, typically starts from petrol-based raw materials, which at present are still abundantly available. However, the intricate molecular architectures of many alkaloids present considerable synthetic challenges and often require lengthy routes to assemble, resulting in low overall yields and significant generation of waste. Besides, modern synthetic methods frequently employ catalysts based on precious metals that are in high demand also by other industries and are subject to substantial price fluctuations.^20^ Because of these limitations, only few total syntheses of alkaloids have been successfully translated to production scale.

Methods of biotechnology could help to establish novel production routes for alkaloids and thereby alleviate existing supply limitations.^21^ Indeed, some biotechnological approaches have already had a major impact: Random mutagenesis of opium poppy has been used to create a variant with altered alkaloid profile, which is now cultivated on large scale for the production of thebaine, a precursor to prescription opioids.^22^ Plant cell fermentation^23^ has enabled the commercial manufacture of paclitaxel by a process whose environmental advantages over semi-synthetic routes have been recognised with the US Environmental Protection Agency's 2004 *Greener Synthetic Pathways Award*.^13,24^ Other biotechnological methods of alkaloid production have not yet been applied on industrial scale, but have delivered promising results in laboratory experiments: Metabolic pathway engineering, for instance, makes use of the biosynthetic machinery that nature has evolved for the construction of secondary metabolites, but transposes it into production hosts that are more easily cultivated and optimised than the natural source organism.^25,26^ Recently, this approach has been used to create transgenic yeast strains that assemble opioids,^27^ tetrahydroisoquinolines,^28^ or tropanes^29^ from simple sugar and amino acid building blocks, in some cases with impressive product titres [*e.g.*, 4.6 g L^−1^ for (*S*)-reticuline].

Biocatalysis, *i.e.*, the use of enzymes or microbial cells in preparative transformations,^30^ has been established as a valuable synthetic tool in the pharmaceutical industry,^31^ and its potential for the synthesis of complex natural products, including alkaloids, has also been recognised.^32^ Biocatalytic reactions typically offer high chemo-, regio-, and stereoselectivity, but also a substrate scope that is usually broad enough to allow not only the preparation of one particular product but a series of structurally related derivatives. Integrating *in vitro* biotransformations into the total synthesis or semi-synthesis of alkaloids can hence lead to shorter routes and a reduced need for protective groups, while avoiding limitations of productivity and flexibility that are often associated with fermentation approaches.

In a previous review, published in 2013, we have identified three main strategies for the integration of biocatalysis into alkaloid synthesis: (1) the biocatalytic preparation of chiral building blocks that are chemically transformed into the target compounds, (2) the biocatalytic kinetic resolution, desymmetrisation, or deracemisation of alkaloids that have been synthesised by chemical methods, and (3) the construction of alkaloids using biocatalytic C–N and/or C–C bond formation in the asymmetric key step.^33^ In the roughly eight years that have passed since then, exciting new developments have been made in all three of these areas, and the overarching trend towards a more central role of biocatalysis in chemo-enzymatic alkaloid syntheses, which we have identified in our earlier review, has continued. Moreover, the identification of imine-reducing enzymes with broad substrate scope and high stereoselectivity has enabled biocatalytic C=N reduction as a fourth approach to the chemo-enzymatic asymmetric synthesis of alkaloids. In this update to our previous review, we therefore intend to show how the discovery of novel enzymes, the engineering of biocatalysts, but also the creative application of well-known biotransformations have led to many new, elegant routes towards a broad range of alkaloids, and thereby have continued to re-shape the role of biocatalysis in the asymmetric synthesis of these fascinating natural products.

## 2 Biocatalytic asymmetric synthesis of chiral building blocks

The use of enantiomerically pure building blocks for constructing complex target molecules with substrate-based stereocontrol is a classical approach to asymmetric total synthesis. The traditional source for these building blocks – the ‘chiral pool’ of naturally occurring amino acids, sugars, and terpenes – has recently enjoyed a renewed interest from organic chemists as they strive to incorporate renewable feedstocks into their synthesis routes.^34^ Alternatively, biocatalytic transformations can be used to prepare a wide range of chiral building blocks, which can often be tailored more closely to the desired target structure than the compounds available in nature.^35^ This strategy is frequently employed in alkaloid synthesis, and lipases and esterases as well as toluene dioxygenase remain the most important enzymes in this context.

### 2.1 Lipases and esterases

The enantioselective hydrolysis and formation of ester bonds catalysed by lipases and esterases has been an early focus of biocatalysis research and has found broad application in asymmetric synthesis.^36^ As discussed in our previous review,^33^ chiral building blocks prepared using lipases and esterases have also been employed frequently in chemo-enzymatic syntheses of alkaloids. While early examples have focused on a few selected classes of building blocks, in particular cyclic alcohols and piperidine derivatives, recent contributions have made use of a much more diverse range of structures. Moreover, besides kinetic resolution and desymmetrisation, dynamic kinetic resolution has recently been explored as an additional option for the lipase-catalysed synthesis of chiral building blocks in alkaloid chemistry.

#### 2.1.1 Kinetic resolution of alcohols

**Scheme 1:**
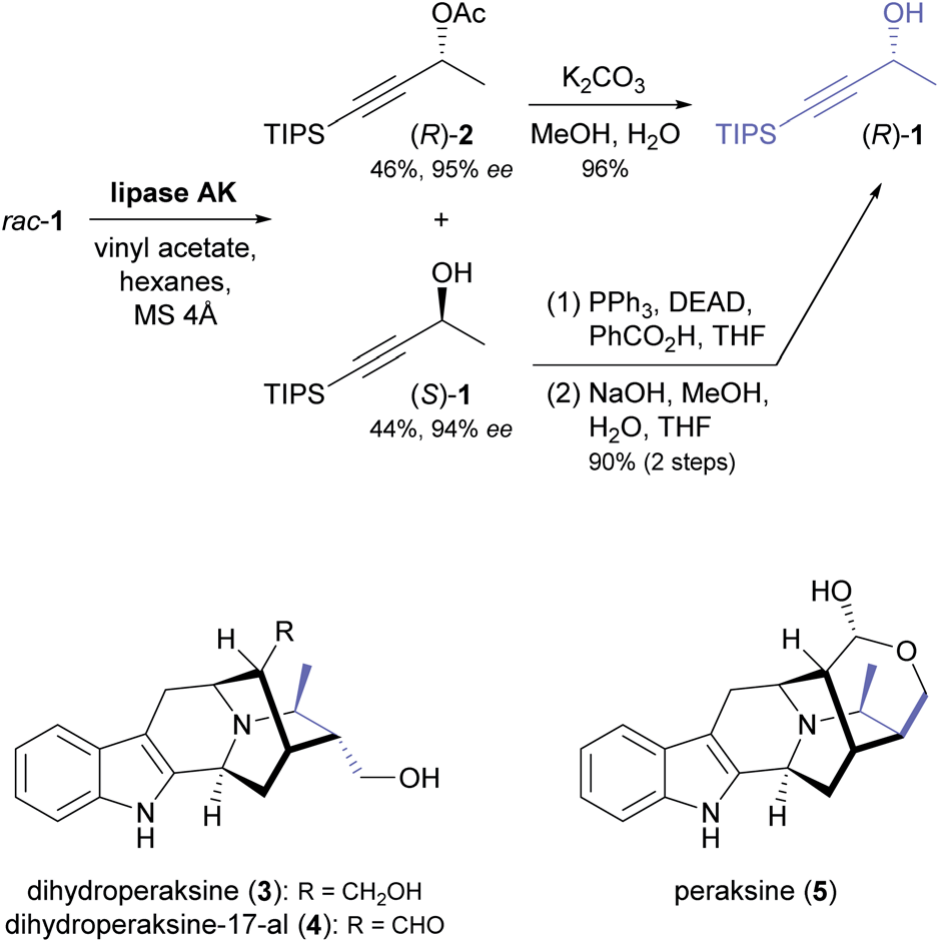
Synthesis of building block (*R*)-1 by lipase-catalysed kinetic resolution, and structures of indole alkaloids prepared from this building block.^39^

Despite its intrinsic limitation to 50% yield for either enantiomer, kinetic resolution is still the most widely used approach in the lipase- or esterase-catalysed synthesis of chiral building blocks. The kinetic resolution of secondary alcohols by enzymatic *O*-acylation is particularly common, because it usually proceeds with reliably high enantioselectivity. In an impressive recent example, the TIPS-protected propargylic alcohol 1 (Scheme 1) was resolved using lipase AK^37^ on a 104 gram scale, affording (*S*)-1 in 44% yield and 94% *ee* along with the (*R*)-acetate 2 in 46% yield and 95% *ee* (*E* = 139).^38^ As only the (*R*)-enantiomer was required for the downstream synthetic sequence, the authors implemented a stereoconvergent strategy to maximise its yield: Saponification of (*R*)-2 gave (*R*)-1 directly, while (*S*)-1 was subjected to Mitsunobu inversion *via* benzoylation followed by hydrolysis, which also led to (*R*)-1 in 90% yield and with no erosion of optical purity. The (*R*)-alcohol was then converted into the indole alkaloids dihydroperaksine (3), dihydroperaksine-17-al (4), and peraksine (5) in 11–13 additional steps, which include coupling with a d-tryptophan-derived tetracyclic lactam, iodoboration of the alkyne group, and a palladium(0)-catalysed intramolecular α-vinylation of a ketone moiety.

**Scheme 2:**
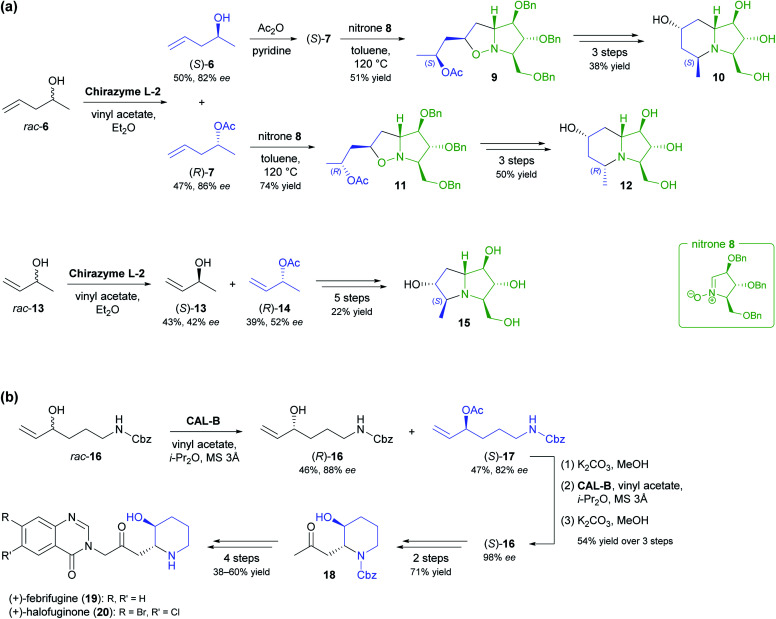
Olefinic secondary alcohols as building blocks in alkaloid synthesis: (a) Kinetic resolution of alcohols 6 and 13 and their conversion into polyhydroxylated pyrrolizidines and an indolizidine. (b) Chemo-enzymatic synthesis of (+)-febrifugine (19) and (+)-halofuginone (20) based on the kinetic resolution of alcohol 16.

The lipase-catalysed kinetic resolution of two small, olefinic *sec*-alcohols, 6 and 13 (Scheme 2a), was employed in the preparation of polyhydroxylated pyrrolizidines and an indolizidine that are close structural analogues of hyacinthamines and steviamine, respectively.^40^ Acylation of the alcohols using Chirazyme L-2 (ref. 37) and vinyl acetate in diethyl ether at room temperature proceeded with only moderate enantioselectivity (6: *E* = 34, 13: *E* = 5), but the resulting limitations in optical purity of the reaction products could be tolerated, since their subsequent coupling with the enantiomerically pure nitrone 8 produced separable mixtures of diastereomers. Limited enantioselectivity was also observed in the kinetic resolution of alcohol 16 by lipase B from *Candida antarctica* (Scheme 2b, *E* = 29), carried out as part of a chemo-enzymatic synthesis of both enantiomers of the quinazoline alkaloid febrifugine (19) and its non-natural derivative halofuginone (20).^41^ The (*R*)-enantiomer of 16 was isolated from the lipase biotransformation in 46% yield and 88% *ee* and used as such in further steps. The corresponding (*S*)-acetate (17), obtained in 47% yield and 82% *ee*, was hydrolysed back to the alcohol and its optical purity upgraded to 98% *ee* by a second round of kinetic resolution. Alkene cross-metathesis, a diastereoselective *aza*-Michael addition, and α-functionalisation of methyl ketone 18 completed the syntheses of febrifugine and halofuginone.

**Scheme 3:**
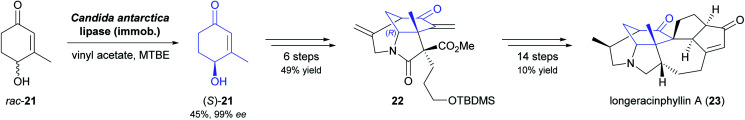
Chemo-enzymatic asymmetric synthesis of longeracinphyllin A (23).

A recent synthesis of the hexacyclic *Daphniphyllum* alkaloid longeracinphyllin A (also known as daphniynnine C;^42^23, Scheme 3) uses hydroxyketone (*S*)-21, prepared in 45% yield and 99% *ee* by a 74 gram-scale kinetic resolution of the racemate with immobilised CAL-B, as starting point and precursor to the central cyclohexanone ring of the target structure.^43^ From there, the complex alkaloid scaffold was constructed with remarkable efficiency, arriving at a tricyclic intermediate (22) in only three operations (six steps) that include a Toste-type silyl enol ether cyclisation^44^ and a Michael addition/aldol condensation cascade. The synthesis was completed in 14 more steps, with the remaining three ring closures being achieved by intramolecular conjugate addition, a (3 + 2) enone–allene cycloaddition, and a Horner–Wadsworth–Emmons reaction.

**Scheme 4:**
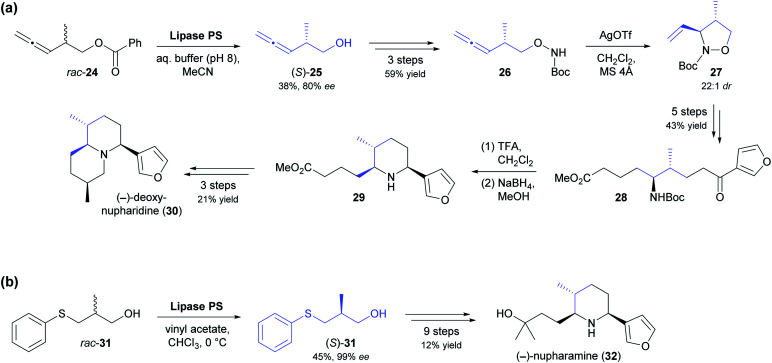
Lipase-catalysed kinetic resolution of β-chiral primary alcohols in the asymmetric synthesis of Nuphar alkaloids: (a) chemo-enzymatic synthesis of (−)-deoxynupharidine (30), (b) chemo-enzymatic synthesis of (−)-nupharamine (32).

The kinetic resolution of racemic primary alcohols is more challenging than that of *sec*-alcohols because the stereogenic centre of the substrate is further away from the reacting hydroxyl group, which often results in lower enantioselectivity. Nevertheless, the lipase-catalysed kinetic resolution of β-chiral primary alcohols was successfully used in two recent syntheses of Nuphar alkaloids. In the first of these studies, the allenic benzoate ester 24 (Scheme 4a) was enantioselectively hydrolysed by lipase PS^37^ to obtain alcohol (*S*)-25 in 38% yield and 80% *ee* (*E* = 15).^45^ Transformation of the hydroxyl group into a Boc-protected hydroxylamine set the stage for a silver(i)-catalysed cyclisation that gave isoxazolidine 27 with excellent diastereoselectivity (22 : 1 *dr*). From this intermediate, (−)-deoxynupharidine (30) was prepared in nine steps, whereby the third chirality centre was formed with complete diastereocontrol in an intramolecular reductive amination reaction. In unrelated work, (−)-nupharamine (32), a piperidine Nuphar alkaloid, was synthesised from alcohol (*S*)-31, which was prepared by acylative kinetic resolution of the racemate, again using lipase PS (Scheme 4b).^46^ The enantioselectivity of the biotransformation was excellent in this case (*E* ≥50), resulting in 45% yield and 99% *ee* of (*S*)-31, while the corresponding (*R*)-acetate was not isolated. The optically pure alcohol was elaborated into the target alkaloid in nine steps with an overall yield of 12%.

**Scheme 5:**
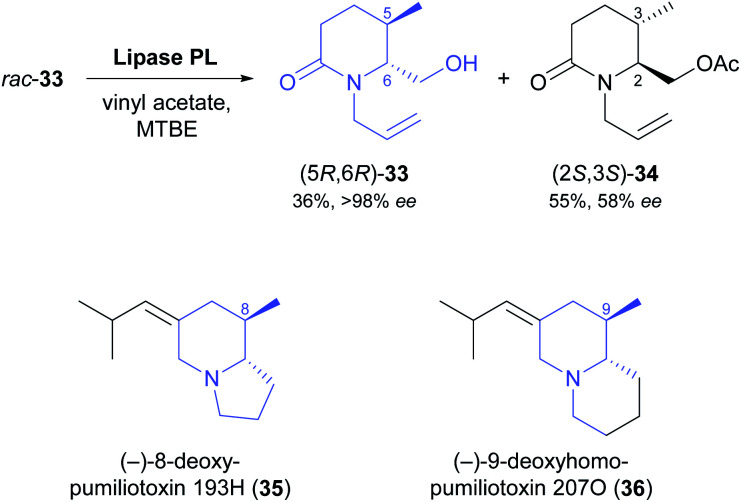
Lipase-catalysed kinetic resolution of building block 33, and structures of alkaloids prepared from this building block.

Two recent studies demonstrate that fairly complex chiral building blocks can be prepared by the kinetic resolution of primary alcohols. Attaining a sufficient enantioselectivity proved difficult also in these examples, making two consecutive rounds of kinetic resolution necessary to obtain both enantiomers in satisfactory optical purity. As part of their recent synthesis of two pumiliotoxins, Okada, Toyooka, and co-workers carried out the kinetic resolution of alcohol 33 using lipase PL^37^ and vinyl acetate in MTBE at room temperature (Scheme 5).^47^ While the unreacted alcohol (5*R*,6*R*)-33 was recovered in 36% yield and >98% *ee*, the corresponding acetate (2*S*,3*S*)-34 was isolated in 55% yield but only 58% *ee*, indicating moderate enantioselectivity (*E* = 16). After ester hydrolysis, enantioenriched (5*S*,6*S*)-33 was subjected to a second round of kinetic resolution under identical conditions, which raised the enantiomeric excess of (2*S*,3*S*)-34 to 98% (at 47% yield). The optically pure building blocks thus obtained were converted into both enantiomers of 8-deoxypumiliotoxin 193H (35) and 9-deoxyhomopumiliotoxin 207O (36) in seven and eight chemical steps, respectively.

**Scheme 6:**
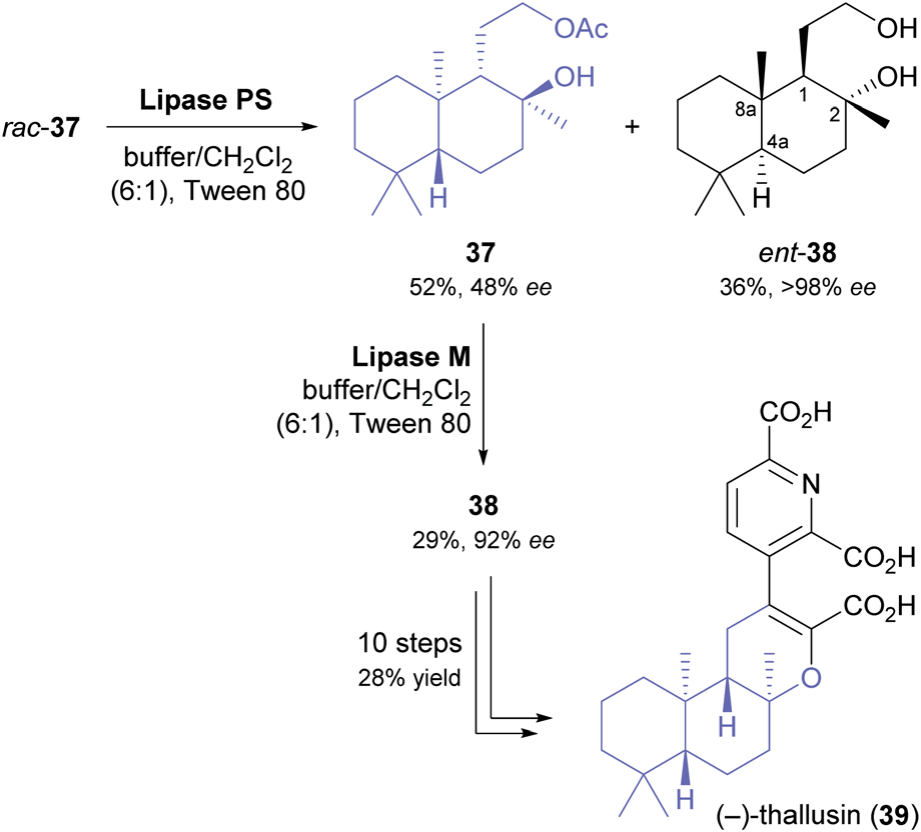
Preparation of building block 38 by double kinetic resolution, and its use in the chemo-enzymatic asymmetric synthesis of (−)-thallusin (39).

Yamamoto and co-workers, in their synthesis of (−)-thallusin (39), took a slightly more elaborate approach for upgrading the optical purity of the desired enantiomer of building block 38 (Scheme 6).^48^ After hydrolytic kinetic resolution of acetate *rac*-37 under literature-known conditions (lipase PS,^37^ phosphate buffer/dichloromethane = 6 : 1, Tween 80, 30 °C)^49^ had provided the unreacted ester in 52% yield and 48% *ee*, along with the corresponding alcohol in 36% yield and >98% *ee* (*E* = 160), the authors screened five alternative lipases for a second round of kinetic resolution of enantioenriched 37. Lipase M turned out to preferentially hydrolyse the major enantiomer (*i.e.*, it showed opposite enantioselectivity to lipase PS), giving (1*S*,2*S*,4a*R*,8a*R*)-38 in 92% *ee* and 29% yield. With all stereogenic centres of the target molecule thus in place, the synthesis of (−)-thallusin was completed in a 10-step sequence.

#### 2.1.2 Dynamic kinetic resolution of alcohols

**Scheme 7:**
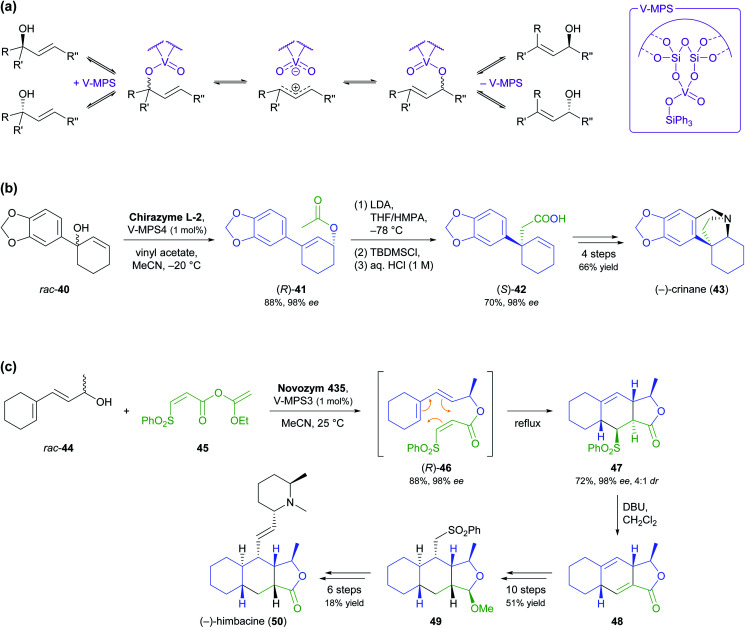
Dynamic kinetic resolution (DKR) of allylic alcohols in the asymmetric synthesis of alkaloids: (a) general racemisation mechanism of oxovanadium catalyst immobilised on mesoporous silica (V-MPS), (b) DKR of 40 followed by Ireland–Claisen rearrangement in the synthesis of (−)-crinane (43), (c) DKR of 44 followed by Diels–Alder cycloaddition in the synthesis of (−)-himbacine (50).

In 2013, Shuji Akai and co-workers have reported an oxovanadium catalyst immobilised on mesoporous silica that effects the racemisation of allylic alcohols *via* 1,3-transposition of the hydroxyl group (Scheme 7a) and can be combined with lipases to achieve dynamic kinetic resolution (DKR).^50^ More recently, the same research group has applied this methodology to the asymmetric synthesis of the alkaloids (−)-crinane (43) and (−)-himbacine (50, non-natural enantiomer). In the first case, the DKR was performed on the tertiary alcohol 40, resulting in its transformation into the allylic acetate (*R*)-41 with 88% yield and 98% *ee* (Scheme 7b).^51^ An Ireland–Claisen rearrangement was then used for a 1,3-transfer of chirality that set up the quaternary stereocentre of the target compound and proceeded with full conservation of enantiomeric purity. The total synthesis of (−)-crinane was completed in four more steps with an overall yield of 66% (41% from 40). In the second case, DKR using Novozym 435 and the oxovanadium catalyst transformed the open-chain allylic alcohol 44 into the ester (*R*)-46, which upon heating to reflux underwent an intramolecular Diels–Alder cycloaddition that afforded the lactone 47 in 98% *ee* and as a 4 : 1 mixture of diastereomers (Scheme 7c).^52^ Treatment with DBU in dichloromethane at room temperature quantitatively converted this isomeric mixture into diene 48, and this intermediate was further elaborated into (−)-himbacine in 16 steps.

#### 2.1.3 Desymmetrisation of alcohols and anhydrides

**Scheme 8:**
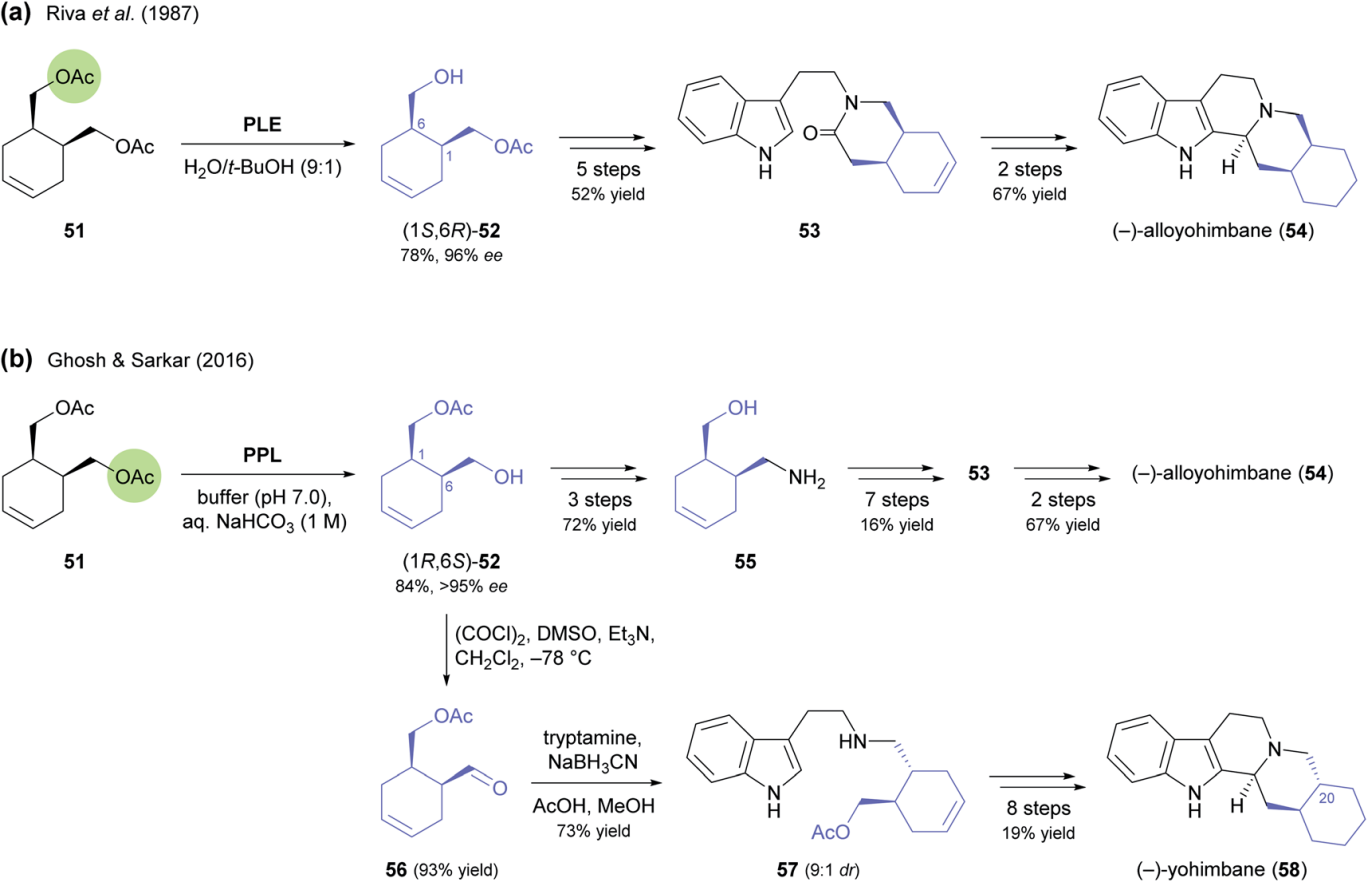
Preparation of building block 52 by hydrolase-catalysed desymmetrisation and its use in the synthesis of yohimbe alkaloids: (a) synthesis of (−)-alloyohimbane (54) from (1*S*,6*R*)-52, (b) synthesis of (−)-alloyohimbane (54) and (−)-yohimbane (58) from (1*R*,6*S*)-52.

In contrast to kinetic resolution, lipase-catalysed desymmetrisation offers the advantage of providing access to a single enantiomer in 100% maximum theoretical yield. In turn, it requires a prochiral or *meso*-compound as substrate, while a kinetic resolution can in principle be performed on any racemic molecule.^53^ Consequently, hydrolase-catalysed desymmetrisation reactions are generally less common than kinetic resolutions. Nevertheless, the earliest example of the use of a chiral building block in alkaloid synthesis – published in 1987 by Renata Riva and co-workers,^54^ and discussed in our previous review – features the desymmetrisation of *meso*-diacetate 51 (Scheme 8) as an asymmetric key step in the preparation of the yohimbe bark constituent (−)-alloyohimbane (54). Interestingly, a very similar approach was recently used by Ghoch & Sarkar in their synthesis of the same alkaloid and its C20 epimer, (−)-yohimbane (58).^55^ While in the 1987 report the selective monohydrolysis of 51 was achieved by pig liver esterase (PLE), resulting in formation of monoacetate (1*S*,6*R*)-52 (Scheme 8a), the authors of the recent study relied on porcine pancreatic lipase, which afforded the opposite enantiomer, (1*R*,6*S*)-52, in 84% yield and >95% *ee* (Scheme 8b). They then elaborated this chiral building block into the amino alcohol 55*via* the corresponding azide and converted 55 into the lactam 53, a compound that was also a late intermediate in Riva's synthetic sequence. Bischler–Napieralski cyclisation followed by NaBH_4_ reduction and hydrogenation of the olefinic double bond completed the synthesis of (−)-alloyohimbane (54). For the synthesis of its epimer, Ghosh & Sarkar oxidised (1*R*,6*S*)-52 to aldehyde 56, which upon reductive amination with tryptamine afforded the *trans*-configured coupling product 57 in 9 : 1 excess over the *cis*-isomer, most likely *via* epimerisation by imine–enamine tautomerism. Conversion of 57 into (−)-yohimbane (58) was achieved by the same sequence of steps used for 54.

**Scheme 9:**
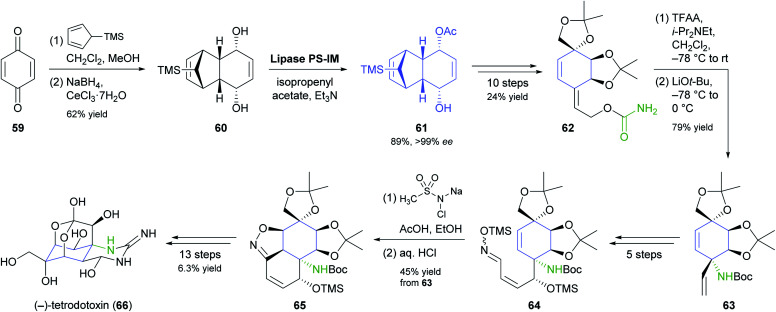
Fukuyama's chemo-enzymatic total synthesis of (−)-tetrodotoxin (66).

Desymmetrisation of the tricyclic *meso*-compound 60 (Scheme 9) is the asymmetric key step in Fukuyama's recent synthesis of the neurotoxic puffer fish alkaloid (−)-tetrodotoxin.^56^ The diol 60, prepared by a Diels–Alder reaction of *para*-benzoquinone (59) with 5-TMS-cyclopentadiene followed by Luche reduction, was stereoselectively acetylated by lipase PS-IM^37^ under previously reported conditions^57^ to give monoacetate 61 in 89% yield and >99% *ee*. From there, the target compound was assembled in a 29-step sequence that exploits facial selectivity in the tricyclic intermediate 61 for controlling three stereogenic centres of the central cyclohexane ring, while the remaining three were installed by a [3,3]-sigmatropic rearrangement of an allylic cyanate and an intramolecular 1,3-dipolar cycloaddition of a nitrile oxide.

**Scheme 10:**
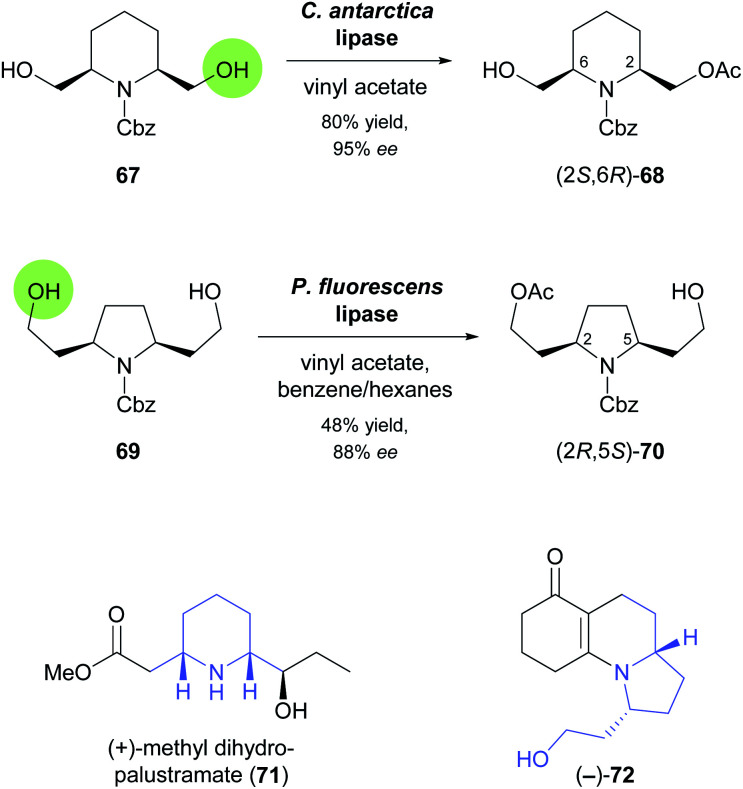
Biocatalytic preparation of piperidine building block 68 and pyrrolidine building block 70, and structures of target molecules prepared from these building blocks.

In the 1990s, disubstituted piperidine derivatives were used extensively as substrates for lipase-catalysed desymmetrisation and as building blocks for alkaloid synthesis.^58^ One of the reactions studied during this time is the enantiotoposelective acetylation of *meso*-diol 67 by *Candida antarctica* lipase, which affords the monoacetate (2*S*,6*R*)-68 in 80% yield and 95% *ee* (Scheme 10).^59^ In a more recent study, this building block was employed in the synthesis of (+)-methyl dihydropalustramate (71), a degradation product of the alkaloid (+)-palustrine.^60^ Key transformations of the 8-step synthetic sequence are a Wittig reaction, a Seyferth–Gilbert homologation, and the ruthenium(ii)-catalysed *anti*-Markovnikov hydration of a terminal alkyne. The acetylative desymmetrisation of a structurally related diol, 69 (Scheme 10), employing a commercial lipase from *Pseudomonas fluorescens*, was used to access the tricyclic enaminone (−)-72, a known intermediate^61^ in the synthesis of the poison frog alkaloid (−)-gephyrotoxin.^62^ The biotransformation furnished the (2*R*,5*S*)-enantiomer of monoacetate 70 in 48% yield and 88% *ee*, indicating that compared to 67 the increased distance of the reactive atom from the *meso* “core” of the molecule did not only lead to lower selectivity but also to a switch in the recognised enantiotopic group. From (2*R*,5*S*)-70, the target enaminone was obtained in a straightforward sequence comprised of Swern oxidation of the free alcohol, a proline-catalysed, reductive Knoevenagel condensation, and two deprotection steps.

**Scheme 11:**
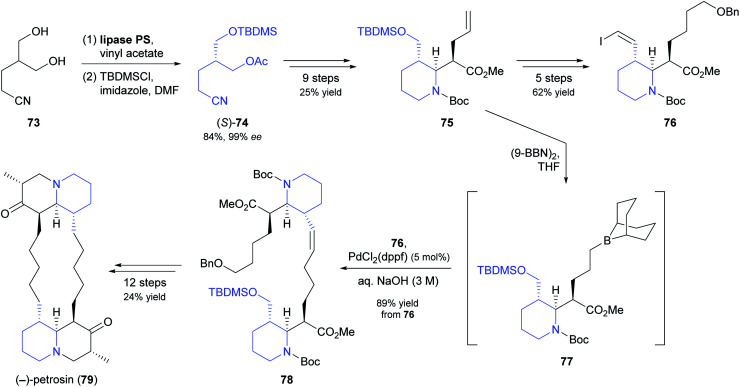
Lipase-catalysed desymmetrisation of 1,3-diol 73 in the total synthesis of the bisquinolizidine alkaloid (−)-petrosin (79).

Open-chain *meso*-1,3-diols are challenging substrates for desymmetrisation because racemisation of the resulting monoacetates by acyl group migration can occur, leading to reduced enantiomeric purity.^63^ In their chemo-enzymatic synthesis of the marine sponge alkaloid (−)-petrosin (79, Scheme 11), Hidetoshi Tokuyama and co-workers managed to prevent this side reaction by immediate silyl-protection of the free hydroxyl group.^64^ Hence, they reacted diol 73 with vinyl acetate under lipase PS^37^ catalysis, and – after filtration and evaporation of excess acyl donor – treated the crude monoacetate with TBDMS chloride to give compound (*S*)-74 in 84% yield (two steps) and 99% *ee*. This building block was elaborated into the piperidine derivative 75, which in turn served as precursor to vinylic iodide 76 and borane 77. Suzuki–Miyaura coupling of the two latter compounds gave olefin 78, from which the bisquinolizidine alkaloid 79 was prepared in 12 additional steps.

**Scheme 12:**
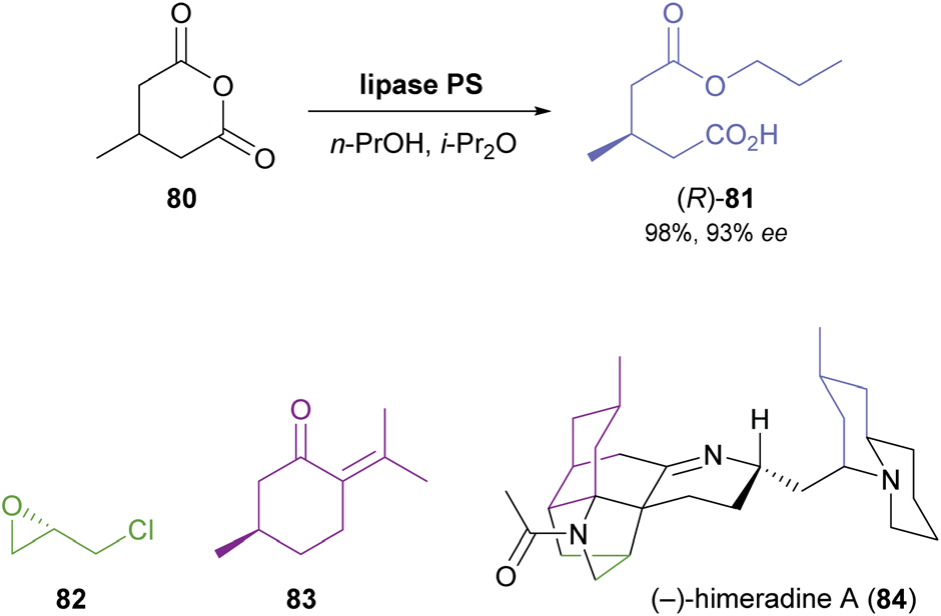
Lipase-catalysed desymmetrisation of cyclic anhydride 80, and the structures of (*S*)-epichlorohydrin (82), (*R*)-pulegone (83), and (−)-himeradine A (84).

The lipase-catalysed desymmetrisation of cyclic anhydrides is much less common than the desymmetrisation of diols or diesters, perhaps due to the intrinsically high reactivity of anhydrides that can give rise to undesired non-enzymatic background reactivity.^65^ Nevertheless, the alcoholysis of 3-methylglutaric anhydride (80, Scheme 12) by lipase PS^37^ is known to provide monoester 81 in excellent yield (95–98%) and high optical purity (92–93%).^66^ Matthew Shair and co-workers, of Harvard University, recently used this biotransformation in constructing the quinolizidine subunit of the proposed structure of (−)-himeradine A (84), a *Lycopodium* alkaloid.^66*b*^ The pentacyclic core of the target molecule, on the other hand, was assembled from ‘chiral pool’ building blocks (*S*)-epichlorohydrin (82) and (*R*)-pulegone (83) in a multi-step sequence that the authors adapted for the synthesis of other *Lycopodium* alkaloids sharing the same scaffold.

#### 2.1.4 Kinetic resolution of esters and lactones

**Scheme 13:**
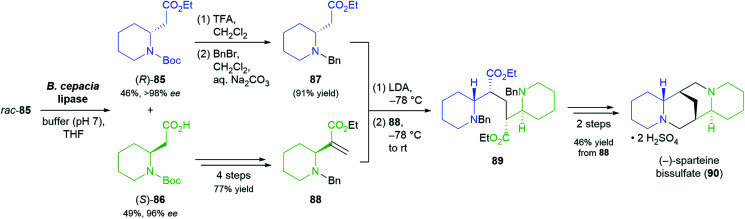
Lipase-catalysed kinetic resolution of building block 85 in the asymmetric synthesis of (−)-sparteine bissulfate (90).

Lipases are frequently employed for the hydrolytic kinetic resolution of esters, whereby the stereogenic centre is usually located in the alcohol part of the molecule. Examples of the opposite case, where the ester of a chiral carboxylic acid is resolved, are rare, but one recent study used this type of biotransformation to great effect in the asymmetric total synthesis of (−)-sparteine.^67^ O'Brien and co-workers recognised that the two lateral piperidinyl rings of the target alkaloid can be retrosynthetically traced back to the two enantiomers of ester 85 (Scheme 13), the kinetic resolution of which is a literature-known reaction.^68^ Slight modifications of the literature procedure enabled the enantioselective hydrolysis of 85 by *Burkholderia cepacia* lipase to be carried out at 10 g scale, yielding 49% of the (*S*)-acid 86 in 96% *ee* and 46% of (*R*)-85 in >98% *ee* (*E* >200). The latter was converted from the *N*-Boc to the *N*-benzyl derivative, which after deprotonation by LDA underwent conjugate addition to the α,β-unsaturated ester 88, obtained in four steps from (*S*)-86. *N*-Deprotection by catalytic hydrogenation triggered ring closure to a bislactam, which was converted into (−)-sparteine (isolated as the bissulfate, 90) by reduction with lithium aluminium hydride. In the same study, (−)-sparteine surrogate, a non-natural derivative developed by O'Brien's group,^69^ was prepared in 8 steps from (*R*)-85.

**Scheme 14:**
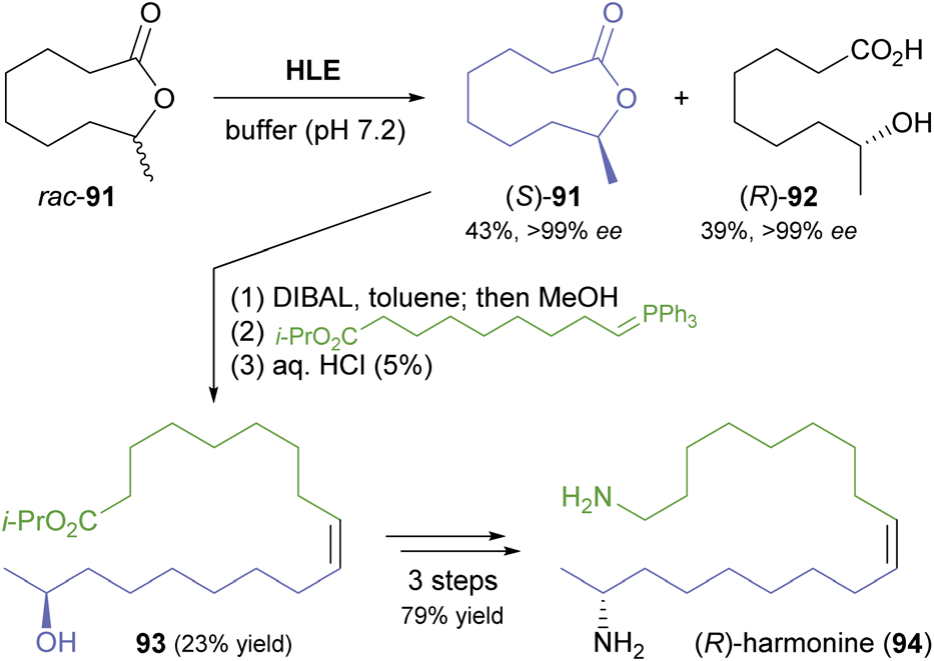
Hydrolytic kinetic resolution of lactone 91 by horse liver esterase (HLE) used in the chemo-enzymatic synthesis of (*R*)-harmonine (94).

Another rather unusual type of hydrolase reaction, the kinetic resolution of a lactone, was used in a recent synthesis of (*R*)-harmonine (94, Scheme 14), the toxic principle of the Asian lady beetle.^70^ The nine-membered chiral lactone 91 was hydrolysed by horse liver esterase with excellent selectivity for the (*R*)-enantiomer (*E* >200), affording the (*R*)-hydroxy acid 92 in 39% yield along with 43% of the unreacted (*S*)-lactone, both in optically pure form (*ee* >99%). The latter compound was reduced to the hydroxy aldehyde and directly subjected to a Wittig reaction to give a hydroxy ester (93) that already contained the complete carbon skeleton of the target compound. Reduction to the diol and conversion of both hydroxyl groups to amines *via* the bis-azide completed the synthesis of (*R*)-harmonine.

### 2.2 Toluene dioxygenase

**Scheme 15:**
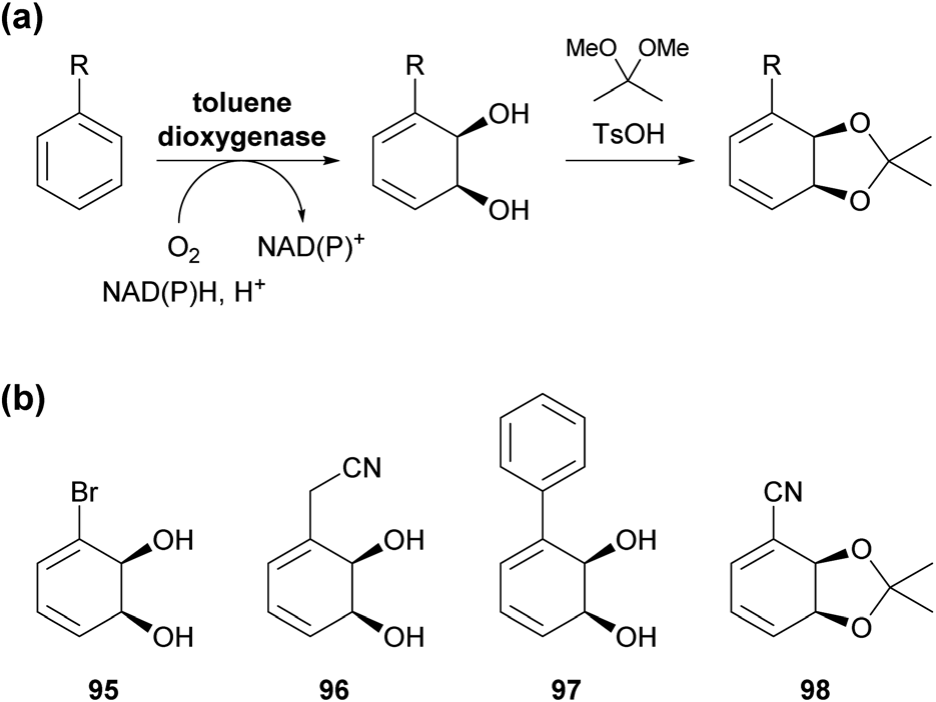
Arene *cis*-dihydroxylation by toluene dioxygenase (TDO): (a) general reaction scheme for the dihydroxylation of a monosubstituted arene by TDO and for the conversion of the resulting diol into an acetonide. (b) Examples of commercially available arene *cis*-dihydrodiols or the corresponding acetonides (as of January 2021).

Toluene dioxygenase (TDO) from *Pseudomonas putida*, an iron–sulfur protein first reported and characterised in the late 1960s,^71^ oxidises toluene and other mono- and disubstituted arenes to the corresponding *cis*-dihydrodiols, usually with excellent stereoselectivity (Scheme 15a). The versatile reactivity of these compounds has enabled their exploitation in the asymmetric synthesis of a broad range of complex target molecules, and consequently TDO has become the most widely used dioxygenase in organic synthesis.^72^ Due to the success of arene *cis*-dihydrodiols as chiral building blocks, several of these compounds are now commercially available (for examples, see Scheme 15b), making them accessible also to researchers that lack the necessary equipment or expertise for carrying out TDO biotransformations.

**Fig. 1:**
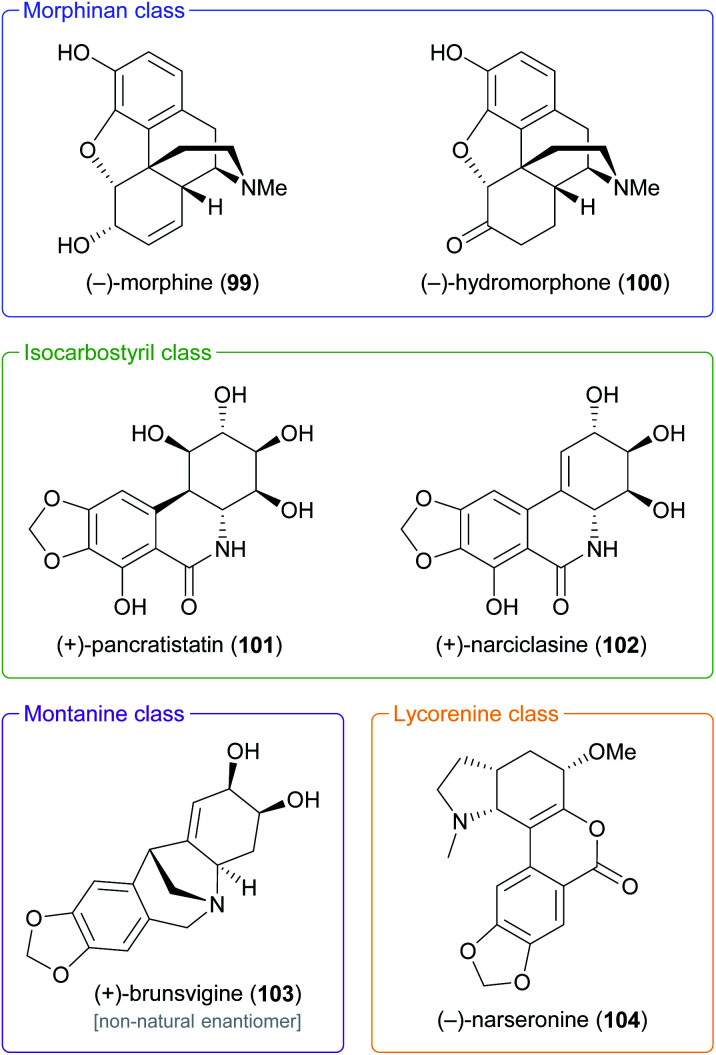
Examples of alkaloid classes targeted by TDO-based chemo-enzymatic syntheses.

**Scheme 16:**
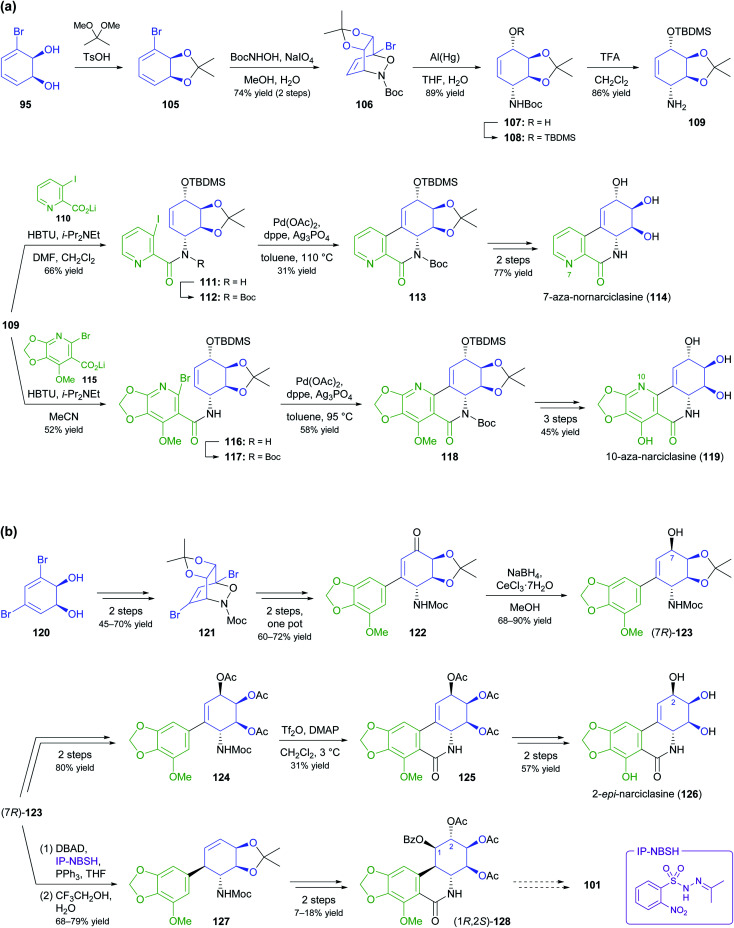
Recent application of TDO-derived chiral building blocks in the synthesis of isocarbostyril alkaloids: (a) transformation of building block 95 into 7-aza-nornarciclasine (114) and 10-aza-narciclasine (119), (b) transformation of building block 120 into 2-*epi*-narciclasine (126) and a late synthetic intermediate 128 of (+)-pancratistatin (101).

In the context of alkaloid synthesis, TDO-derived building blocks have been most intensively used by the groups of Tomas Hudlicky, who has developed several TDO-based chemo-enzymatic approaches to morphinan and isocarbostyril alkaloids over the course of the last 30 years, and of Martin Banwell, who has focused on *Amaryllidaceae* alkaloids of the montanine and lycorenine classes (Fig. 1).^73^ Both groups have continued these efforts in recent years, with a shift of focus to the preparation of non-natural alkaloid derivatives following synthetic strategies developed in earlier work. This trend is exemplified by the preparation of 7-aza-nornarciclasine (114) and 10-aza-narciclasine (119), two isocarbostyril analogues featuring a nitrogen atom in the A-ring (Scheme 16a).^74^ Both syntheses commenced with the transformation of TDO-derived *cis*-diol 95 into the conduramine derivative 109*via* an acyl-nitroso-diene [4 + 2] cycloaddition (nitroso-Diels–Alder reaction) followed by reduction with aluminium amalgam and protective-group manipulations. Compound 109, which contains the four contiguous stereogenic centres of the target alkaloids in the correct configuration, was then coupled with the lithium carboxylates 110 and 115 to give the amides 111 and 116, respectively. Closure of ring B by a Heck reaction proceeded in moderate yield in both cases (31% for 113 and 58% for 118), and deprotection furnished the target compounds. Biological evaluation showed the 7-aza-derivative 114 to be inactive against human cancer cell lines,^74*a*^ while 10-aza-narciclasine (119) did show significant cytotoxic activity.^74*b*^ A related synthetic approach was used to prepare 2-*epi*-narciclasine (126, Scheme 16b), a trace constituent of snowdrop (*Galanthus*) bulb extracts.^75^ In this work, TDO-catalysed oxidation of *meta*-dibromobenzene afforded the diol 120, which was protected as the usual acetonide and transformed into ketone 122*via* a nitroso-Diels–Alder reaction, Suzuki coupling, and Mo(CO)_6_ reduction. Luche reduction then established the desired (*R*)-configuration at C7 (123; C2 in isocarbostyril numbering). After protection of the three vicinal hydroxyl groups as acetates, a modified Bischler–Napieralski cyclisation was used to construct ring B, and deprotection gave the target alkaloid. The allylic alcohol 123 that is a central intermediate in this sequence also proved useful in a formal total synthesis of (+)-pancratistatin (101) that uses 1,3-chirality transfer by Myers' reductive transposition as a key step (Scheme 16b).^76^ While this transposition could be accomplished in satisfactory yield (68–79%), *trans*-dihydroxylation of the resulting olefin 127 by literature-known methods was unsuccessful. The authors, therefore, chose to establish the required (1*R*,2*S*)-stereochemistry (isocarbostyril numbering) through *cis*-dihydroxylation followed by preparation and ring-opening of a cyclic sulfate. Closure of ring B was again accomplished by a modified Bischler–Napieralski cyclisation, and although both of the latter two reactions produced regioisomeric mixtures, the desired isomers were favoured in both cases.

**Scheme 17:**
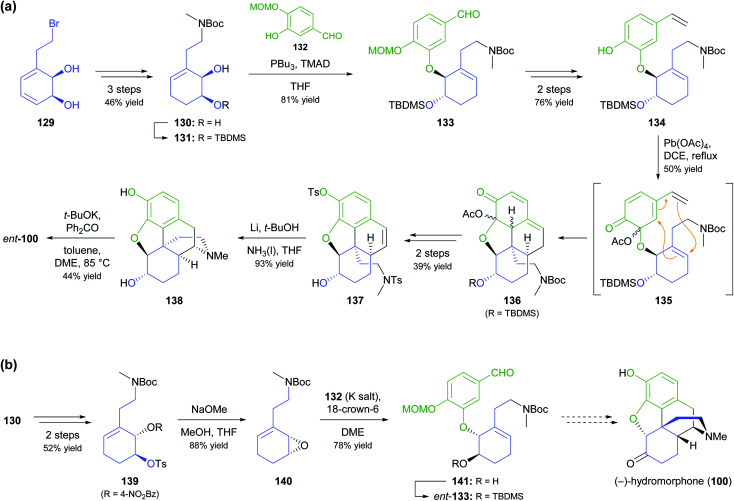
Synthesis of both enantiomers of hydromorphone (100) from TDO-derived building block 129: (a) transformation of 129 into the non-natural enantiomer, (+)-hydromorphone (*ent*-100), *via* Mitsunobu coupling, Diels–Alder cycloaddition, and N-centred radical cyclisation as key steps, (b) formal synthesis of the natural enantiomer, (−)-hydromorphone (100), using formation and ring-opening of epoxide 140 for stereoinversion.

As part of their ongoing efforts to develop practical syntheses of morphinan alkaloids,^77^ Hudlicky and co-workers have recently presented a novel chemo-enzymatic approach to both enantiomers of hydromorphone (100) starting from the TDO-derived building block 129 (Scheme 17a).^78^ The cornerstone of their synthetic strategy is the construction of ring B of the morphinan skeleton by a Diels–Alder reaction of intermediate 135, produced *in situ* by oxidative dearomatisation of the styrene derivative 134. This is followed by closure of ring D through radical cyclisation of tosylamide 137. The feasibility of this sequence was first demonstrated for the non-natural enantiomer, *ent*-100, using lead(iv) acetate as oxidant in the dearomatisation step and affording the target alkaloid in 2% overall yield over 12 steps from 129.^78*a*^ A follow-up study explored alternative oxidation methods, including the use of electrochemistry and of hypervalent iodine reagents, and found that Pb(OAc)_4_ can be replaced with the less environmentally problematic (diacetoxyiodo)benzene (DAIB), albeit at the cost of a reduced yield (20–30% for the cycloaddition product, compared to 50%).^78*b*^ In the same publication, the authors formally extended their synthetic strategy to the natural enantiomer, as they could demonstrate the conversion of diol 130 into *ent*-133*via* a five-step sequence (Scheme 17b).

**Scheme 18:**
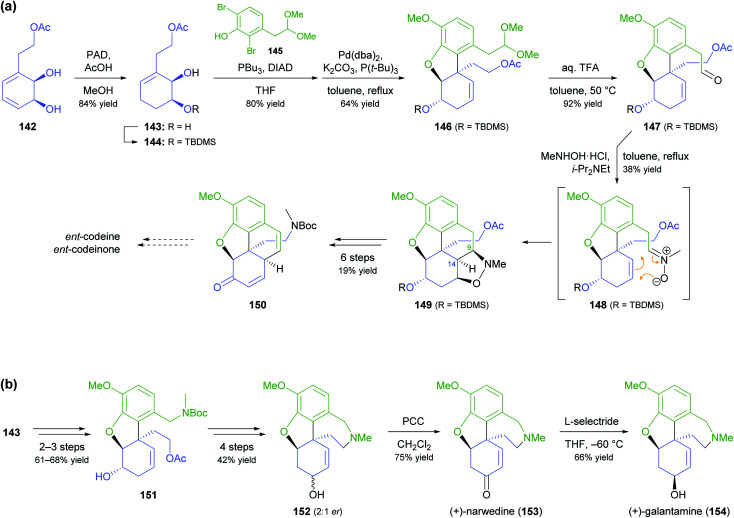
Examples for the use of TDO-derived building block 142 in alkaloid synthesis: (a) formal synthesis of *ent*-codeine and *ent*-codeinone *via* Mitsunobu coupling, Heck cyclisation, and nitrone–alkene (3 + 2) cycloaddition as key steps, (b) total synthesis of (+)-narwedine (153) and (+)-galantamine (154; non-natural enantiomers in both cases).

A more common, alternative strategy for morphinan synthesis is the construction of ring E by a sequence of Mitsunobu coupling and Heck cyclisation (*cf*. Schemes 6 and 18d of our previous review, ref. 33), followed by the successive closure of the remaining two rings. In recent years, the Hudlicky group has extensively explored this approach in synthetic studies that are based on building block 142 (Scheme 18a), a compound obtained in product titres of up to 5 g L^−1^ by the TDO-catalysed oxidation of phenethyl acetate.^79^ Partial reduction and silyl protection converted 142 into 144, which underwent Mitsunobu coupling with phenol 145 and subsequent Heck cyclisation to give the key intermediate 146, from which various routes to the complete morphinan scaffold were developed.^80^ In one study,^80^ conversion of 146 into the nitrone 148 triggered a (3 + 2)-cycloaddition that was intended to close ring B and establish the C9 amine stereocentre in a single operation. Unfortunately, the reaction produced isoxazolidine 149 with an incorrect C9,C14-stereoconfiguration, and an alternative cyclisation of a nitrile oxide followed by reduction gave the same result. Consequently, the authors decided to cleave the C9–N bond by a Hofmann elimination to enable installation of the correct stereochemistry at a later point in the synthesis. Within five more steps, they arrived at compound 150, which can be transformed into the non-natural enantiomers of codeine and codeinone by literature-known methods. Later studies established several options for converting intermediate 146 and related compounds into the non-natural (+)-forms of the semi-synthetic opioid analgesics oxycodone and 10-keto-oxycodone.^81^ Moreover, a structurally similar tricyclic intermediate, 151, served as precursor to the non-natural antipodes of the snowdrop alkaloids narwedine (153) and galantamine (154; Scheme 18b).^82^ The authors investigated two alternative Mitsunobu–Heck sequences, which both afforded 151 in good yield (61% over three steps, 68% over two steps) from diol 143. Elimination of the hydroxyl group, followed by iodoacetoxylation (Prévost reaction), reductive deiodination, and closure of the seven-membered D ring gave a 2 : 1 mixture of galantamine epimers (152), which was oxidised to (+)-narwedine (153) using pyridinium chlorochromate. Reduction of 153 with L-selectride at low temperature stereoselectively afforded (+)-galantamine (154).

**Scheme 19:**
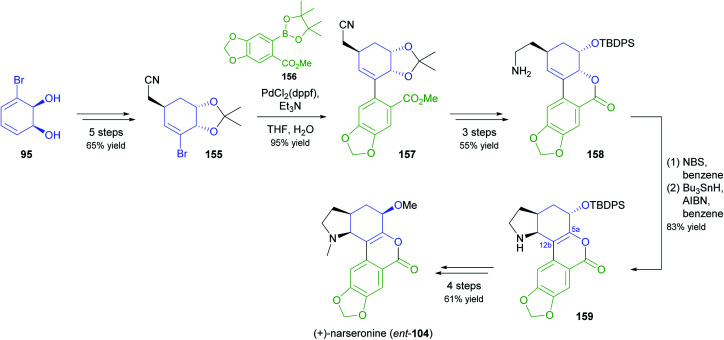
Synthesis of the non-natural enantiomer of narseronine (*ent*-104) from TDO-derived diol 95.

In an interesting example of stereodivergent synthesis, Banwell and co-workers prepared (+)-narseronine (*ent*-104) from the commercially available diol 95,^83^ after having previously used the same building block to obtain the (−)-enantiomer of this Amaryllidaceae alkaloid.^84^ The sequence followed to arrive at *ent*-104, however, resembles more closely the one developed in another synthetic endeavour of Banwell's group, the preparation of (+)-clividine:^85^ Diol 95 was converted into the vinylic bromide 155*via* a five-step sequence before being coupled with boronate 156 in a Suzuki reaction (Scheme 19). Protective-group manipulations and hydrogenation of the nitrile moiety led to primary amine 158, which underwent radical cyclisation to give the C5a–C12b-unsaturated compound 159. This outcome is in contrast to the clividine synthesis, where a similar reaction resulted in formation of the saturated product. The synthesis of (+)-narseronine was completed by inversion of the C5-stereocentre through an oxidation–reduction sequence and *N*,*O*-bismethylation. Other complex target molecules recently prepared by the Banwell group from building block 95 include non-natural analogues of the alkaloids galantamine and vindoline.^86^

### 2.3 Baker's yeast

**Scheme 20:**
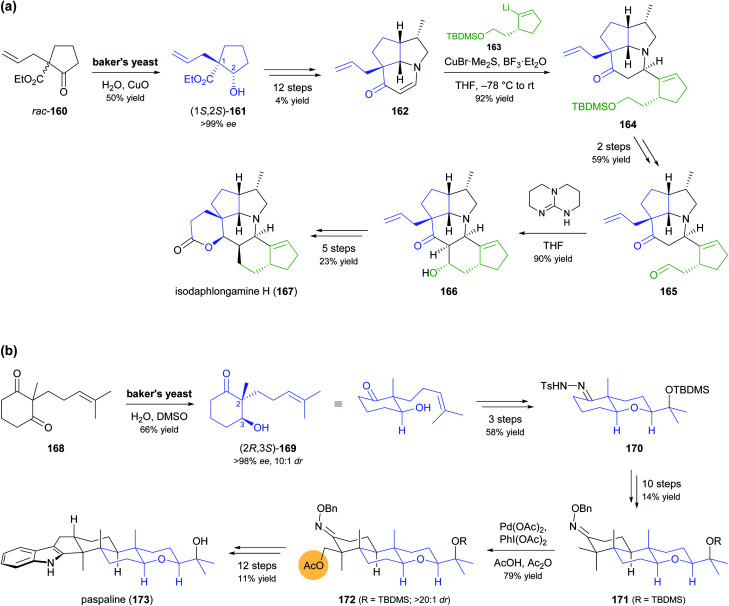
Recent application of baker's yeast in the total synthesis of alkaloids: (a) reductive kinetic resolution of β-ketoester *rac*-160 in the synthesis of isodaphlongamine H (167), (b) synthesis of paspaline (173) featuring reductive desymmetrisation of β-diketone 168 by baker's yeast and local desymmetrisation of 171 by Pd^2+^-catalysed diastereoselective C–H acetoxylation.

Despite the advances in molecular biology that have made recombinant enzymes broadly accessible,^87^ wild-type microorganisms remain relevant as biocatalysts. This is particularly true for baker's yeast, as it is cheap, easily available, and simple to use. In two recent studies, baker's yeast was employed for generating chiral building blocks that constituted the starting points for multi-step syntheses of highly complex alkaloids (Scheme 20).^88^ In both cases, the yeast reduced an α-substituted β-dicarbonyl compound, thereby controlling two vicinal chirality centres, one of which is quaternary. Hanessian and co-workers, in their synthesis of isodaphlongamine H (167), made use of the literature-known^89^ reductive kinetic resolution of β-ketoester 160 to prepare hydroxyester (1*S*,2*S*)-161 (50% yield, single isomer), which they elaborated into the enone 162 in a 12-step sequence (Scheme 20a). From 162, they constructed the hexacyclic framework of the target compound in 9 more steps, which include a copper(i)-mediated conjugate addition of vinyllithium reagent 163 and an intramolecular aldol addition.^88*c*^ The baker's yeast catalysed reductive desymmetrisation of diketone 168, inspired by similar reactions reported in literature,^90^ was used by Sharpe and Johnson in their total synthesis of paspaline (173), an indole diterpenoid from ergot fungi (Scheme 20b).^88*a*,*b*^ Reduction product 169, obtained in 66% yield, 10 : 1 *dr*, and >98% *ee*, served as basis for constructing the elongated, hexacyclic target compound with substrate-based stereocontrol of five additional chirality centres. Key transformations of the 27-step reaction sequence include an epoxidation–etherification cascade, an Ireland–Claisen rearrangement, a palladium(ii)-catalysed, diastereoselective C–H acetoxylation of intermediate 171, and a Gassmann indole synthesis.

## 3 Biocatalytic kinetic resolution, dynamic kinetic resolution, and deracemisation of alkaloids

The examples in the previous section have shown how a chiral building block prepared by an enzymatic transformation early in a synthetic sequence can be used to construct complex alkaloids with multiple chirality centres through substrate-based stereocontrol. For simpler target structures, in particular those that contain only a single stereogenic centre, it can often be more efficient to incorporate the asymmetric key step at a later point in the route. Biocatalysis has hence been used for the kinetic resolution, dynamic kinetic resolution, and deracemisation of alkaloids or advanced synthetic intermediates, with the majority of examples relying on lipases and amine oxidases.

### 3.1 Lipase-catalysed kinetic resolution and dynamic kinetic resolution

**Fig. 2:**
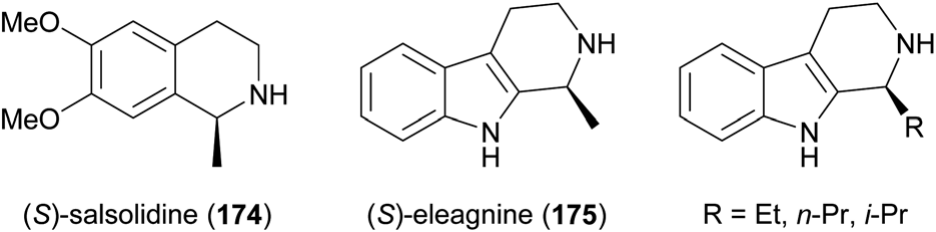
Structures of alkaloids obtained by kinetic resolution using lipase B from *Candida antarctica* (CAL-B).

Some simple alkaloids containing a chiral secondary amine moiety are amenable to kinetic resolution by lipase-catalysed *N*-acylation. Excellent results in this context were achieved for salsolidine (174) and eleagnine (175; Fig. 2) using lipase B from *Candida antarctica* (CAL-B) as biocatalyst and allyl phenyl carbonate as acyl donor.^91^ A strong dependence of enantioselectivity on reaction temperature and solvent was observed, but in methyl *tert*-butyl ether (MTBE) at 50 °C the kinetic resolution of *rac*-174 proceeded fast (50% conv. in 2.5 h) and with high selectivity (*E* >200), affording the acylated (*R*)-enantiomer in 97% *ee* along with unreacted (*S*)-174 in 99% *ee*. Interestingly, under identical conditions the acylation of *rac*-175 was prohibitively slow (47% conv. after one week), but substoichiometric addition of triethylamine (*ca*. 29 mol%) led to an increased reaction rate (50% conv. in 48 h) while maintaining an excellent enantioselectivity (*E* >200). In a follow-up study, the kinetic resolution of tetrahydro-β-carbolines with different C1 substituents (ethyl, *n*-propyl, isopropyl) was investigated, and excellent enantioselectivities were found in all cases.^92^

**Scheme 21:**
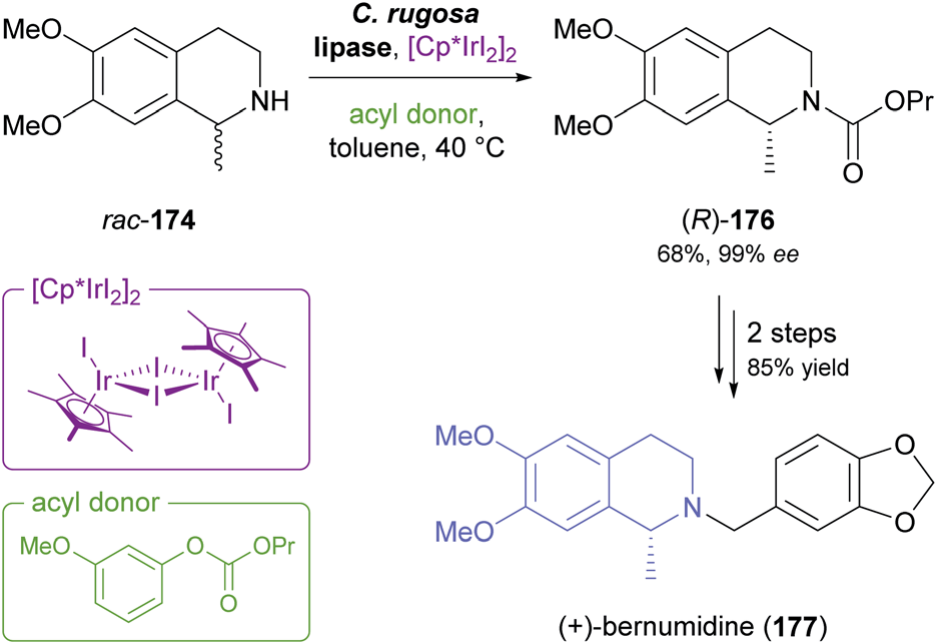
Chemo-enzymatic dynamic kinetic resolution of salsolidine (*rac*-174) and conversion of the resulting carbamate (*R*)-176 into (+)-bernumidine (177).

Recent progress in the mild racemisation of chiral secondary amines has also rendered the dynamic kinetic resolution (DKR) of simple alkaloids possible. In a recent example, the literature-known^93^ DKR of *rac*-174 using lipase from *Candida rugosa* in combination with an iridium(iii)-based racemisation catalyst was used to obtain the carbamate (*R*)-176 in 68% yield and 99% *ee* (Scheme 21).^94^ Hydrolysis of 176 gave (*R*)-salsolidine, which was further transformed into (+)-bernumidine (177), an alkaloid of *Berberis nummularia*.

In cases where the (dynamic) kinetic resolution of alkaloids by lipase-catalysed *N*-acylation is not possible, it may be achieved through the acylation of side-chain OH groups. Fülöp and co-workers have previously followed this strategy in their synthesis of crispine A, which used the kinetic resolution of the primary alcohol 180 as a key step (see Section 3.1 of our earlier review, ref. 33).^95^ More recently, the same authors have extended this method to tetrahydroisoquinolines with shorter C1 substituents – the natural product calycotomine (178, R = H) and its non-natural homologue 179.^96^ Kinetic resolution reactions were carried out using CAL-B as biocatalyst, vinyl acetate as acyl donor, and toluene as solvent in both batch and flow systems. Under flow conditions, (*R*)-178 (R = Boc) and its acylated (*S*)-enantiomer could be obtained in 99% *ee* at 50% conversion (*E* >200), while the kinetic resolution of 179 required the addition of triethylamine and sodium sulfate under batch conditions to reach a practically useful enantioselectivity (*E* = 88; Table 2). Three hydroxymethyl-substituted tetrahydro-β-carbolines (181–183) were subjected to CAL-B-catalysed kinetic resolution under similar reaction conditions, with excellent results in all cases (Table 2).^97^

**Table 2:** Kinetic resolution of primary alcohols using immobilised CAL-B

Substrate	Conditions	Products	*ee* [%]	*E* a	Ref.
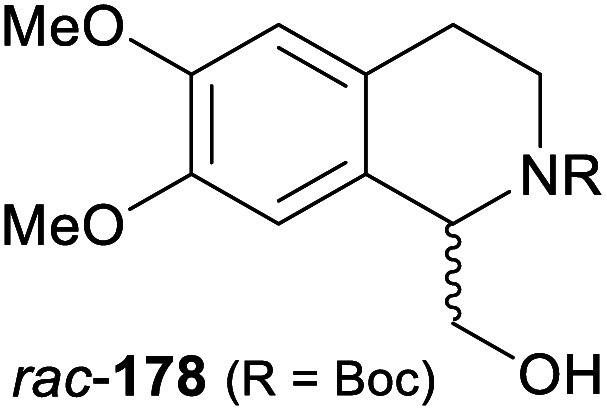	Toluene, 12 mM 178, 2 eq. vinyl acetate	(*R*)-178	99	>200	96*a*
		(*S*)-acetate	99		
	Flow (80 bar, 0.1 mL min^−1^), 60 °C				
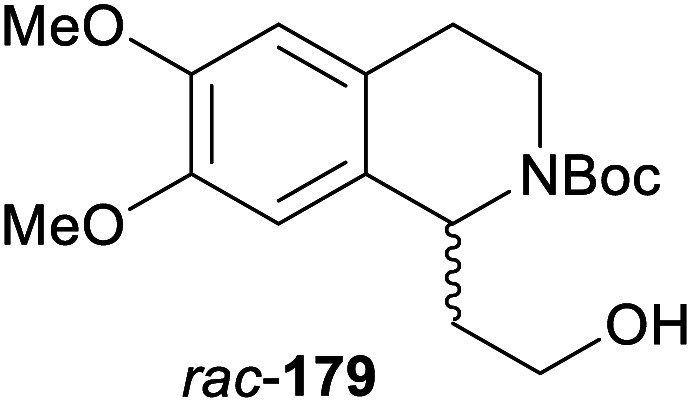	Toluene, 12 mM 179, 4 eq. vinyl acetate	(*R*)-179	30	88	96*b*
	Batch, Et_3_N, Na_2_SO_4_, 3 °C	(*S*)-acetate	97		
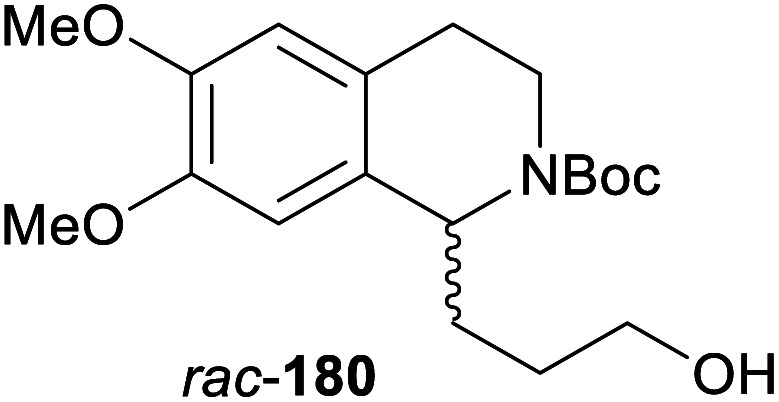	Toluene, 12 mM 180, 2 eq. vinyl acetate	(*R*)-180	*rac*	1	96*a*
	Flow (80 bar, 0.1 mL min^−1^), 60 °C	(*S*)-acetate	*rac*		
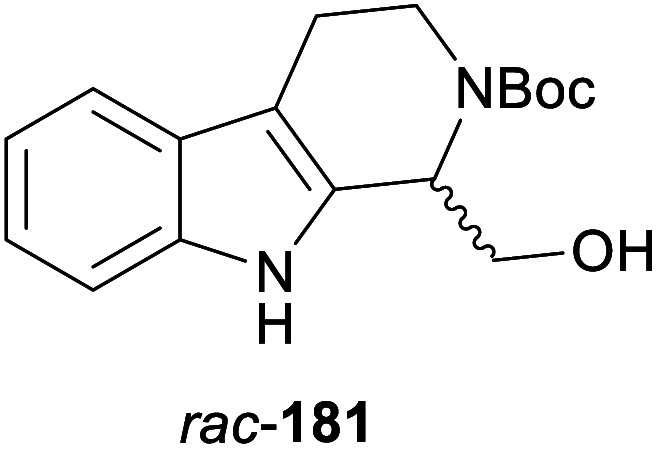	Toluene, 12.5 mM 181, 2 eq. Ac_2_O	(*R*)-181	98	>200	97
	Batch, 60 °C	(*S*)-acetate	98		
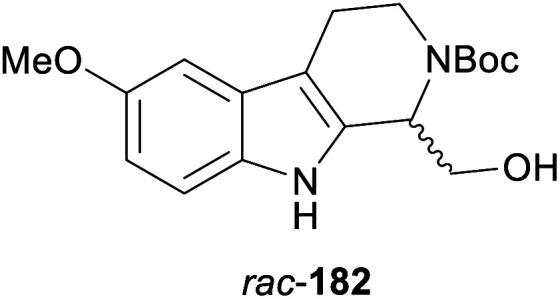	Toluene, 12.5 mM 182, 8 eq. Ac_2_O	(*R*)-182	98	>200	97
	Batch, 60 °C	(*S*)-acetate	98		
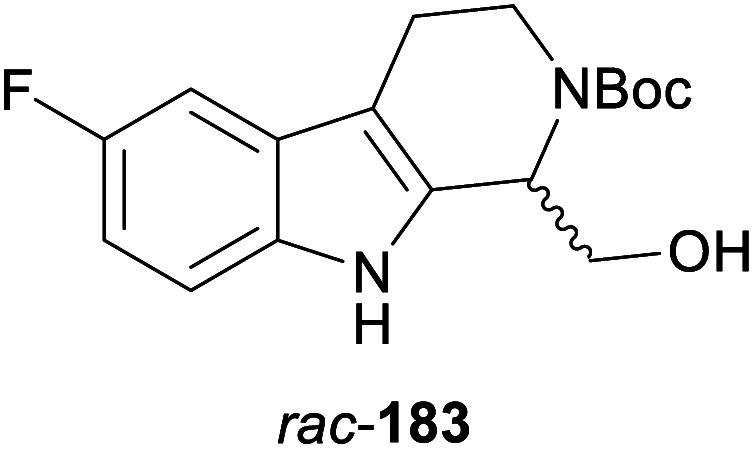	Toluene, 12.5 mM 183, 6 eq. Ac_2_O	(*R*)-183	96	>200	97
	Batch, 60 °C	(*S*)-acetate	98		

a Enantioselectivity, calculated from *ee* of substrate and product.

**Scheme 22:**
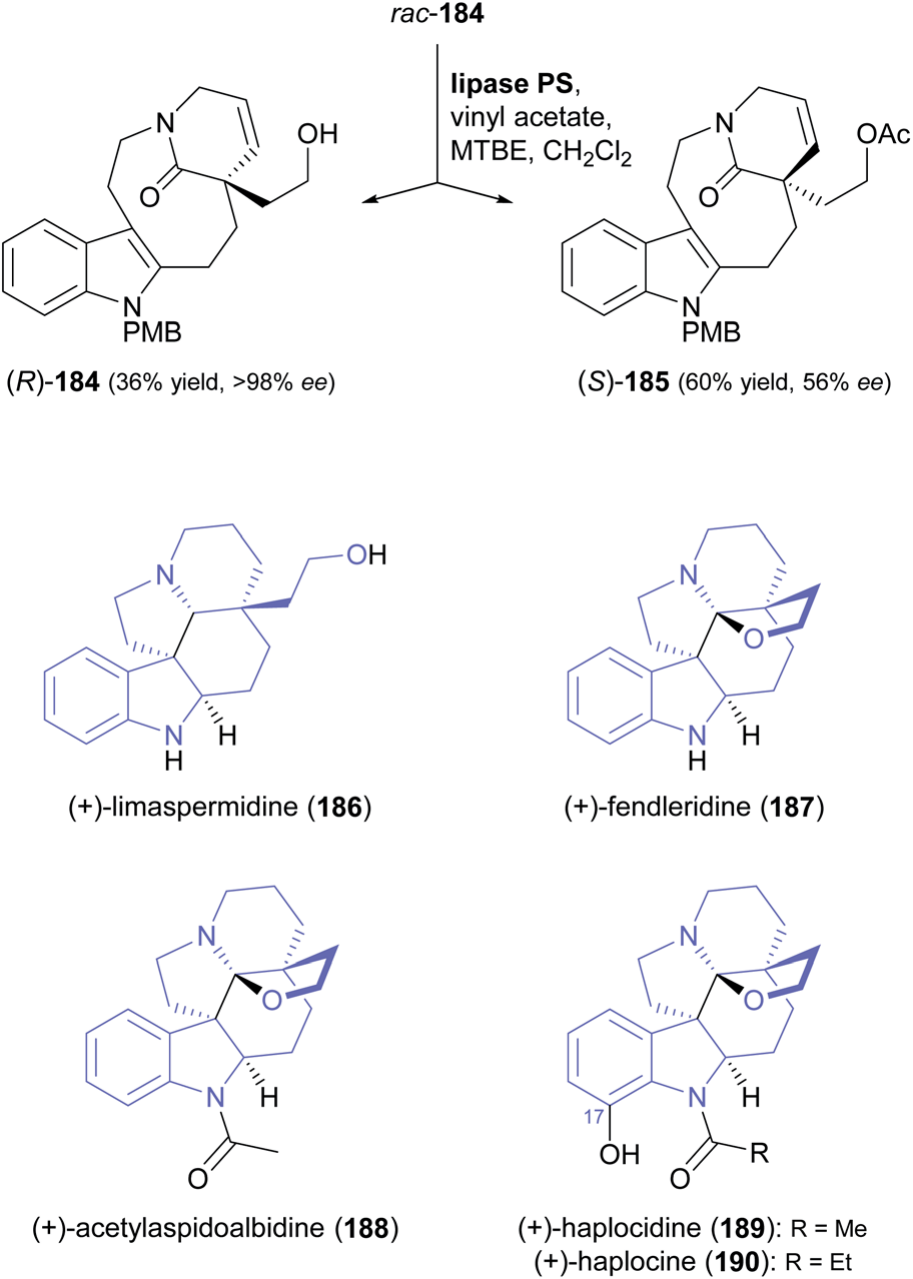
Kinetic resolution of lactam 184, and structures of alkaloids prepared from its (*R*)-enantiomer.

A particularly impressive example of a lipase-catalysed kinetic resolution of a primary alcohol constitutes the asymmetric key step in a recent synthesis of five *Aspidosperma* alkaloids:^98^ Lactam 184 (Scheme 22), containing a quaternary stereogenic centre in γ-position of the reactive hydroxyl group, was kinetically resolved using lipase PS^37^ and vinyl acetate. Although the enantioselectivity of this biotransformation was moderate (*E* = 22), the desired enantiomer (*R*)-(−)-184 could be obtained in 36% yield and >98% *ee* at 64% conversion. This advanced chiral intermediate was transformed into the alkaloids (+)-limaspermidine (186) and (+)-fendleridine (187) *via* electrophilic activation of the lactam moiety for cyclisation, followed by reduction and intramolecular hemiaminal formation, respectively. Acetylation of the secondary nitrogen atom led to (+)-acetylaspidoalbidine (188), while a palladium(ii)-catalysed, amide-directed *ortho*-oxidation provided access to the C17-hydroxylated natural products (+)-haplocidine (189) and (+)-haplocine (190).

**Scheme 23:**
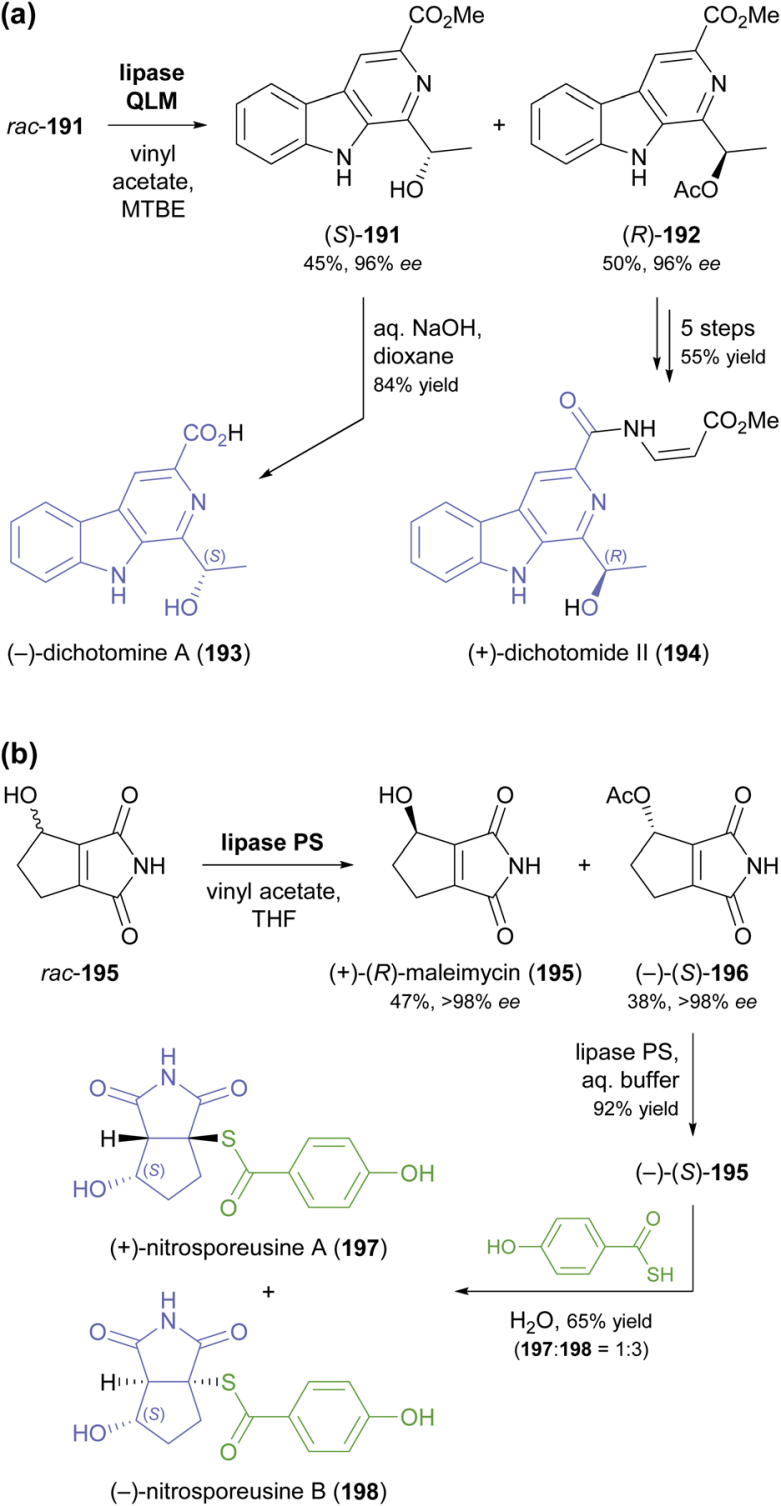
Lipase-catalysed kinetic resolution of *sec*-alcohol moieties in alkaloids: (a) kinetic resolution of *rac*-191 and its conversion into (−)-dichotomine A (193) and (+)-dichotomide II (194), (b) kinetic resolution of maleimycin (195) and its conversion into nitrosporeusines A and B (197, 198).

The lipase-catalysed acylation of secondary alcohols was used in two recent studies for the kinetic resolution of alkaloids and for elucidating the absolute configuration of natural products derived from them. The methyl ester of dichotomine A (191, Scheme 23a), a stitchwort alkaloid, was enantioselectively acetylated by lipase QLM^37^ to afford the (*R*)-acetate in 50% yield and 96% *ee* along with the (*S*)-alcohol in 45% yield and 96% *ee* (*E* = 194).^99^ Saponification of the methyl ester in (*S*)-191 gave (−)-dichotomine A (193), while aminolysis and a copper(i)-catalysed C–N-coupling – among other steps – were used to obtain (+)-dichotomide II (194) from (*R*)-192. The spectral characteristics and optical rotation of both target compounds matched those reported for material isolated from the natural source,^100^ confirming that the two natural products 193 and 194 have opposite absolute configuration. The kinetic resolution of maleimycin (195, Scheme 23b), an antibiotic metabolite of *Streptomyces showdoensis*, was accomplished using lipase PS^37^ and vinyl acetate in THF, giving (+)-195 in 47% yield and the corresponding (−)-acetate 196 in 38% yield, both in optically pure form (*ee* >98%, *E* >200). Hydrolysis of (−)-196 by lipase PS was used to access (−)-195. Both out of a synthetic interest and to elucidate the absolute configuration of (+)- and (−)-195, the authors then converted the two enantiomers into nitrosporeusines A and B (197 and 198), two alkaloids first isolated from an Arctic actinomycete.^101^ Interestingly, the non-natural enantiomer of maleimycin, (−)-(*S*)-195, was the one that led to the naturally occurring isomers of the nitrosporeusines.

### 3.2 Amine oxidases in chemo-enzymatic deracemisation systems

**Scheme 24:**
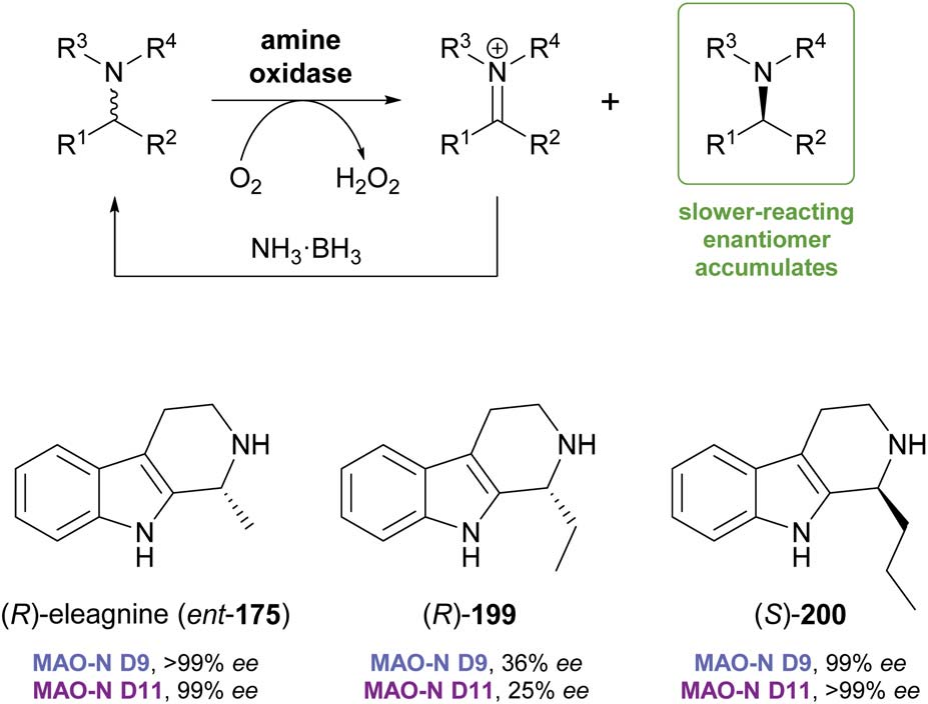
General reaction scheme for the ‘cyclic deracemisation’ of amines, and examples of tetrahydro-β-carbolines that have been deracemised using engineered variants of MAO-N.

Monoamine oxidases (MAOs) constitute a large class of flavoenzymes that oxidise amines to the corresponding imine or iminium species. Combining this transformation with a non-stereoselective reducing agent that converts the imine back to the racemic amine establishes a ‘cyclic deracemisation’, in which the less reactive amine enantiomer accumulates (Scheme 24).^102^ As discussed in our previous review,^33^ this deracemisation concept has been applied to a broad range of amines, including several alkaloids. In particular, Nicholas Turner's group at the University of Manchester has used directed evolution to develop several highly enantioselective variants of MAO from *Aspergillus niger* (MAO-N), which were shown to be applicable to the asymmetric synthesis of tetrahydroisoquinoline and tetrahydro-β-carboline alkaloids.^103^ Variants from this protein engineering campaign were recently tested with a broader set of substrates of these structural classes, with interesting stereochemical outcome.

Screening MAO-N variants D9 and D11 against eleagnine (175) and 10 non-natural tetrahydro-β-carbolines revealed a substrate-dependent switch in enantioselectivity for both variants (Scheme 24).^104^ While deracemisation of eleagnine and its ethyl analogue (199) afforded the (*R*)-enantiomers (in ≥99% and ≤36% *ee*, respectively), derivatives with longer-chain, branched, or cyclic C1-substituents led to the (*S*)-configured products. This observation was rationalised based on docking simulations, which indicated two different binding modes of the substrate enantiomers in the active site. (*S*)-Tetrahydro-β-carbolines with sterically demanding groups at C1 failed to dock in a productive conformation, explaining the switch in selectivity with increasing substituent size. The synthetic value of the method was demonstrated by preparative-scale (0.4 mmol) deracemisation of the propyl (200), butyl, isobutyl and phenyl derivatives, leading to the (*S*)-enantiomers in 90–99% *ee* and >85% yield.

**Scheme 25:**
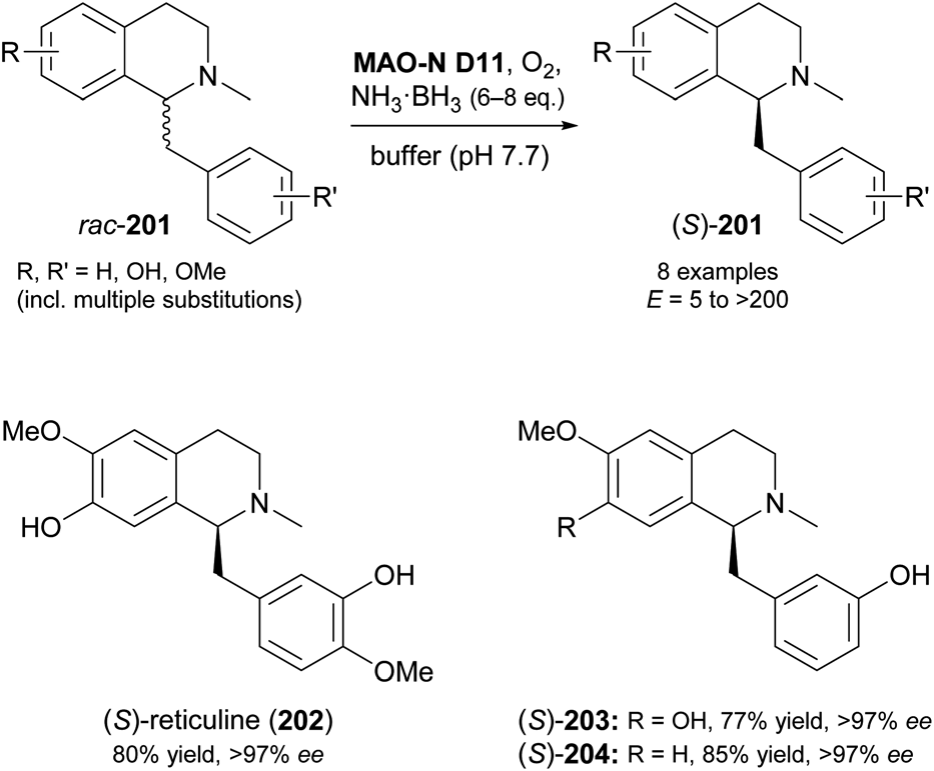
Deracemisation of benzylisoquinoline alkaloids 201 employing MAO-N variant D11 in combination with ammonia–borane as reducing agent.

In another study, MAO-N variants D5, D9 and D11 were screened against 15 benzylisoquinolines with diverse substitution patterns, and variant D11 showed activity on eight of these compounds (Scheme 25).^105^ Cyclic deracemisation led to the (*S*)-enantiomers in all cases, consistent with the earlier example of 1-phenyl-1,2,3,4-tetrahydroisoquinoline,^103^ but the enantioselectivity (*E*-value) of the MAO-N oxidation varied between 5 and >200 depending on the substrate structure. Docking simulations were again invoked to rationalise the experimental observations, revealing substituents on the substrates' isoquinoline ring to govern the enantioselectivity through steric interactions with active-site residues. After optimisation of biocatalyst production and reaction conditions, three deracemisations were run on a preparative scale (0.5 mmol), affording the natural product (*S*)-reticuline (202) and two non-natural analogues in 77–85% isolated yield and excellent optical purity (>97% *ee*).

**Fig. 3:**
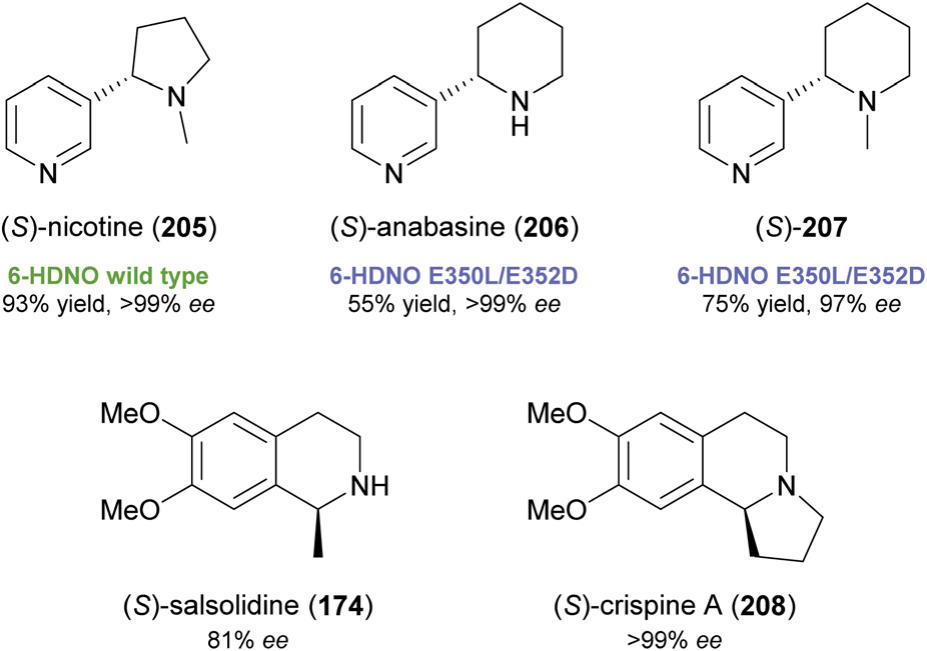
Alkaloids deracemised using 6-hydroxy-d-nicotine oxidase (6-HDNO).

While these recent findings demonstrate a certain stereochemical flexibility of engineered MAO-N variants, most deracemisation reactions using this enzyme afford (*R*)-configured products. A general access to (*S*)-amines *via* ‘cyclic deracemisation’ requires alternative amine oxidases with an enantioselectivity complementary to MAO-N. Turner and co-workers have identified 6-hydroxy-d-nicotine oxidase (6-HDNO) from *Arthrobacter nicotinovorans* as a suitable candidate in this regard and have engineered the enzyme for increased substrate tolerance.^106,107^ Wild-type 6-HDNO enabled the stereoinversion of (*R*)-nicotine to its naturally occurring (*S*)-enantiomer (205, Fig. 3), but was only active on eight of 34 tested amines. Analysis of a previously published model^108^ of enzyme–substrate interaction suggested residues for mutagenesis by a CAST (combinatorial active-site saturation test)^109^ approach, and a solid-phase colorimetric assay developed for MAO-N^110^ proved suitable for the fast screening of 6-HDNO variants. The mutation of only two active-site residues, E350L and E352D, resulted in a considerable expansion of the substrate scope (19 of 34 substrates accepted), which contrasts with the case of MAO-N, where up to 11 mutations were required to achieve a similar effect. Preparative deracemisation reactions afforded the nightshade alkaloids (*S*)-nicotine (205) and (*S*)-anabasine (206), as well as the *N*-methyl derivative of the latter (207), in high yields (55–93%) and excellent optical purities (97% to >99% *ee*). Moreover, the formation of (*S*)-salsolidine (174) and (*S*)-crispine A (208; non-natural enantiomer) from the racemates was demonstrated in analytical-scale reactions.

In a follow-up study, 6-HDNO was combined with an imine reductase (*cf.* Section 4) instead of ammonia-borane as reducing agent.^111^ The resulting two-enzyme system allowed the deracemisation of various 2-substituted pyrrolidines and piperidines, but alkaloids were not among the successful examples.

### 3.3 Oxidative kinetic resolution of benzylisoquinolines by berberine bridge enzyme

**Scheme 26:**
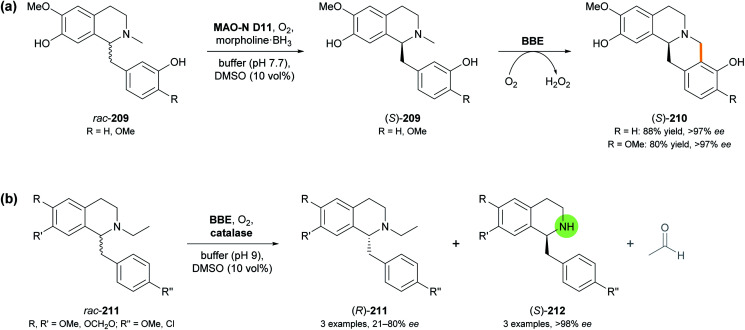
Recent applications of BBE in alkaloid synthesis: (a) transformation of *rac*-benzylisoquinolines 209 into (*S*)-berbines 210 by combining BBE with a monoamine oxidase variant (MAO-N D11) and a borane reducing agent, and (b) enantioselective dealkylation of *N*-ethyl-benzylisoquinolines 211 by BBE.

Berberine bridge enzyme (BBE, EC 1.21.3.3) is a flavoprotein oxidase that occurs in various alkaloid-producing plants, mainly from the families of *Papaveraceae* (poppy) and *Fumariaceae* (fumitory).^112^ Identified as a branch-point enzyme in the biosynthesis of benzylisoquinoline alkaloids, it catalyses the oxidative cyclisation of (*S*)-reticuline (209, R = OMe; Scheme 26) to (*S*)-scoulerine (210, R = OMe), forming the so-called ‘berberine bridge’. This transformation consumes O_2_ as stoichiometric oxidant and is enabled by an FAD cofactor that is bicovalently attached to the protein *via* a histidine and a cysteine residue.^113^ As discussed in our previous review,^33^ the efficient heterologous expression of BBE in *Pichia pastoris* has paved the way for detailed investigations of its structure and function, as well as for its exploitation as a biocatalyst. Racemic, non-natural benzylisoquinolines were transformed with high enantioselectivity, leading to kinetic resolution into (*S*)-berbines (tetrahydroprotoberberines) and the remaining (*R*)-configured substrates.

As a continuation of this work, and to overcome the intrinsic 50% yield limit of kinetic resolution, the BBE-catalysed oxidative C–C coupling was combined with ‘cyclic deracemisation’ of the benzylisoquinoline substrate using a monoamine oxidase variant (MAO-N D11) and a borane reducing agent (*cf*. Section 3.2).^114^ Critical for the success of this concept, which makes (*S*)-berbines 210 accessible from *rac*-benzylisoquinolines 209 (Scheme 26a), was the finding that MAO-N D11 oxidises several known BBE substrates with high (*R*)-selectivity, while being inactive towards the BBE reaction products. Morpholine-borane was chosen as reductant for the ‘cyclic deracemisation’ step, as it was found to be more compatible with the two oxidases than the much more commonly used ammonia-borane. The MAO- and BBE-catalysed transformations could be performed in step-wise or concurrent fashion with similar success, and the latter option was chosen for preparative-scale reactions (0.5 mmol of *rac*-209). Thus, the natural product (*S*)-scoulerine (210, R = OMe) and a close structural analogue were obtained in 80% and 88% isolated yield, respectively, and in optically pure form (>97% *ee*).

An attempt to extend the scope of BBE-catalysed C–C coupling from *N*-methyl to *N*-ethyl-benzylisoquinolines surprisingly revealed an enantioselective *N*-dealkylation activity of BBE. Instead of undergoing cyclisation to berbines, several *N*-ethyl-benzylisoquinolines (211) with varied substitution at the two aromatic rings were oxidatively cleaved to give the secondary amines (212) and acetaldehyde (Scheme 26b).^115^ Like the natural BBE activity, this dealkylation was highly selective for the (*S*)-enantiomer of the substrates (*E* >100), leaving the (*R*)-enantiomer untouched, but due to the relatively low reaction rate, maximum conversion (50%) was not attained in this kinetic resolution process. Consequently, the products (*S*)-212 were formed in >98% *ee*, while the optical purity of the remaining substrates (*R*)-211 varied between 21% and 80% *ee*, depending on the extent of conversion. Two preparative transformations on 80–170 mg scale afforded the dealkylation products in 16–24% isolated yield and >98% *ee*. Interestingly, one *N*-methyl-benzylisoquinoline that lacks the structural prerequisites for C–C-coupling by BBE (*N*-methyl analogue of 211 with R, R′, R′′ = OMe) also underwent dealkylation with high conversion (47%) and enantioselectivity (*E* >100), while two other tested *N*-methyl derivatives were not transformed at all.

## 4 Biocatalytic reduction of imines and iminium ions

**Scheme 27:**
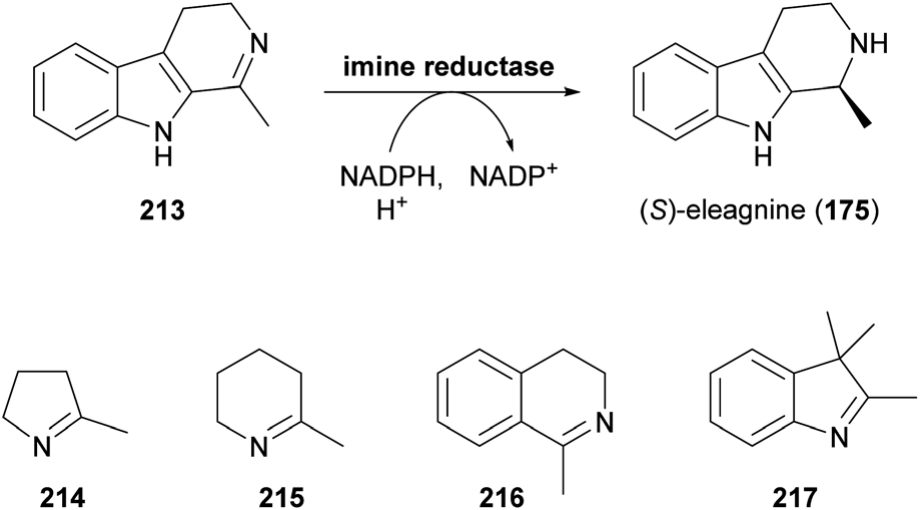
Formation of the alkaloid (*S*)-eleagnine (175) from the corresponding imine 213 by an imine reductase (IRED), and examples of non-alkaloid substrates of IREDs.

Since the publication of our last review on the role of biocatalysis in the asymmetric synthesis of alkaloids in 2013,^33^ a large, novel group of enzymes that show applicability for this purpose has emerged: Imine reductases (IREDs), as their name suggests, catalyse the reduction of a wide range of cyclic imines to the corresponding amines, consuming a reduced nicotinamide cofactor (usually NADPH) in the process (Scheme 27).^102*c*,116^ In addition, these enzymes are capable of catalysing the reductive amination of carbonyl compounds,^117^ a reactivity that has only very recently been harnessed for alkaloid synthesis.

While the enzymatic reduction of C=N double bonds is common in primary metabolism, this reaction has long remained elusive in a biocatalytic context.^116*d*^ Early examples have relied on whole microbial cells or cell-free extracts as catalysts, without identification of the responsible enzymes. This is also true for two studies in which the yeast *Saccharomyces bayanus* and a homogenisate of red Californian earthworms (*Eisenia foetida*) were used to obtain the (*S*)- and (*R*)-enantiomer, respectively, of the alkaloid eleagnine (175, *cf*. Scheme 27).^118^ Several non-natural tetrahydro-β-carbolines were also synthesised in these studies in 45–83% yield and 50% to >99% enantiomeric excess.

In 2010, Koichi Mitsukura and co-workers discovered imine reductase activity in two *Streptomyces* species, termed GF3587 and GF3546, by screening more than 680 microbial strains using 2-methyl-1-pyrroline (214, Scheme 27) as substrate.^119^ The responsible enzymes, which display complementary stereoselectivity, were identified soon afterwards^120^ and became the first characterised members of the IRED enzyme family, which now comprises more than 1400 known sequences.^121^ The discovery of this new enzyme class has sparked an immense interest from both academic and industrial research groups, and consequently, over the course of the last decade, many IREDs have been thoroughly studied regarding their substrate scope and stereoselectivity. The investigated substrate panels typically include small prochiral imines like the examples 214–217 shown in Scheme 27, but several IREDs have also proven useful for the asymmetric synthesis of simple alkaloids (Table 3). For instance, the hemlock neurotoxin (*R*)-coniine (218)^122^ was obtained in 90% isolated yield and >98% *ee* by gram-scale reduction of the corresponding imine, γ-coniceine, using the original (*R*)-selective IRED from *Streptomyces* sp. GF3587, heterologously expressed in *E. coli*.^123^*In situ* regeneration of the required NADPH cofactor was achieved *via* the metabolism of the expression host at the expense of d-glucose. (*R*)-Coniine is also accessible in high conversion and enantiomeric excess using the IREDs from *Nocardiopsis halophila* (*Nh*-IRED) and *Streptomyces kanamyceticus* (Q1EQE0, IRED-B),^124^ but interestingly no IRED that would afford the (*S*)-enantiomer (*ent*-218) with good stereoselectivity has been identified yet.

**Table 3:** Use of imine reductases (IREDs) for the production of a range of simple alkaloids from the corresponding imines

Product	IRED^a^	Conditions	Conv^b^. [%]	*ee* [%]	Ref.
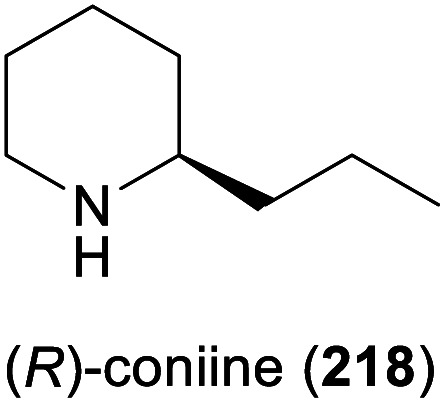	*Nh*-IRED	5 mM imine, 0.25 mg mL^−1^ IRED (purified enzyme), buffer not reported, 30 °C	90	82	124*a*
	IRED-B	50 mM imine, 2 mg mL^−1^ IRED (crude cell-free preparation), buffer (pH 7.5), 30 °C	98	>99	124*b*
	(*R*)-IRED GF3587	25 mM imine, whole cells expressing IRED (OD_600_ = 30), buffer (pH 7.0), 30 °C	>98 (90 isol.)	>98	123
(*S*)-coniine (*ent*-218)	*Bc*-IRED	5 mM imine, 0.25 mg mL^−1^ IRED (purified enzyme), buffer not reported, 30 °C	57	35	124*a*
	IRED-G	50 mM imine, 2 mg mL^−1^ IRED (crude cell-free preparation), buffer (pH 6.0), 30 °C	75	70	124*b*
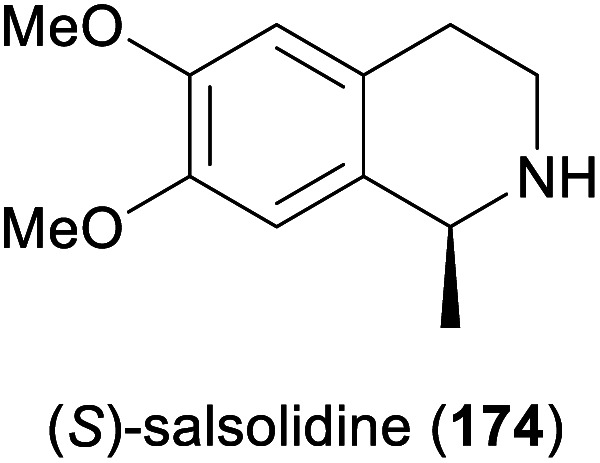	(*S*)-IRED GF3546	5 mM imine, whole cells expressing IRED (OD_600_ = 30), buffer (pH 7.0), 30 °C	92	>98	125*a*
	*Ao*-IRED (wt)	5 mM imine, 0.4 mg mL^−1^ IRED (purified enzyme), buffer (pH 7.5), 30 °C	50	79	125*c*
	IRED-M	10 mM imine, 2 mg mL^−1^ IRED (crude cell-free preparation), buffer (pH 7.5), 30 °C	>99	>99	125*e*
	*Sn*-IRED	100 mM imine, 10 mg mL^−1^ IRED (crude cell-free preparation), buffer (pH 7.0), 30 °C	>99 (76 isol.)	>99	125*d*
(*R*)-salsolidine (*ent*-174)	*Ao*-IRED (Y179A)	5 mM imine, 0.4 mg mL^−1^ IRED (purified enzyme), buffer (pH 7.5), 30 °C	15	>99	125*c*
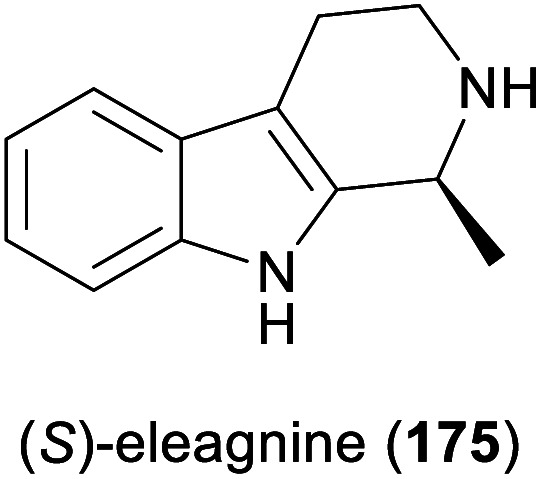	(*S*)-IRED GF3546	5 mM imine, whole cells expressing IRED (OD_600_ = 30), buffer (pH 7.0), 30 °C	>98	>98	125*a*
	IRED-M	10 mM imine, 2 mg mL^−1^ IRED (crude cell-free preparation), buffer (pH 7.5), 30 °C	92	>99	125*e*
	*Sa*-IRED	20 mM, 0.08 U mL^−1^ IRED (crude cell-free preparation), buffer (pH 7.0)	91	99	125*b*
(*R*)-eleagnine (*ent*-175)	*Ao*-IRED (wt)	5 mM imine, 0.4 mg mL^−1^ IRED (purified enzyme), buffer (pH 7.5), 30 °C	5	>99	125*c*
	IRED-D	10 mM imine, 2 mg mL^−1^ IRED (crude cell-free preparation), buffer (pH 7.5), 30 °C	27	78	125*e*
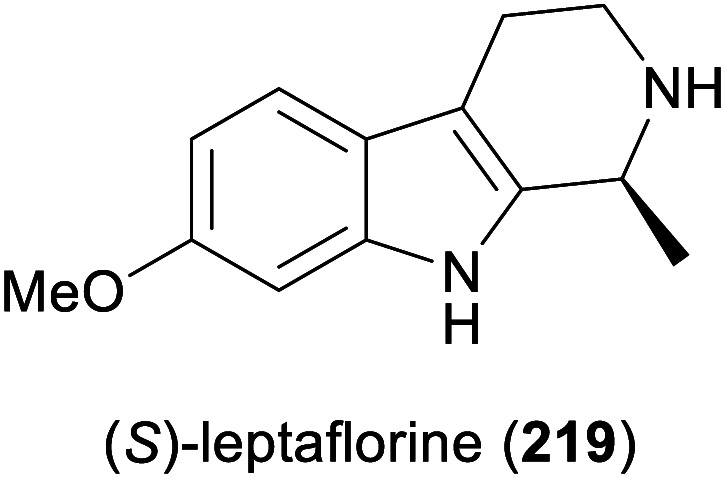	(*S*)-IRED GF3546	5 mM imine, whole cells expressing IRED (OD_600_ = 30), buffer (pH 7.0), 30 °C	50	>98	125*a*
	*Ao*-IRED (wt)	5 mM imine, 0.4 mg mL^−1^ IRED (purified enzyme), buffer (pH 7.5), 30 °C	15	79	125*c*
	IRED-L	10 mM imine, 2 mg mL^−1^ IRED (crude cell-free preparation), buffer (pH 7.5), 30 °C	88	>99	125*e*
(*R*)-leptaflorine (*ent*-219)	*Ao*-IRED (N241A)	5 mM imine, 0.4 mg mL^−1^ IRED (purified enzyme), buffer (pH 7.5), 30 °C	96	60	125*c*

a The IRED abbreviations are adopted from the original research articles and refer to the enzymes from the following source organisms: (*R*)-IRED GF3587, *Streptomyces* sp. GF3587; (*S*)-IRED GF3546, *Streptomyces* sp. GF3546; *Ao*-IRED, *Amycolatopsis orientalis*; *Bc*-IRED, *Bacillus cereus* BAG3X2; *Nh*-IRED, *Nocardiopsis halophila*; *Sa*-IRED, *Streptomyces aurantiacus* JA 4570; *Sn*-IRED, *Stackebrandtia nassauensis*; IRED-B, *Streptomyces kanamyceticus*; IRED-D, *Mesorhizobium* sp. L2C089B000; IRED-G, *Streptomyces rimosus* ATCC 10970; IRED-L, *Nocardia brasiliensis* ATCC 700358; IRED-M, *Saccharothrix espanaensis* ATCC 51144.

b Conversion according to GC or HPLC analysis. The values in parentheses are isolated yields of preparative-scale transformations.

Among the amines most commonly prepared by IRED catalysis are the isoquinoline alkaloid (*S*)-salsolidine (174) and the tetrahydro-β-carbolines (*S*)-eleagnine (175) and (*S*)-leptaflorine (219; Table 3). Several enzymes that are suitable for the synthesis of these natural products have been identified in screenings,^125^ and 174 has been prepared on a 150 mg scale (76% yield, >99% *ee*) using an IRED from *Stackebrandtia nassauensis* (*Sn*-IRED).^125*d*^ Access to the corresponding (*R*)-enantiomers is less straightforward, which reflects the general trend that (*S*)-selective IREDs seem to be more tolerant towards sterically demanding substrates than their (*R*)-selective counterparts.^125*e*^ Of particular note in this context is an IRED from *Amycolatopsis orientalis* (*Ao*-IRED), whose stereoselectivity is unusually sensitive to small variations in substrate structure.^125*c*^ The wild-type enzyme affords (*S*)-salsolidine (174) and (*S*)-leptaflorine (219) in moderate optical purity (79% *ee* in both cases), while eleagnine – which differs from 219 only by the absence of a methoxy substituent – is formed with low conversion (5%) but perfect (*R*)-selectivity (>99% *ee*). Even more surprisingly, imine 216 (Scheme 27) was reduced to the corresponding (*S*)-amine in 81–85% *ee* by fresh biocatalyst preparations, but storage of *Ao*-IRED at 4 °C for 24 h completely reversed its selectivity, leading to formation of the (*R*)-product in 98% *ee*. Neither extensive investigation of additives and reaction conditions nor the elucidation of the enzyme's crystal structure provided any explanations for this peculiar behaviour, but the crystal structure served as a basis for site-directed mutagenesis, which led to interesting (*R*)-selective enzyme variants. *Ao*-IRED Y179A produced optically pure (*R*)-salsolidine (*ent*-174), albeit at only 15% conversion, and variant N241A gave (*R*)-leptaflorine (*ent*-219) with near-complete conversion (96%) and 60% *ee*.

**Scheme 28:**
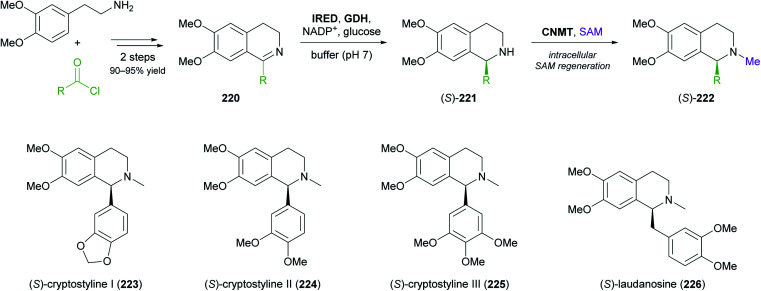
Three-enzyme cascade for the production of phenylisoquinoline and benzylisoquinoline alkaloids employing an IRED, d-glucose dehydrogenase (GDH) and coclaurine *N*-methyltransferase (CNMT).

More complex natural products than those shown in Table 3 have rarely been targeted with IRED-based routes. One notable example is a recent study by Xudong Qu and co-workers, who engineered an IRED from *Streptomyces aurantiacus* (IR45) for acceptance of sterically demanding substrates and used it in combination with d-glucose dehydrogenase and an *N*-methyltransferase to access phenyl- and benzylisoquinoline alkaloids (Scheme 28).^126^ Protein engineering of the imine reductase, identified in a previous study by the same group,^127^ was guided by docking simulations and led to two double variants (F190L–W191F and F190M–W191F) that reduced several phenyl- and benzyl-substituted imines 220 with fair activity (*k*_cat_ = 0.003–0.248 s^−1^) and excellent stereoselectivity (>99% *ee*). For preparative biotransformations, the appropriate IRED variant was either co-expressed with d-glucose dehydrogenase (GDH; for cofactor regeneration) and the third required enzyme, coclaurine *N*-methyltransferase (CNMT) from *Coptis japonica*, in a single *E. coli* host, or IRED and GDH were added to an *E. coli* culture expressing CNMT alone. In both systems, *S*-adenosyl methionine (SAM), the cofactor required by CNMT, was supplied and regenerated *in situ* by the expression host. Thus, (*S*)-cryptostylines I–III (223–225) and (*S*)-laudanosine (226) were obtained from the corresponding imines (50 mg L^−1^) in 93–98% isolated yield and in optically pure form (>99% *ee*).

**Scheme 29:**
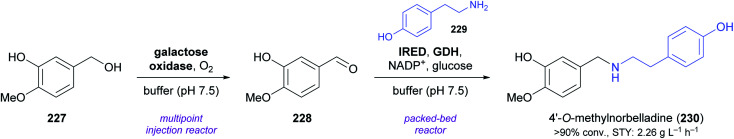
Synthesis of 4′-*O*-methylnorbelladine (230) in a three-enzyme continuous-flow system.

A very recent publication by Cosgrove, Flitsch, and co-workers marks the first time that an intermolecular reductive amination catalysed by an IRED was used in the context of alkaloid synthesis. As part of a larger study that explored multi-enzyme flow systems for the preparation of secondary amines, the authors prepared 4′-*O*-methylnorbelladine (230), a central biosynthetic precursor of *Amaryllidaceae* alkaloids,^128^ from tyramine (229) and isovanillyl alcohol (227) in an oxidation–amination cascade (Scheme 29).^129^ The oxidation of 227 to isovanillin (228) was performed by a galactose oxidase variant in a previously described^130^ multipoint injection reactor (MPIR). As soon as this continuous-flow biotransformation had reached a steady state, the MPIR effluent was mixed with a solution of 229, NADP^+^, and d-glucose, and pumped through a packed-bed reactor containing immobilised IRED and GDH. The two-step flow system produced a stable output of >90% 230 for four hours at 36 min total residence time, corresponding to a space-time yield (STY) of 2.26 g L^−1^ h^−1^.

Other recent examples of multi-enzyme reaction systems that involve imine reductases and bear some relevance to alkaloid synthesis are the combination of ene-reductases and IREDs for the preparation of cyclic amines with branched alkyl substituents, including non-natural coniine derivatives,^131^ and the combination of carboxylic acid reductases, transaminases and IREDs for the synthesis of diverse piperidines and pyrrolidines, including the natural product dihydropinidine (*cf*. Scheme 33a, Section 5.2).^132^

## 5 Biocatalytic asymmetric key C–C and C–N bond formation

The generation of molecular complexity from simple precursors is a key challenge in the synthesis of all natural products. In the context of alkaloid chemistry, carbon–carbon and carbon–nitrogen bond formation are of particular importance for constructing the scaffold of the target molecule. Biocatalytic methods that achieve these bond formations in stereoselective fashion hence have immense potential for enabling concise and often entirely novel synthesis routes to alkaloids. In our earlier review we have noted a shift of research focus towards such methods, and this trend has continued in recent years, as aldolases, transaminases, and Pictet–Spenglerases were employed in the preparation of a broad variety of alkaloids and non-natural alkaloid derivatives.

### 5.1 Aldolases

**Scheme 30:**
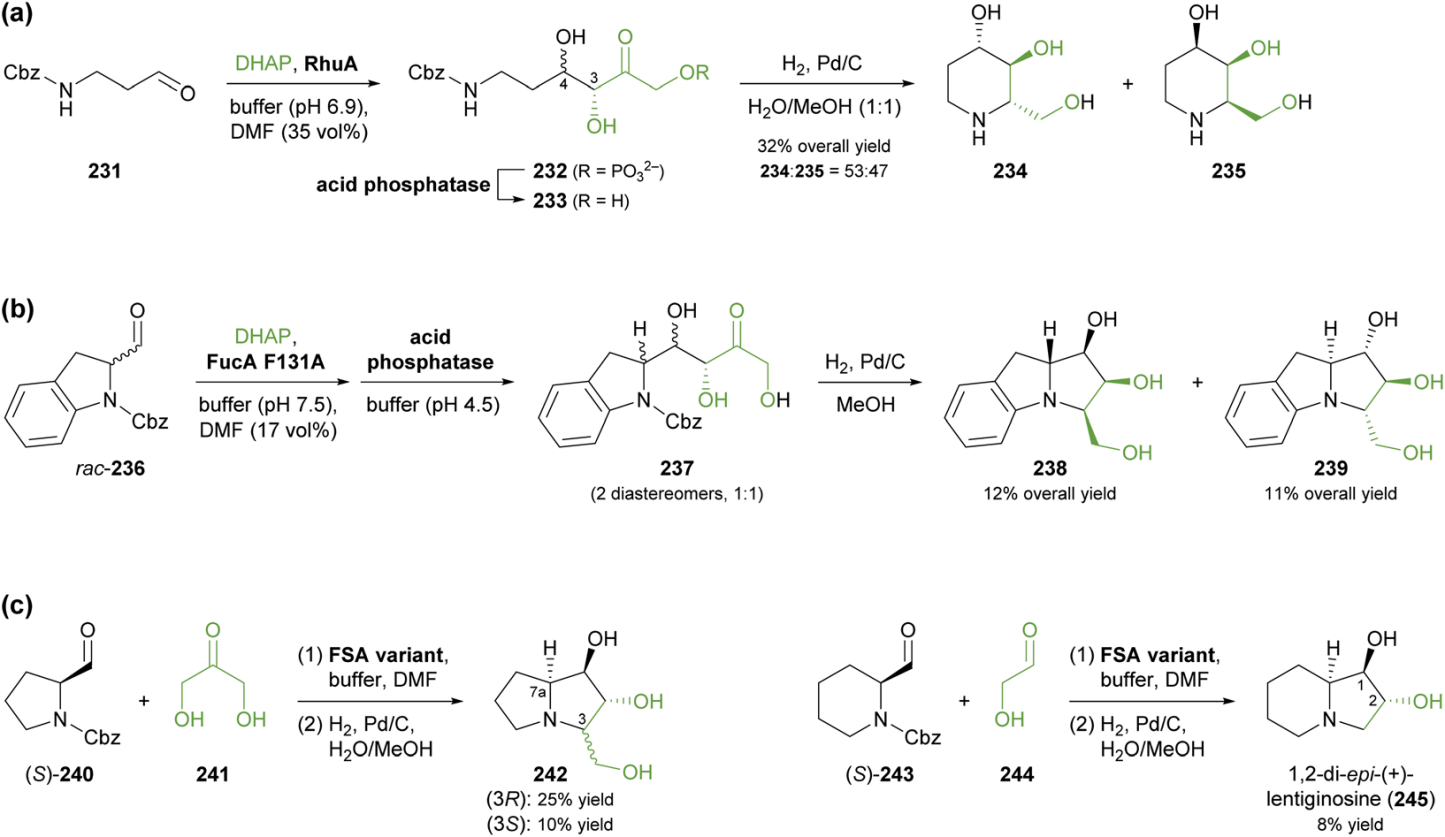
Recent applications of aldolases in the synthesis of non-natural iminocyclitols: (a) preparation of fagomine stereoisomers 234 and 235 using l-rhamnulose-1-phosphate aldolase (RhuA) from *Thermotoga maritima*, (b) synthesis of benzopyrrolizidines using an l-fuculose-1-phosphate aldolase (FucA) variant, and (c) preparation of (+)-hyacinthacine A2 stereoisomers 242 and 1,2-di-*epi*-(+)-lentiginosine (245) using variants of d-fructose-6-phosphate aldolase (FSA).

Aldolases are probably the most thoroughly studied class of C–C bond forming enzymes, and they have been applied in the preparation of a vast range of saccharides and other polyhydroxylated target molecules.^133^ In the field of alkaloid synthesis, enzymatic aldol additions to Cbz-amino-substituted aldehydes have been widely used in combination with catalytic hydrogenation to access iminocyclitols (‘aza-sugars’),^134^ as discussed in our previous review.^33^ Many of the approaches developed in this context gave rise to recent follow-up studies, which often focus on the investigation of novel aldolases or aldolase variants, and on the preparation of non-natural derivatives of known aza-sugars. For instance, a thermostable l-rhamnulose-1-phosphate aldolase (RhuA) from *Thermotoga maritima* was used to prepare a variety of non-natural piperidine, pyrrolidine, and pyrrolizidine iminocyclitols.^135^ The high solvent tolerance of this enzyme enabled an efficient aldol reaction between the poorly water-soluble aldehyde 231 and dihydroxyacetone phosphate (DHAP) in the presence of 35% (v/v) of DMF (Scheme 30a). The resulting aldol product 232 was dephosphorylated and subjected to catalytic hydrogenation to give compounds 234 and 235, which are diastereomers of the natural aza-sugar d-fagomine, in 32% combined yield and a 53 : 47 ratio. This stereochemical outcome indicates that the aldol addition proceeded with high (*R*)-selectivity at C3 of 232, but with low selectivity at the neighbouring C4 stereocentre. Similarly, the coupling of DHAP with racemic aldehyde 236 catalysed by variant F131A of l-fuculose-1-phosphate aldolase (FucA) from *E. coli* yielded, after dephosphorylation, two diastereomers of aldol 237 in a 1 : 1 ratio (Scheme 30b).^136^ Catalytic hydrogenation of this diastereomeric mixture afforded the non-natural benzopyrrolizidines 238 and 239, which proved separable by chromatography and were isolated in 12% and 11% overall yield, respectively.

In contrast to DHAP-dependent aldolases like RhuA and FucA, d-fructose-6-phosphate aldolase (FSA) from *E. coli* is known to accept non-phosphorylated carbonyl compounds as donor substrates.^133*c*^ This offers many advantages in chemo-enzymatic syntheses, including a lower cost of the donor, no requirement for phosphatase addition, and a generally broader donor scope, which expands the range of accessible products. Pere Clapés and co-workers recently carried out extensive protein engineering of FSA with the goal to improve its acceptance of α-substituted and α-cyclic aldehydes.^137^ The structure-guided engineering campaign started from previously reported single and double variants of FSA and explored the effect of various active-site mutations on aldol reactions using dihydroxyacetone (241), hydroxyacetone, and glycolaldehyde (244) as donor substrates. For the latter two donors, saturation mutagenesis of position R134 proved particularly effective and led to a panel of seven 4-point and 5-point variants capable of generating no less than 47 structurally diverse iminocyclitols. Of these, the ones most closely related to natural products are 7a-*epi*-(+)-hyacinthacine A2 [(3*R*)-242] and 7a,3-di-*epi*-(+)-hyanthacine A2 [(3*S*)-242], obtained *via* the aldol coupling of 241 and aldehyde (*S*)-240, and 1,2-di-*epi*-(+)-lentiginosine (245), prepared from 244 and aldehyde (*S*)-243 (Scheme 30c).

**Scheme 31:**
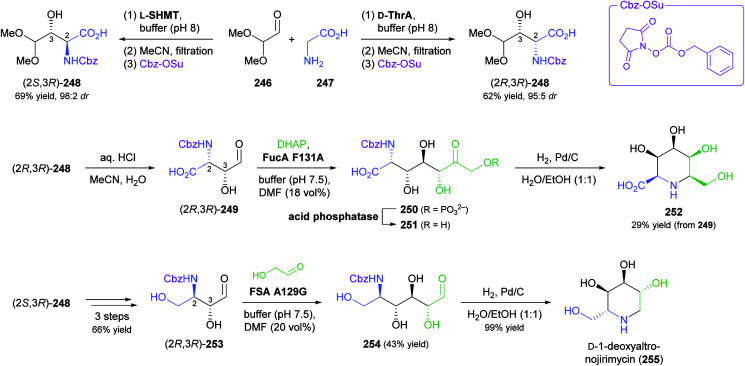
Chemo-enzymatic preparation of (2*S*,3*R*)-248 and its (2*R*,3*R*)-diastereomer using l-serine hydroxymethyltransferase (l-SHMT) and d-threonine aldolase (d-ThrA), respectively, and their further conversion into a pipecolic acid derivative (252) and the iminocyclitol d-1-deoxyaltronojirimycin (255) *via* a second aldol reaction.

While all the examples discussed so far rely on acceptor substrates obtained by chemical synthesis, two recent studies have established a stereodivergent access to iminocyclitols *via* two sequential aldolase reactions, one of which yields the acceptor substrate for the other. In the first case, a screening of glycine-utilising aldolases revealed l-serine hydroxymethyltransferase (l-SHMT) from *Streptococcus thermophilus* to give the threonine derivative (2*S*,3*R*)-248 in good yield (69%) and high stereoselectivity (98 : 2 *dr*) after Cbz protection, while d-threonine aldolase (d-ThrA) from *Achromobacter xylosoxidans* led to the (2*R*,3*R*)-diastereomer with comparable efficiency (62% yield, 95 : 5 *dr*; Scheme 31).^138^ Acidic hydrolysis of the dimethyl acetal in (2*R*,3*R*)-248 liberated aldehyde (2*R*,3*R*)-249, which underwent aldol coupling with DHAP, catalysed by FucA variant F131A, to yield compound 250 with full stereocontrol. The usual sequence of dephosphorylation and catalytic hydrogenation then afforded the polyhydroxylated pipecolic acid derivative 252 in good yield (29% from 249). Using epimeric (2*S*,3*R*)-248, aldolases other than FucA, and alternative donor substrates, a panel of 10 further pipecolic acid derivatives was also prepared. Moreover, both epimers of 248 could be transformed into the corresponding dihydroxyaldehydes 253 and further into (homo)iminocyclitols. For example, the aldol addition of (2*R*,3*R*)-253 and glycolaldehyde catalysed by FSA variant A129G gave – after hydrogenation – the iminocyclitol d-1-deoxyaltronojirimycin (255) in high yield (43% from 248) on multi-milligram scale.

**Scheme 32:**
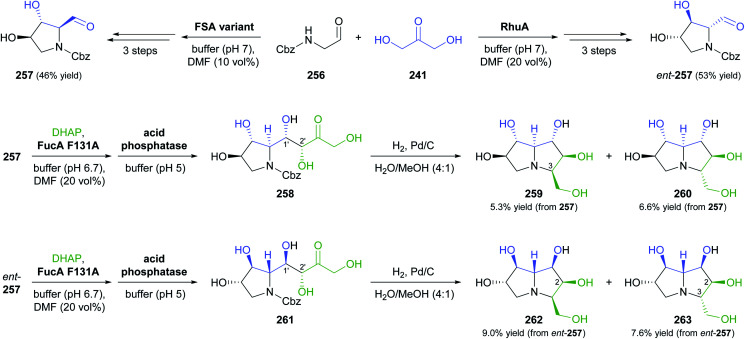
Chemo-enzymatic synthesis of *ent*-3-*epi*-casuarine (259), *ent*-casuarine (260), 2-*epi*-casuarine (262) and 2,3-di-*epi*-casuarine (263) *via* stereodivergent aldol reactions using d-fructose-6-phosphate aldolase (FSA), l-rhamnulose-1-phosphate aldolase (RhuA), and l-fuculose-1-phosphate aldolase (FucA).

In the second study, a related ‘double aldol’ approach gave access to four stereoisomers of the polyhydroxylated pyrrolizidine alkaloid casuarine.^139^ The aldol addition of dihydroxyacetone (241) to aldehyde 256, catalysed either by FSA variant A129S–A165G or by RhuA, and followed by hydrogenation, Cbz protection, and oxidation, yielded aldehyde 257 and its enantiomer, as described in a previous study (Scheme 32).^140^ The two aldehydes 257 and *ent*-257 were coupled with dihydroxyacetone phosphate by FucA variant F131A with (*R*)-selectivity at the 2′ carbon atom, irrespective of the aldehyde stereochemistry. The neighbouring C1′ stereocentre, on the other hand, was under substrate-based stereocontrol, leading to the exclusive formation of aldol adducts 258 and 261 after dephosphorylation. The final catalytic hydrogenation step was not stereoselective, but afforded epimeric mixtures that were separable by ion-exchange chromatography. Thus, *ent*-3-*epi*-casuarine (259), *ent*-casuarine (260), 2-*epi*-casuarine (262) and 2,3-di-*epi*-casuarine (263) were obtained in yields of 5.3–9.0% (from 257/*ent*-257).

### 5.2 Transaminases

**Scheme 33:**
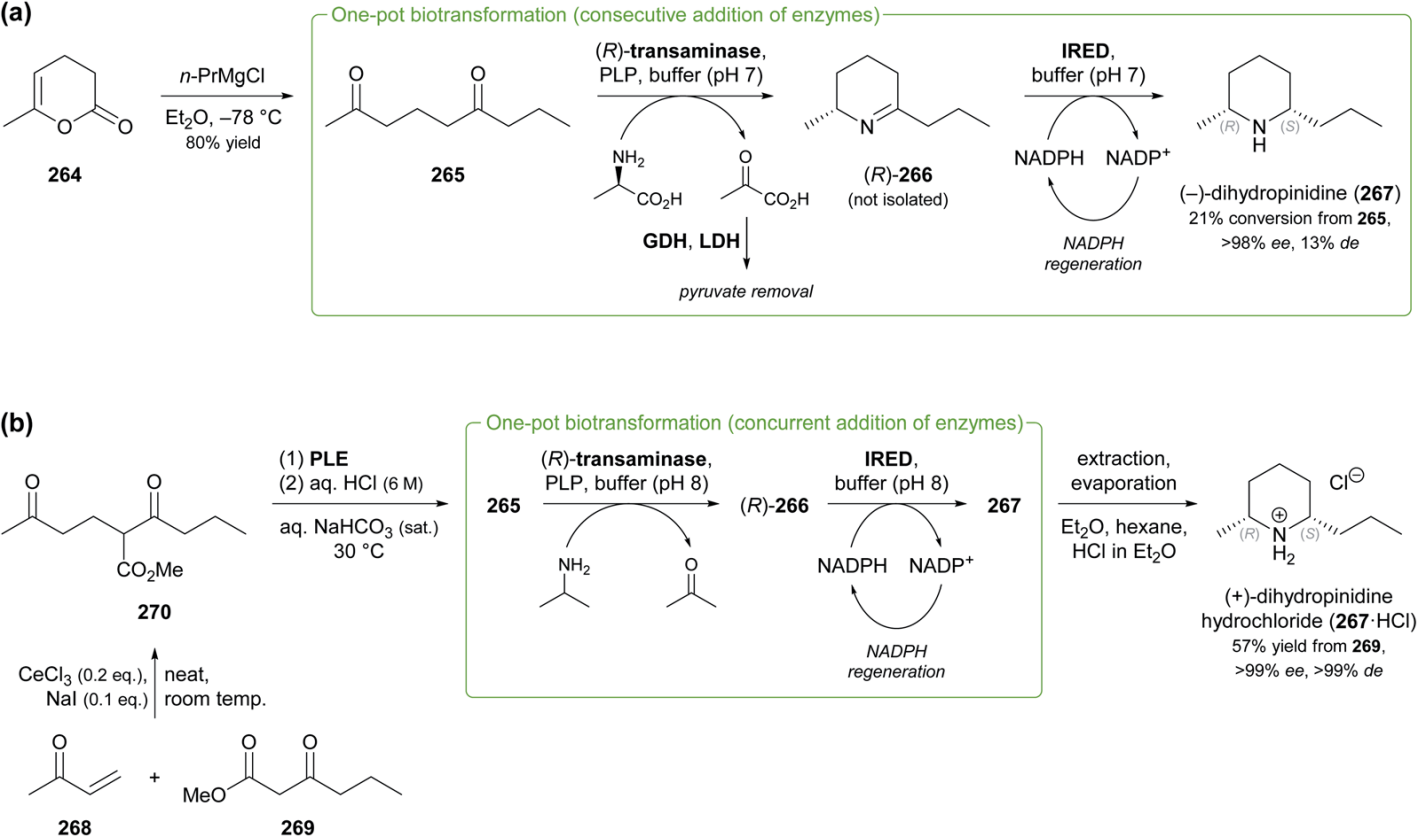
Chemo-enzymatic synthesis of dihydropinidine (267): (a) combination of transaminase and IRED biocatalysis in a one-pot system with consecutive addition of enzymes, (b) combination of transaminase and IRED biocatalysis in a concurrent one-pot system. Note that 267 and its hydrochloride (267·HCl) have opposite signs of optical rotation.

Transaminases are pyridoxal 5′-phosphate (PLP)-dependent enzymes that catalyse the reversible transfer of an amino group from an amine donor to a carbonyl acceptor. Over the course of the last decade, ω-transaminases (also referred to as amine transaminases, ATAs) have become the biocatalysts of choice for the synthesis of chiral primary amines from the corresponding prochiral ketones, a reaction with countless applications in academia and industry.^141^ Thanks to their broad substrate scope and their high stereoselectivity, ω-transaminases are also promising biocatalysts for the synthesis of alkaloids, and their use in this context has been significantly expanded since the publication of our previous review.^33^ There, we had discussed that the regioselective mono-amination of 1,5-diketones (*e.g.*, 265, Scheme 33) by transaminases leads to chiral, cyclic imines that can be reduced with excellent *cis*-selectivity by catalytic hydrogenation or with moderate *trans*-selectivity by LiAlH_4_ in the presence of Et_3_Al, affording 2,6-disubstituted piperidine derivatives.^142^ More recently, imine reductases (IREDs, *cf*. Section 4) were identified as an alternative to chemical reducing agents for the second step in this sequence, rendering a one-pot biotransformation of 1,5-diketones into disubstituted piperidines possible. This concept was first explored by Nicholas Turner and co-workers as part of a larger study aimed at the formation of diverse N-heterocycles from dicarbonyl precursors.^132^ Among several 2,6-disubstituted piperidines prepared in this work, the spruce tree alkaloid (−)-dihydropinidine (267) was obtained with 23% conversion, >99% *ee* and 13% *de*, *via* mono-amination of diketone 265 using a commercial, (*R*)-selective transaminase and subsequent reduction of imine (*R*)-266 using (*R*)-IRED GF3587 (Scheme 33a; *cf*. Section 4).^143^ Employing a commercial (*S*)-transaminase and the same IRED, the opposite enantiomer, *ent*-267, was formed with quantitative conversion and excellent stereoselectivity (>98% *ee*, 95% *de*). In both cases an established pyruvate removal system (LDH, GDH, glucose) was used to displace the transamination equilibrium, and the two enzymatic steps were performed sequentially in the same pot, adding the IRED after completion of the transaminase reaction. An even more integrated, multi-enzymatic approach to the same target molecule was later developed by our group (Scheme 33b).^124*b*^ In this work, the transamination of 265 was performed with isopropylamine as the amine donor, eliminating the need for auxiliary enzymes, and reduction of imine (*R*)-266 by an IRED from *Nocardiopsis alba* was carried out concurrently in the same reaction vessel. The synthesis of the substrate required for the biotransformation, diketone 265, was also modified: The previously used Grignard reaction of lactone 264 was replaced with a sequence of Lewis acid-catalysed Michael addition of compounds 268 and 269, ester hydrolysis by pig liver esterase (PLE), and acid-promoted decarboxylation. None of these steps required chromatographic purification of intermediates, making isolation of the target alkaloid by a single hydrochloride precipitation possible. Thus, 267·HCl was obtained in 57% overall yield (from 269) and stereoisomerically pure form (>99% *ee* and *de*) on a 5 gram scale. Similarly, 265 was transformed into *ent*-267·HCl (71% yield, >99% *ee*, >99% *de*) by an (*S*)-transaminase and an IRED from *Mesorhizobium* sp. on a 1 g scale.

**Scheme 34:**
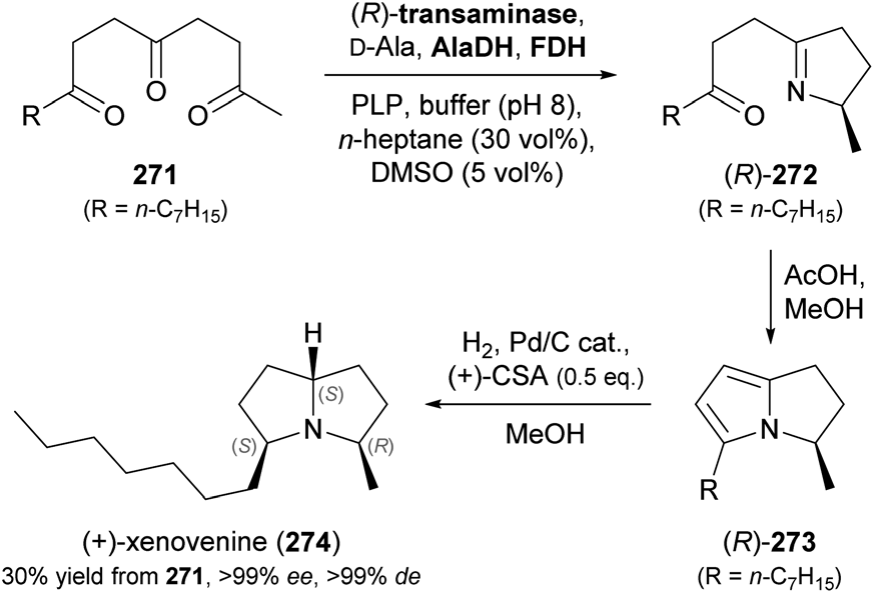
Chemo-enzymatic synthesis of (+)-xenovenine (274) *via* regioselective mono-amination of triketone 271, acid-catalysed pyrrole formation, and acid-promoted catalytic hydrogenation.

The original concept of combining a regioselective, enzymatic transamination with a diastereoselective, chemical reduction was recently extended to a triketone, thereby establishing access to both enantiomers of the ant venom alkaloid xenovenine (274, Scheme 34).^144^ Triketone 271, obtained in two steps from 2-heptylfuran, was aminated at the least sterically hindered of the three carbonyl groups by various transaminases, giving imine (*R*)- or (*S*)-272 with optical purities ranging from 54% to >99% *ee*. The use of *n*-heptane (30% v/v) and DMSO (5% v/v) as co-solvents was required to achieve high conversions of the poorly water-soluble substrate 271. Although imine 272 could be reduced to the target alkaloid by NaCNBH_3_, this reaction gave low yield, as large amounts of pyrrole 273 were formed as side product. The authors, therefore, decided to transform 272 into 273 quantitatively by treatment with acetic acid in methanol, followed by catalytic hydrogenation with camphorsulfonic acid (CSA) as additive. Despite attempts at optimisation, the hydrogenation could not be rendered completely stereoselective, but the obtained diastereomeric mixtures proved separable by chromatography. Performing the chemo-enzymatic sequence on 100 mg scale gave (+)-xenovenine (274) in 30% overall yield (from 271) and its enantiomer in 48% overall yield, both as single stereoisomers.

**Scheme 35:**
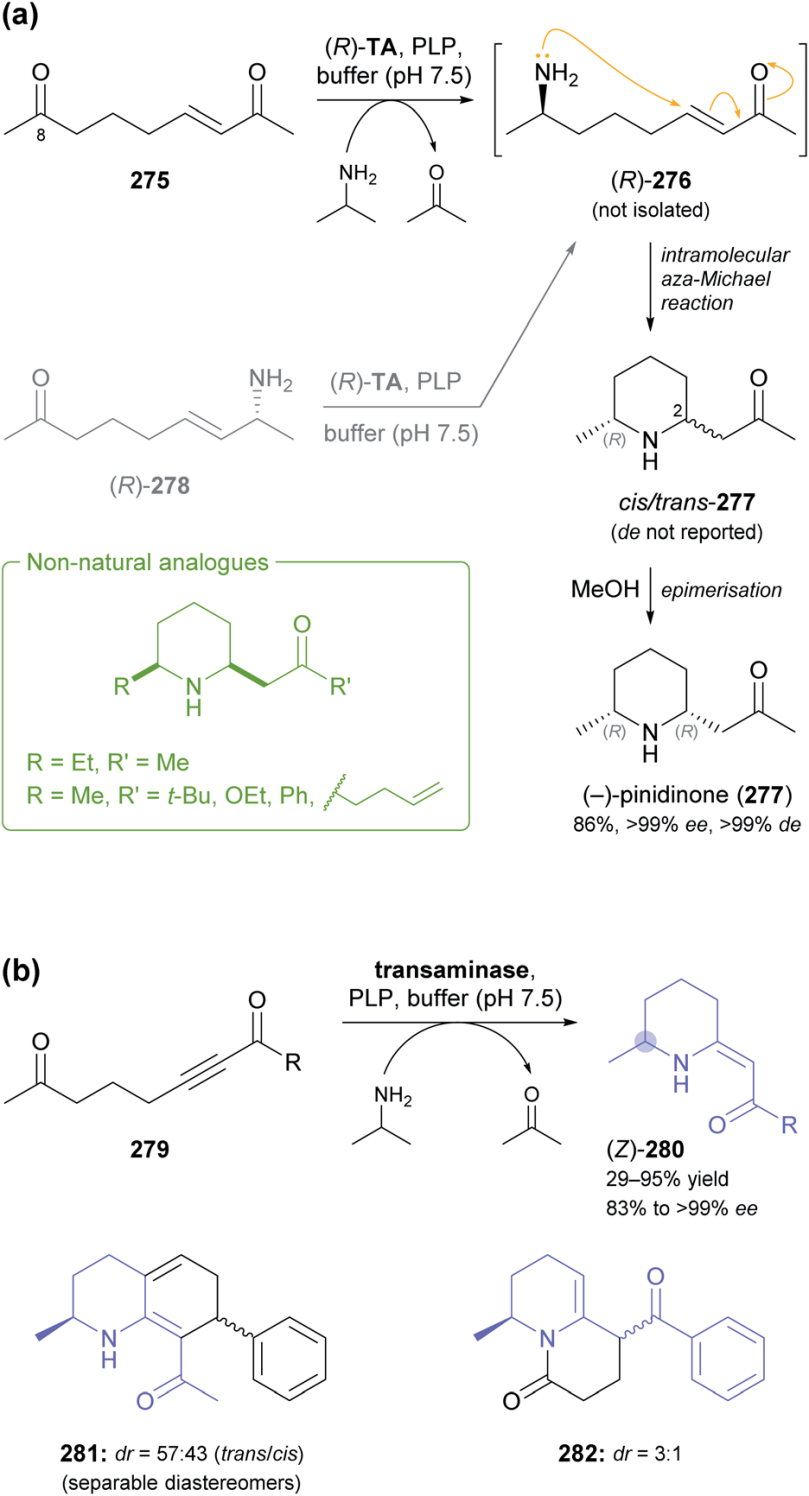
Reductive amination of ene-diones and yne-diones by transaminases (TAs) triggers an intramolecular aza-Michael reaction: (a) asymmetric synthesis of (−)-pinidinone (277) and several non-natural analogues, (b) preparation of (*Z*)-β-enaminones 280 and examples of annulation products that can be obtained from these building blocks.

In another elegant combination of enzymatic transamination with follow-up chemistry, the spruce alkaloid (−)-pinidinone (277) was obtained in essentially a single step from the diketone 275 (Scheme 35a).^145^ A commercial, (*R*)-selective transaminase converted 275 into the amine (*R*)-276, which underwent a spontaneous, intramolecular aza-Michael reaction to give 277 as a mixture of C2 epimers. This mixture equilibrated to the more stable *cis*-configuration by simple stirring in methanol for 24 h, affording (−)-pinidinone in 86% yield and optically pure form (>99% *ee* and *de*) on a half-gram scale. Several non-natural derivatives – including the opposite enantiomer of 277 – were obtained in analogous reactions. Notably, the transamination only required ≤2 equivalents of the amine donor, isopropylamine, as the Michael reaction withdraws amine 276 from the equilibrium. Since the authors did not expect the amination of 275 to be fully regioselective for the C8 carbonyl group, they independently synthesised the allylic amine (*R*)-278 and incubated it with the transaminase in the absence of amine donor. Complete conversion of 278 into 277 was observed, which demonstrates that the amine group can be transposed within the molecule. A recent follow-up study explored the extension of the transamination/aza-Michael sequence to yne-diones 279, giving β-enaminones (*Z*)-280 as products (Scheme 35b).^146^ Several examples of the general structure 280 were obtained in good yields (up to 95%) and optical purities ranging from 83% to >99% *ee*, with access to both enantiomeric forms through the use of stereocomplementary transaminases. The products were shown to be amenable to various annulation reactions, which afforded bicyclic compounds such as 281 and 282.

**Scheme 36:**
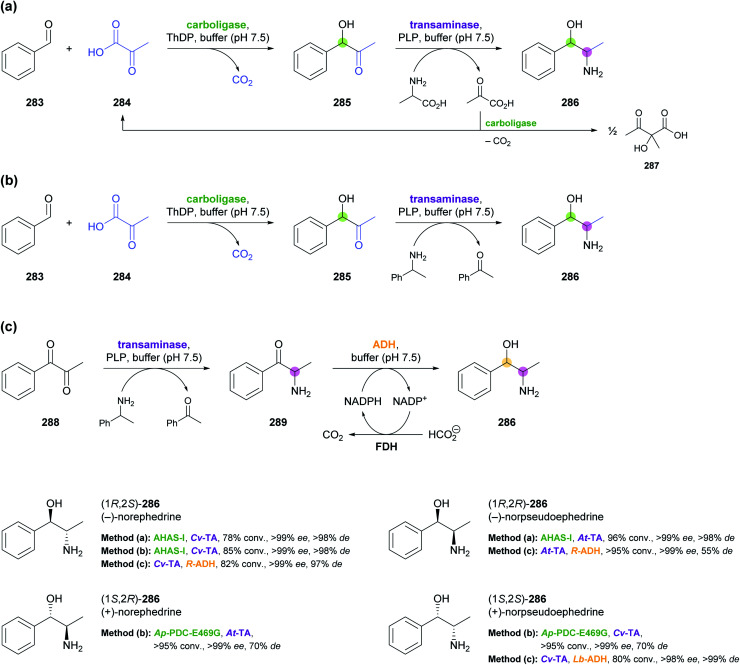
Biocatalytic two-step strategies for the synthesis of all four stereoisomers of nor(pseudo)ephedrine (286): (a) combination of carboligation and transamination, using l- or d-alanine as amine donor in a “recycling cascade”, (b) combination of carboligation and transamination, using (*R*)- or (*S*)-1-phenylethylamine as amine donor, and (c) combination of transamination and ketone reduction. Coloured circles represent stereogenic centres controlled by the enzyme shown in the same colour. For explanation of the enzyme abbreviations, see the main text.

Stereocomplementary transaminases have also been used to access all four stereoisomers of the *Ephedra* constituents^147^ norephedrine and norpseudoephedrine *via* different two-enzyme sequences. The first strategy, developed in 2013 by Dörte Rother and co-workers, relies on the thiamine diphosphate (ThDP)-dependent carboligase acetohydroxyacid synthase I (AHAS-I) from *E. coli* for formation of the hydroxyketone (*R*)-285 from benzaldehyde (283) and pyruvic acid (284, Scheme 36a) as the first step.^148^ Subsequently, the stereocomplementary transaminases from *Chromobacterium violaceum* (*Cv*-TA) and *Aspergillus terreus* (*At*-TA) convert (*R*)-285 into (−)-norephedrine [(1*R*,2*S*)-286] or (−)-norpseudoephedrine [(1*R*,2*R*)-286], using l- or d-alanine as amine donor. The two steps can be combined in one pot, whereby a consecutive addition of the enzymes is advantageous, as it avoids the undesired transamination of benzaldehyde. Thus, (1*R*,2*S*)-286 was formed in 78% conversion and (1*R*,2*R*)-286 in 96% conversion with excellent stereoselectivity in both cases (>99% *ee*, >98% *de*). Since the transaminase generates pyruvic acid as co-product, which is one of the two substrates for AHAS-I, the carboligation can be re-initiated by adding benzaldehyde after completion of the transamination step. This establishes what the authors call a “recycling cascade”, but the preparative value of this option was found to be limited. In a follow-up study, the same authors investigated a stereocomplementary carboligase and alternative reaction setups with the aim of extending the scope of the method to the remaining two isomers of 286 (Scheme 36b).^149^ The E469G variant of a pyruvate decarboxylase from *Acetobacter pasteurianus* (*Ap*-PDC-E469G) generated (*S*)-285 with near-complete conversion (95%) but only moderate *ee* (70%). Consequently, since the used transaminases accept both enantiomers of 285 as substrate, the final products (1*S*,2*R*)-286 and (1*S*,2*S*)-286 were obtained with quantitative conversion and >99% *ee* but only 70% diastereomeric excess. To overcome this limitation, the authors turned towards an entirely different approach that relies on alcohol dehydrogenases in addition to transaminases for the formation of the four 286 stereoisomers from diketone 288 (Scheme 36c).^149^ In this setup, the transamination of 288 using *Cv*-TA or *At*-TA as catalyst and 1-phenylethylamine as amine donor first leads to the (*S*)- and (*R*)-enantiomer, respectively, of amino ketone 289. Thereafter, the stereocomplementary alcohol dehydrogenases from *Lactobacillus brevis* (*Lb*-ADH) and *Ralstonia* sp. (*R*-ADH) were employed in combination with formate dehydrogenase (FDH) for cofactor regeneration to reduce 289 to the target compound 286. Inactivation of the transaminase by pH shift before ADH addition was necessary to avoid an undesired side reaction, the formation of 1-phenylpropane-1,2-diol. In this way, (1*S*,2*S*)-286 was generated in >98% *ee* and >99% *de* at 80% conversion, and an alternative entry to (1*R*,2*S*)-286 was also established (82% conv., >99% *ee*, 97% *de*). Generation of the (2*R*)-isomers by this approach was less successful: (1*R*,2*R*)-286 was formed in quantitative conversion but only 55% *de* by *At*-TA and *R*-ADH, while the combination of *At*-TA and *Lb*-ADH did not afford the expected (1*S*,2*R*)-product at all, producing (1*S*,2*S*)-286 instead (40% conv., >99% *ee*, 60% *de*). The authors propose an isomerisation of intermediate 289 as a possible explanation for this unexpected result.

Unfortunately, no preparative-scale biotransformations were conducted in either of these studies, but the synthesis of (−)-norephedrine [(1*R*,2*S*)-286] by the original carboligase–transaminase approach on a half-gram scale was independently reported by Yijun Chen and co-workers.^150^ They employed an (*R*)-selective pyruvate decarboxylase from *Saccharomyces cerevisiae* and an (*S*)-selective transaminase from *Vibrio fluvialis* as biocatalysts and obtained stereoisomerically pure (−)-norephedrine in 57% yield after column chromatography. In another independent report, AHAS-I and *Cv*-TA were used in the form of two individual *E. coli* whole-cell preparations to generate (1*R*,2*S*)-286 with 62% conversion, excellent stereoselectivity (>99% *ee* and *de*), and good reusability of the biocatalyst.^151^

**Scheme 37:**
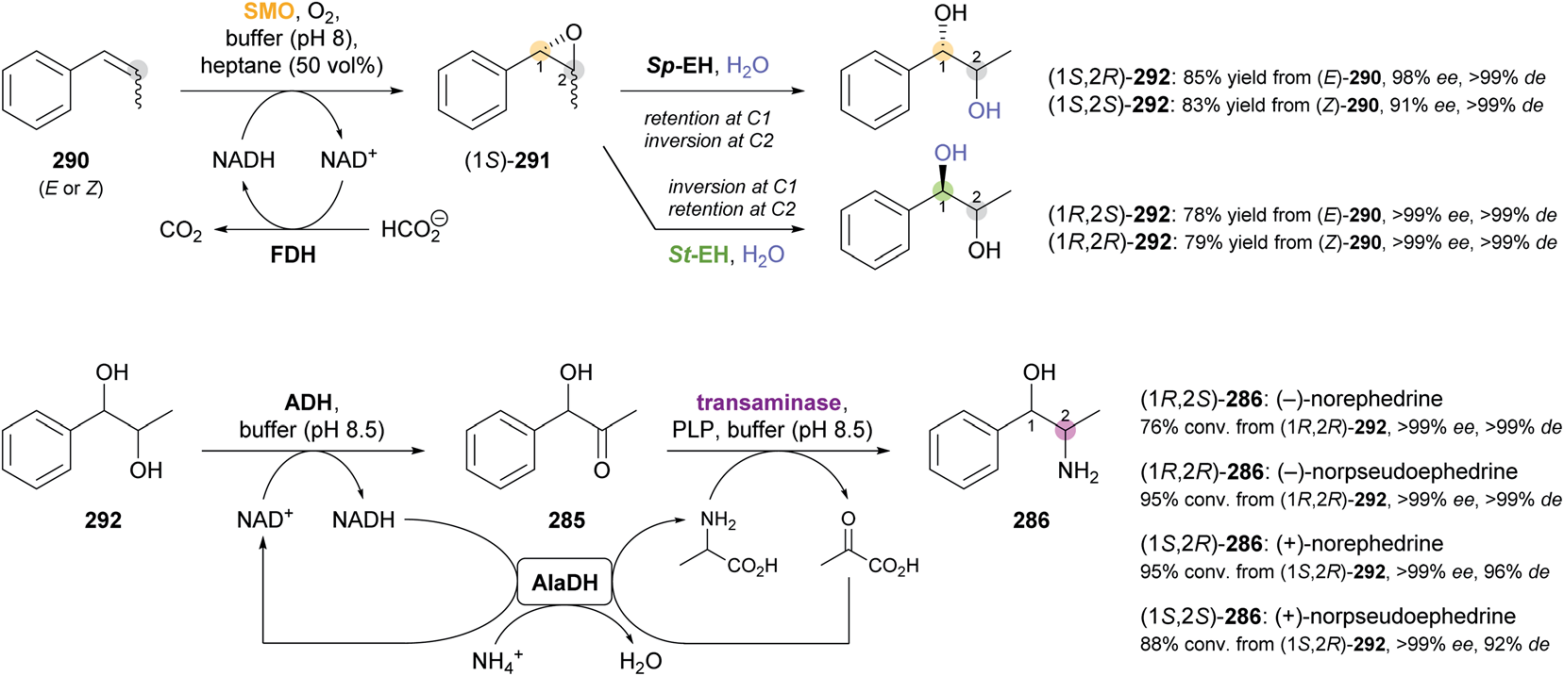
Asymmetric synthesis of all four stereoisomers of nor(pseudo)ephedrine (286) from β-methylstyrene (290) using a total of six enzymes in two biocatalytic one-pot reactions. Coloured circles represent stereogenic centres controlled by the enzyme shown in the same colour. For explanation of the enzyme abbreviations, see the main text.

An even more elaborate multi-enzymatic approach to all four stereoisomers of 286 was recently developed by Francesco Mutti and co-workers.^152^ Their synthetic sequence starts from β-methylstyrene (290), which was oxidised by a flavin-dependent styrene monooxygenase (SMO), used in combination with formate dehydrogenase (FDH) for NADH regeneration, to give the corresponding epoxide 291 (Scheme 37). This biotransformation proceeded with high (*S*)-stereoselectivity at the benzylic carbon atom (C1),^153^ while the configuration at C2 depended on the double bond geometry of 290: the (*E*)-isomer led to (1*S*,2*S*)-291, while the (*Z*)-isomer was converted into (1*S*,2*R*)-291. The epoxide was not isolated but directly transformed into the corresponding diol 292 by epoxide hydrolases from *Sphingomonas* sp. HXN-200 (*Sp*-EH) or *Solanum tuberosum* (potato; *St*-EH), thereby achieving further stereodivergence. The former enzyme catalysed water attack at C2 of the epoxide, resulting in retention of the C1 stereocentre and consequently to the formation of (1*S*,2*R*)-292 or (1*S*,2*S*)-292, depending on the C2 stereochemistry of 291. In contrast, epoxide hydrolysis by *St*-EH proceeded with attack at the benzylic carbon atom (C1), which led to stereochemical inversion at this position and afforded the (1*R*,2*S*)- or (1*R*,2*R*)-isomer of diol 292. The two biotransformations were carried out concurrently in one pot, using equal volumes of aqueous buffer and *n*-heptane as reaction medium. Thus, all four isomers of diol 292 could be accessed in good isolated yields (78–85%) and high optical purity (91% to >99% *ee*, >99% *de*) by proper choice of the starting material [(*E*)- or (*Z*)-290] and the epoxide hydrolase.^152*a*^ In a separate reaction step, the different stereoisomers of 292 were converted into nor(pseudo)ephedrines 286 by a redox-neutral biocatalytic network^154^ comprised of an alcohol dehydrogenase (ADH), a transaminase, and l-alanine dehydrogenase (AlaDH).^152*b*^ Since the stereoinformation at C2 of the diol is lost in this process, each isomer of 286 can in principle be accessed from two diastereomers of 292, but not all of the resulting cascade biotransformations proved equally effective. The best results were obtained when oxidation of (1*R*,2*R*)-292 by ADH from *Leifsonia* sp. or oxidation of (1*S*,2*R*)-292 by ADH from *Bacillus subtilis* was combined with transamination using enzymes from *Aspergillus terreus* (*R*-selective), *Bacillus megaterium* (*S*-selective) or *Chromobacterium violaceum* (*S*-selective). All four stereoisomers of 286 could be generated with high conversions (76–95%) and excellent optical purities (>99% *ee*, 92% to >99% *de*), whereby the authors ascribe the small limitations in *de* observed for the (1*S*)-products to an imperfect *ee* of diol (1*S*,2*R*)-292. For two representative cases, the entire sequence from β-methylstyrene (290) to 286 was carried out on a preparative scale (>100 mg), affording (+)-norpseudoephedrine (>99% *ee*, 94% *de*) and (−)-norephedrine (>99% *ee* and *de*) in isolated yields of 71% and 53%, respectively.

**Scheme 38:**
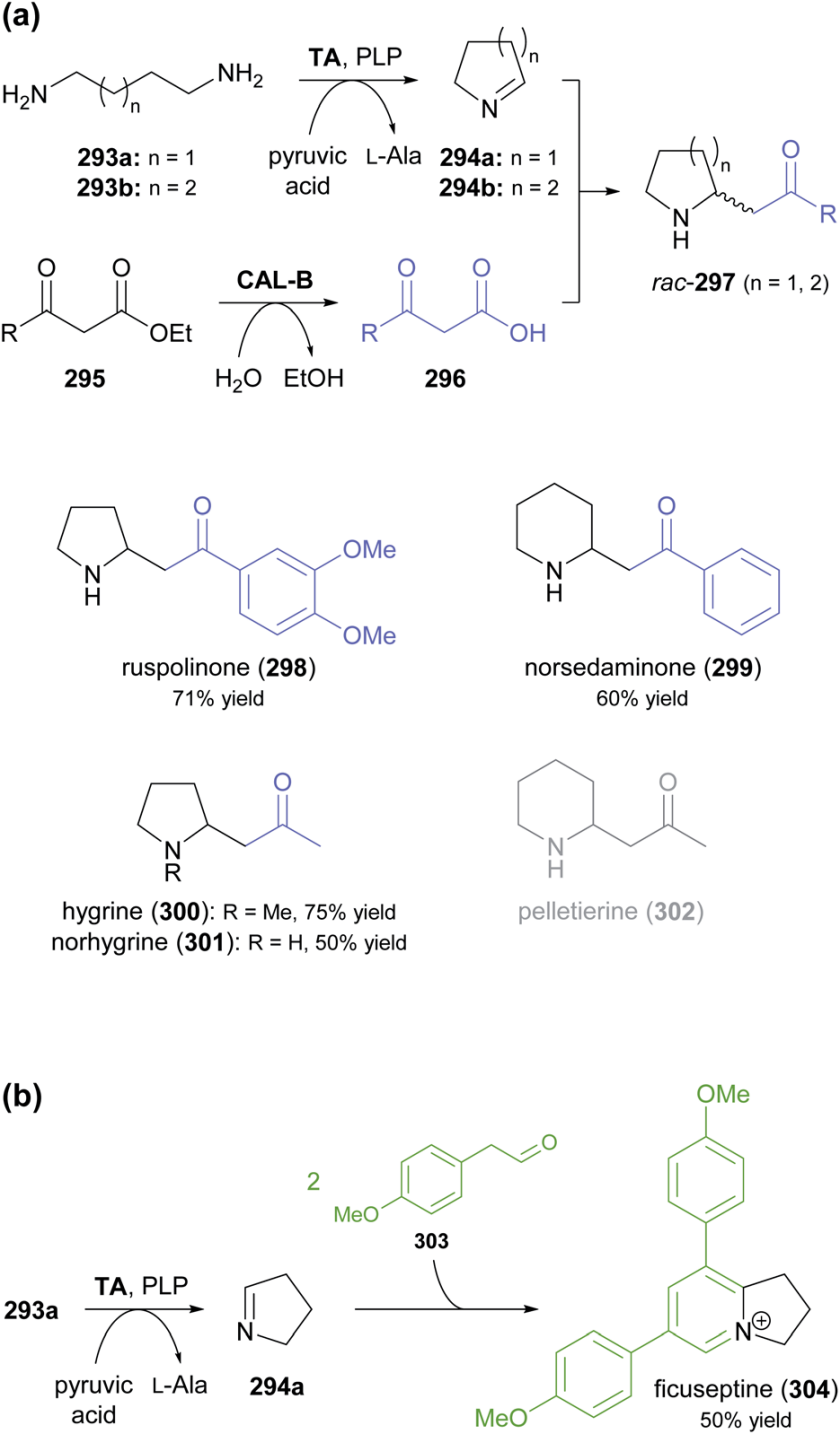
Use of putrescine transaminase in alkaloid synthesis: (a) biocatalytic formation of cyclic imines 294 from linear diamines 293 and decarboxylative Mannich reaction with *in situ* generated β-ketoacids 296 affords racemic pyrrolidine and piperidine alkaloids 297. (b) 1-Pyrroline (294a) formed by transaminase catalysis reacts with two molecules of aldehyde 303 to give the 2,3-dihydroindolizinium alkaloid ficuseptine (304).

While the main strength of transaminases is the stereoselective generation of α-chiral primary amines from prochiral ketones, as used in all examples discussed so far, the reverse reaction, *i.e.*, the deamination of amines, has also found application in alkaloid synthesis. A putrescine transaminase from *E. coli* was used to form the imines 1-pyrroline (294a) and 1-piperideine (294b) from the corresponding linear diamines 293 and pyruvic acid as amine group acceptor (Scheme 38a).^155^ The hydrolysis of β-ketoesters 295 by lipase B from *Candida antarctica* was carried out concurrently in the same reaction vessel and generated β-ketoacids 296, which combined with the imines 294 in a non-enzymatic, decarboxylative Mannich reaction that afforded racemic aminoketones 297 as final products. Biotransformations on 1.5 mmol scale gave the alkaloids ruspolinone (298), norsedaminone (299), hygrine (300), and norhygrine (301) in yields of 50–75%. Moreover, the formation of pelletierine (302) was confirmed analytically, but the compound could not be isolated in pure form. In a related approach, imine 294a was formed by putrescine transaminase and coupled with two molecules of aldehyde 303 to give the antibacterial and antifungal *Ficus* constituent ficuseptine (304) in 50% isolated yield (Scheme 38b).^156^

Additional examples of the use of transaminases in alkaloid synthesis, encompassing both amination and deamination reactions, come in the form of multi-enzyme sequences centred on Pictet–Spenglerases. These approaches are discussed in the next section.

### 5.3 Pictet–Spenglerases

In our previous review from 2013, we described the Pictet–Spenglerases as an emerging class of biocatalysts with vast application potential in the asymmetric synthesis of alkaloids.^33^ The progress achieved since then, however, has even exceeded our expectations. Large efforts were made to further investigate the substrate scope of the two established Pictet–Spenglerases, norcoclaurine synthase (NCS) and strictosidine synthase (STR), and to engineer variants with improved activities, which was often made possible by advancements in the mechanistic understanding of these enzymes.^157^ In particular, the non-natural substrate scope of NCS was expanded greatly, and novel synthetic approaches to natural alkaloids using NCS and STR were developed.

#### 5.3.1 Norcoclaurine synthase

**Scheme 39:**
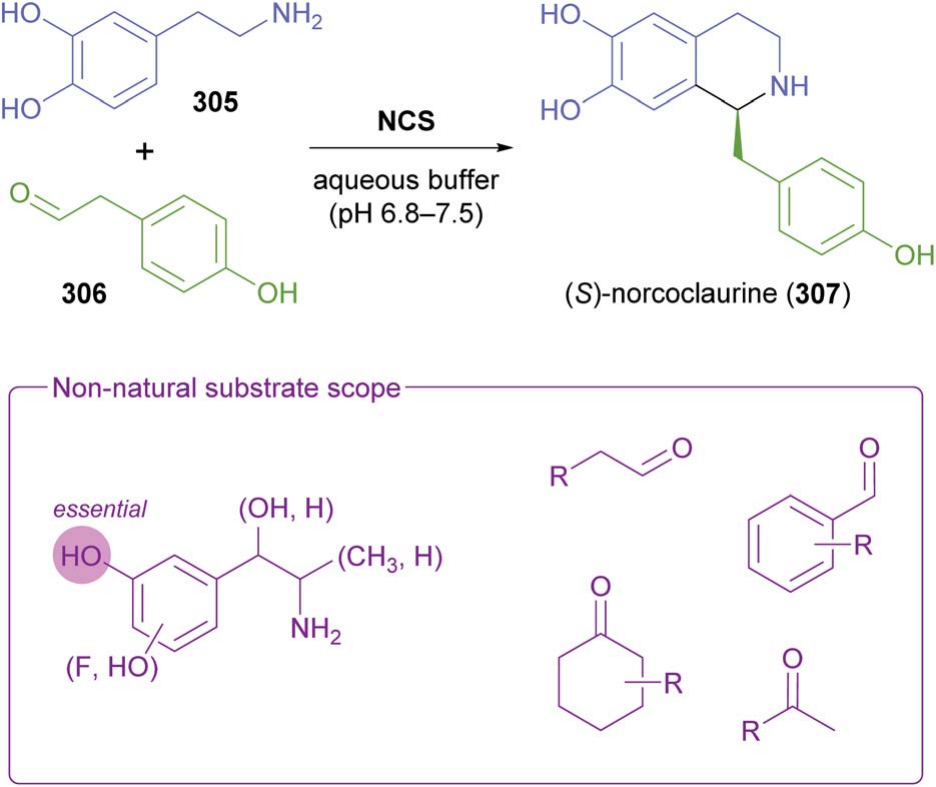
Natural reaction and non-natural substrate scope of norcoclaurine synthase (NCS).

Norcoclaurine synthase is responsible for the first committed step in benzylisoquinoline alkaloid biosynthesis in plants, the condensation of dopamine (305) and 4-hydroxyphenylacetaldehyde (306) yielding (*S*)-norcoclaurine (307, Scheme 39), which is the central biosynthetic precursor for benzylisoquinolines, protoberberines, morphinans, and the like.^157*b*,158^

**Scheme 40:**
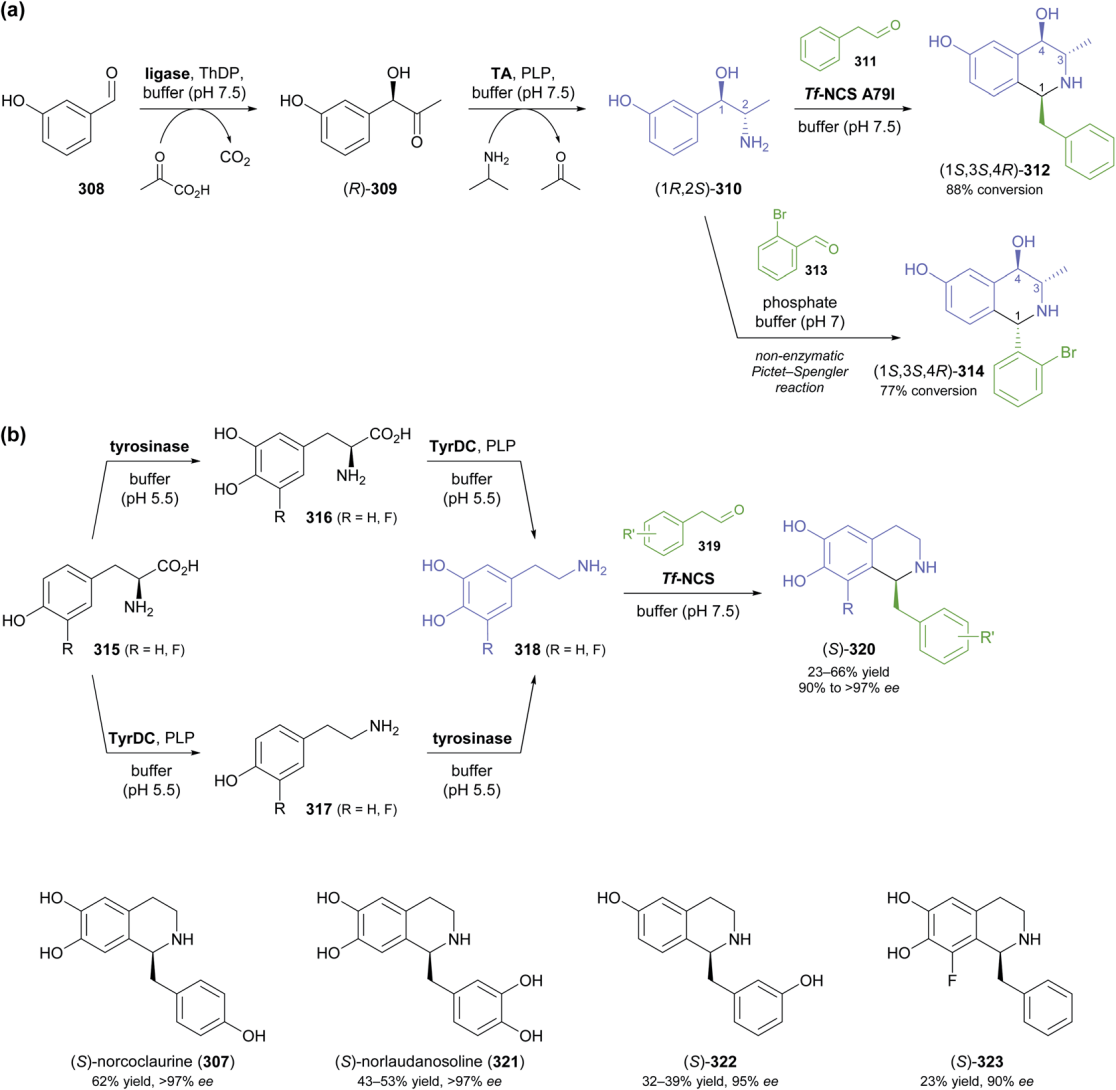
*In situ* formation of amine substrates for norcoclaurine synthase: (a) combination of biocatalytic carboligation and transamination leads to metaraminol (310), which can undergo enzymatic or phosphate-catalysed Pictet–Spengler reactions. (b) Tyrosinase and tyrosine decarboxylase (TyrDC) can be used to prepare dopamine and its derivatives from the corresponding amino acids.

Early investigations into the substrate scope of NCS, carried out using the enzymes from *Thalictrum flavum* (*Tf*-NCS) and *Coptis japonica* (*Cj*-NCS), have found the *meta*-hydroxyphenethylamine core of dopamine to be a strict structural requirement, in line with the currently accepted mechanism of NCS catalysis.^159^ This leaves opportunities for varied substitution in other positions of the aromatic ring and in the aliphatic side chain, some of which were explored in recent studies. Dörte Rother and co-workers used the carboligation–transamination sequence developed in earlier work by their group (*cf*. Scheme 36c, Section 5.2) to generate the amino alcohol metaraminol (310) with excellent stereocontrol (>99% *ee*, 97% *de*; Scheme 40a).^160^ This intermediate was then subjected to Pictet–Spengler reactions catalysed either by the A79I variant of NCS from *Thalictrum flavum* or by phosphate buffer. In the first case, using phenylacetaldehyde (311) as carbonyl substrate, the reaction delivered the benzylisoquinoline (1*S*,3*S*,4*R*)-312 with 88% overall conversion and >97% stereoisomeric purity thanks to the reliable (1*S*)-selectivity of norcoclaurine synthase. The phosphate-catalysed reaction, on the other hand, requires substrate-based stereocontrol, which was found to be limited (30–89% *de*) with 311 and several aromatic aldehydes. 2-Bromobenzaldehyde (313), however, underwent the Pictet–Spengler cyclisation to (1*S*,3*S*,4*R*)-314 with 77% overall conversion and complete diastereoselectivity. Notably, the (1*S*)-configuration of this compound reflects a switch in Cahn–Ingold–Prelog priorities, while the stereoselectivity of the reaction is actually opposite to that of the NCS biotransformation.

**Scheme 41:**
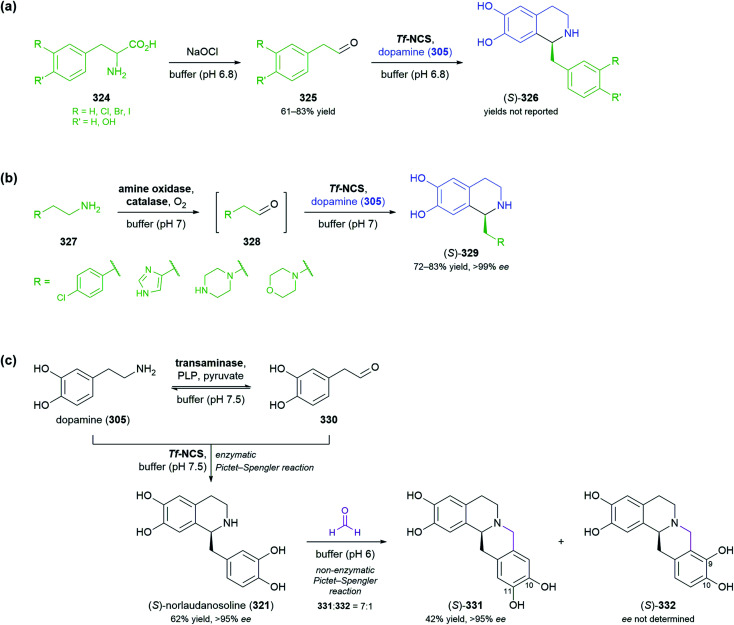
*In situ* formation of aldehyde substrates for norcoclaurine synthase: (a) oxidative decarboxylation of amino acids 324 with sodium hypochlorite, (b) enzymatic oxidation of primary amines 327 by an amine oxidase, and (c) enzymatic transamination of dopamine (305) in a “triangular cascade” leading to (*S*)-norlaudanosoline (321) and tetrahydroprotoberberines (*S*)-331 and (*S*)-332.

Ward, Hailes and co-workers investigated the formation of dopamine and close structural analogues 318 from the corresponding tyrosine derivatives 315 by the combined action of tyrosinases and tyrosine decarboxylases (TyrDCs) from various microbial sources (Scheme 40b).^161^ The best-performing enzymes showed a relatively broad substrate tolerance, and biotransformations that use both enzymes concurrently are therefore likely to convert 315 into 318*via* two competing pathways with 316 and 317 as the respective intermediates. Coupling of these hydroxylation and decarboxylation reactions with *Tf*-NCS-catalysed Pictet–Spengler cyclisation established multi-enzyme cascades, whereby the authors followed what they call a “mix and match” strategy: The carbonyl substrates for NCS were either added to the biotransformation or were generated *in situ* from amines 318 by a transaminase (*cf*. Scheme 41c below). Moreover, tyrosinase and TyrDC were used either in combination or individually, hence giving rise to a variety of (*S*)-benzylisoquinoline products. These include the natural alkaloids (*S*)-norcoclaurine (307) and (*S*)-norlaudanosoline (321), obtained as single enantiomers (>97% *ee*) in isolated yields of 62% (307) and 43–53% (321, depending on the batch size). Several non-natural derivatives (*e.g.*, 322 and 323) were also prepared, with yields ranging from 23% to 66% and *ee* values from 75% to >97%.

Compared to the amine substrate scope, the carbonyl scope of norcoclaurine synthase has been established as relatively broad already in early investigations, comprising a range of arylaliphatic and aliphatic aldehydes. Since many of these are unstable and difficult to synthesise, several studies have explored their *in situ* formation under the conditions of the NCS biotransformation. For instance, a previously reported method^162^ that generates arylacetaldehyde derivatives 325 from the corresponding amino acids 324 using sodium hypochlorite was extended to a broader range of substrates (Scheme 41a).^163^ The aldehydes 325 thus generated were either isolated in 61–83% yield or directly transformed further into (*S*)-benzylisoquinolines 326 using NCS from *Thalictrum flavum*. Unfortunately, only relative reaction rates but no conversion or stereoselectivity data were reported for the chemo-enzymatic sequence. Alternatively, primary amines can serve as precursors to aldehyde substrates of NCS. The quantitative conversion of four amines 327 into the corresponding aldehydes 328 using a diamine oxidase from *Lathyrus cicera* (red pea) was combined with a Pictet–Spengler reaction catalysed by *Tf*-NCS, affording non-natural (*S*)-benzylisoquinolines 329 in 72–83% yield and >99% *ee* (Scheme 41b).^164^ To prevent the undesired oxidation of dopamine, the second NCS substrate, this one-pot sequence was carried out in step-wise fashion and phenylhydrazine was used to inactivate the amine oxidase once its reaction was complete. In a related approach, a transaminase from *Chromobacterium violaceum* was used to deaminate dopamine (305) to aldehyde 330 (Scheme 41c).^165^ Only 0.5 equivalents of the amine acceptor, pyruvate, were employed so that the reaction remained incomplete, allowing the coupling of 330 and remaining 305 to (*S*)-norlaudanosoline (321) by *Tf*-NCS in what the authors have termed a ‘triangular cascade’. A preparative reaction on 0.5 mmol scale gave 321 in 62% isolated yield and excellent optical purity (>95% *ee*). The cascade was easily extended by adding formaldehyde after completion of the biotransformation, which triggered a non-enzymatic Pictet–Spengler reaction that led to the tetrahydroprotoberberines 331 and 332 as final products. Notably, this cyclisation proceeded with a 7 : 1 selectivity for the 10,11-disubstituted 331 over its 9,10-disubstituted regioisomer 332, making the method complementary to enzymatic cyclisation by berberine bridge enzyme (*cf*. Section 3.3). Implementing the three-step chemo-enzymatic process on 0.5 mmol scale resulted in the formation of 331 in 42% overall yield (from 47% conversion) and >95% *ee*.

**Scheme 42:**
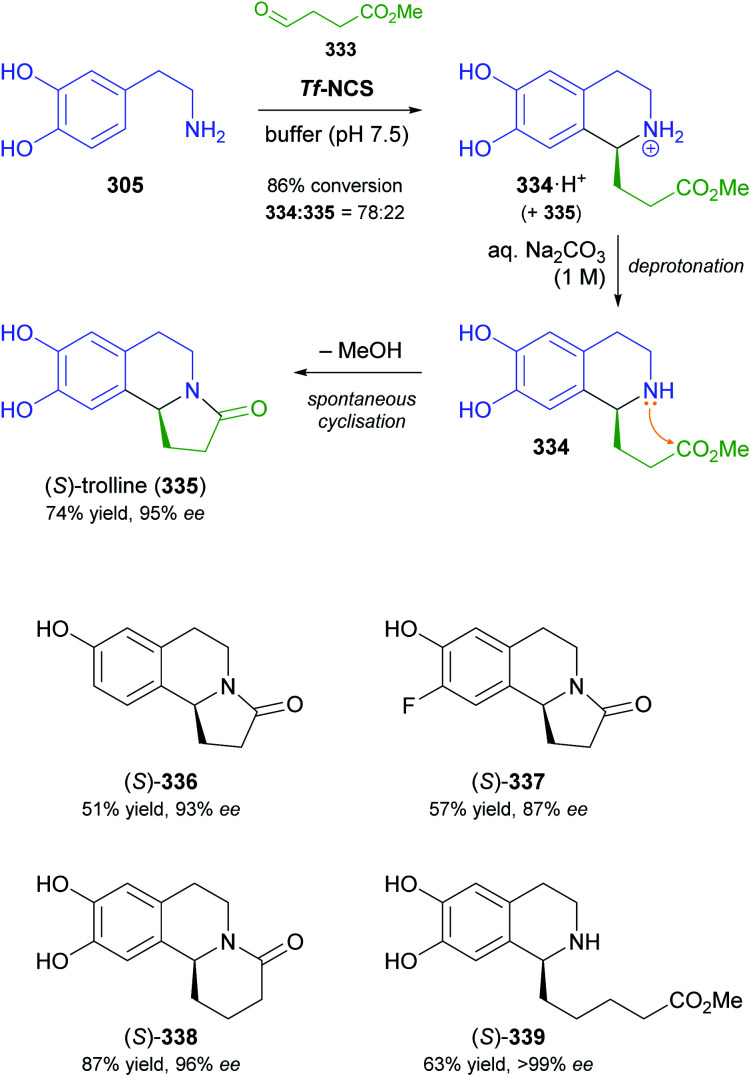
Synthesis of (*S*)-trolline (335) by enzymatic Pictet–Spengler reaction and base-promoted cyclisation, and examples of non-natural analogues prepared in the same way.

A non-enzymatic cyclisation was also exploited in the chemo-enzymatic synthesis of the globeflower alkaloid (*S*)-trolline (335) from dopamine (305) and aldehyde 333 (Scheme 42).^166^ The aliphatic aldehyde was readily accepted as substrate by *Tf*-NCS, which produced the expected tetrahydroisoquinoline 334 along with minor amounts of the target compound 335 in a combined 86% analytical yield. Subsequent basification of the reaction mixture by addition of aqueous Na_2_CO_3_ solution liberated the nucleophilic nitrogen atom in 334 by deprotonation and thus promoted its quantitative cyclisation to 335. (*S*)-Trolline was obtained by this simple one-pot procedure in 74% isolated yield and 95% *ee*. Through variation of the amine and carbonyl substrates, several non-natural derivatives of 335, including compounds 336–339, were prepared in analogous fashion. Notably, tetrahydroisoquinoline 339 could not be cyclised, reflecting the general difficulties associated with the formation of 7-membered rings.

**Fig. 4:**
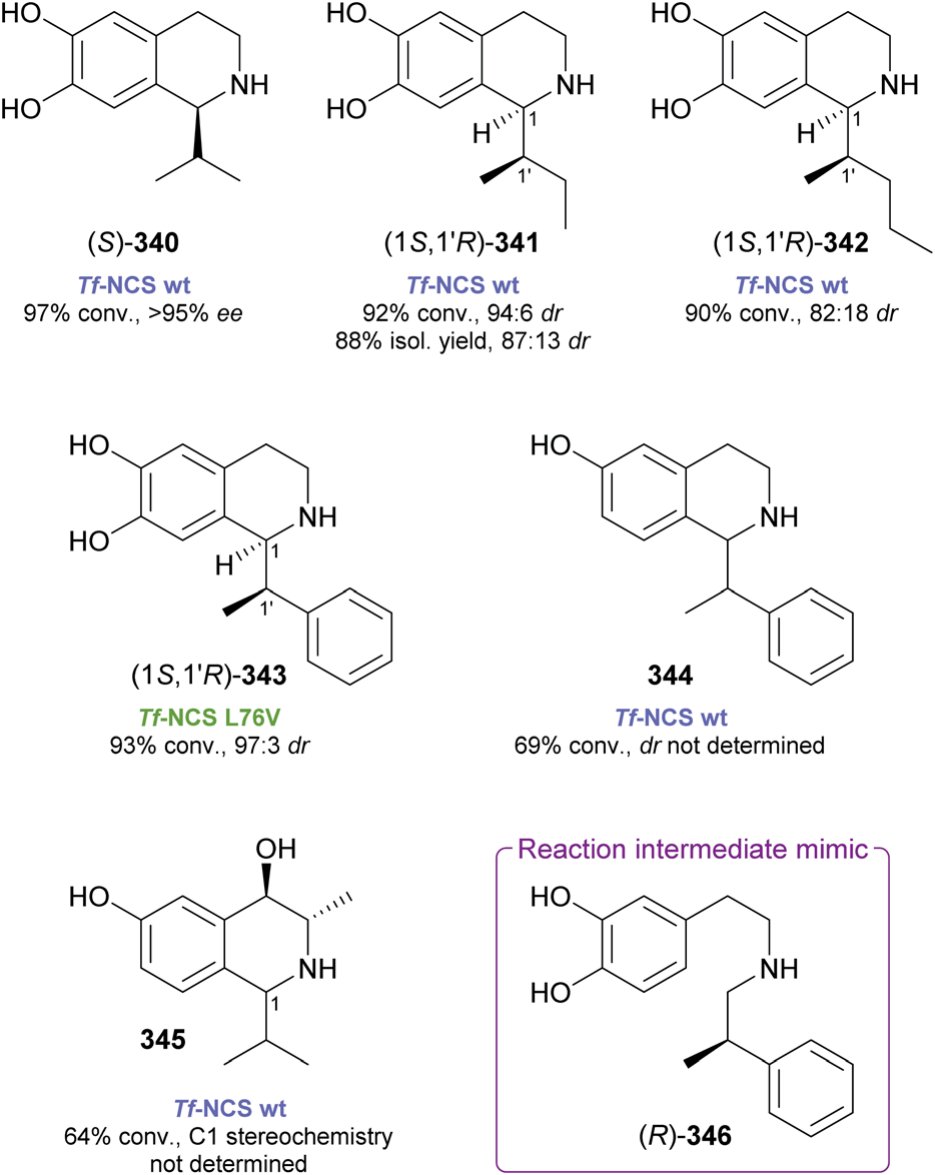
Examples of tetrahydroisoquinolines obtained by NCS reactions of α-branched aldehydes, and structure of a reaction intermediate mimic used for mechanistic studies.

A significant recent development regarding the substrate scope of norcoclaurine synthases is the finding that α-branched and α-cyclic aldehydes are accepted by wild-type NCSs and that this activity can be readily enhanced by protein engineering. First hints towards a potential acceptance of α-branched aldehydes were provided by studies using NCS from *Coptis japonica*, where small activities with aldehydes such as 2-methylpropanal (isobutanal) and 2-phenylpropanal were detected, but no reaction products were isolated.^167^ Much more detailed investigations were recently performed by Helen Hailes and co-workers, who found that NCS from *Thalictrum flavum* converts various α-methyl-substituted aliphatic and arylaliphatic aldehydes with good efficiency and stereoselectivity.^168^ For instance, 2-methylpropanal and dopamine were converted into the tetrahydroisoquinoline (*S*)-340 with near-complete conversion (97%) and >95% *ee* (Fig. 4). When chiral, racemic aldehydes were employed, the enzyme controlled not only the C1-configuration of the tetrahydroisoquinoline, but also the neighbouring side-chain stereocentre, typically affording (1*S*,1′*R*)-products in diastereomeric ratios of 82 : 18 and higher. Experiments performed with the individual enantiomers of 2-methylbutanal confirmed that this diastereoselectivity results from kinetic resolution of the racemates. Notably, the acceptance of α-branched aldehydes was not limited to reactions with the natural amine substrate, dopamine, as demonstrated by the formation of products such as 344 and 345. For some of the investigated reactions, the conversions and diastereoselectivities observed with wild-type *Tf*-NCS were even surpassed by previously reported enzyme variants, and the co-crystallisation of *Tf*-NCS with a reaction intermediate mimic, (*R*)-346, provided insights into the structural basis of these experimental findings. Moreover, a preparative biotransformation towards product (*S*)-341 showed good scalability (88% isolated yield on 0.5 g scale) and only a slight erosion of diastereoselectivity (87 : 13 *dr*) compared to the analytical-scale reaction (94 : 6 *dr*).

**Scheme 43:**
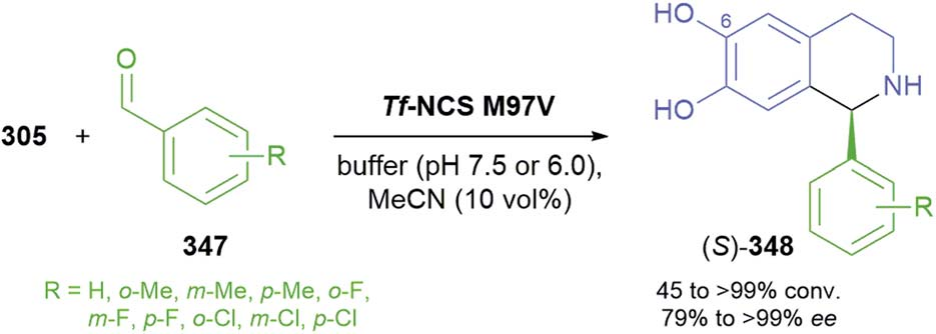
Synthesis of 1-aryl-tetrahydroisoquinolines 348 using variant M97V of NCS from *Thalictrum flavum*.

In a follow-up study published one year later, the authors could show that wild-type *Tf*-NCS and its variants also catalyse Pictet–Spengler reactions of cyclohexanecarbaldehyde, benzaldehyde, and many monosubstituted benzaldehyde derivatives (Scheme 43).^169^ In particular, the variant M97V proved to be very suitable for this class of substrates, generating a panel of (*S*)-1-aryl-tetrahydroisoquinolines 348 with moderate to high conversions (45% to >99%) and high to excellent stereoselectivities (79% to >97% *ee*). For the halogenated benzaldehydes, carrying out the biotransformation at pH 6 instead of the usual pH 7.5 led to improved *ee* values, which is partly due to a lower non-enzymatic background reactivity under the slightly acidic conditions. However, the change to a lower pH also improved analytical yields, indicating an increase in enzyme activity. Unsurprisingly, the substrates that led to the highest non-enzymatic background and consequently to the lowest product *ee* were the *o*-F and *o*-Cl derivative, in which the carbonyl group experiences significant activation by the electron-withdrawing effect of the halogen atom. To further increase the range of accessible products, the authors also investigated the methylation of two tetrahydroisoquinolines 348 by SAM-dependent *O*-methyltransferases from *Rattus norvegicus* and *Myxococcus xanthus*. Both enzymes were found to be active in these reactions, and the latter also proved completely regioselective for the C6 hydroxyl group.

**Fig. 5:**
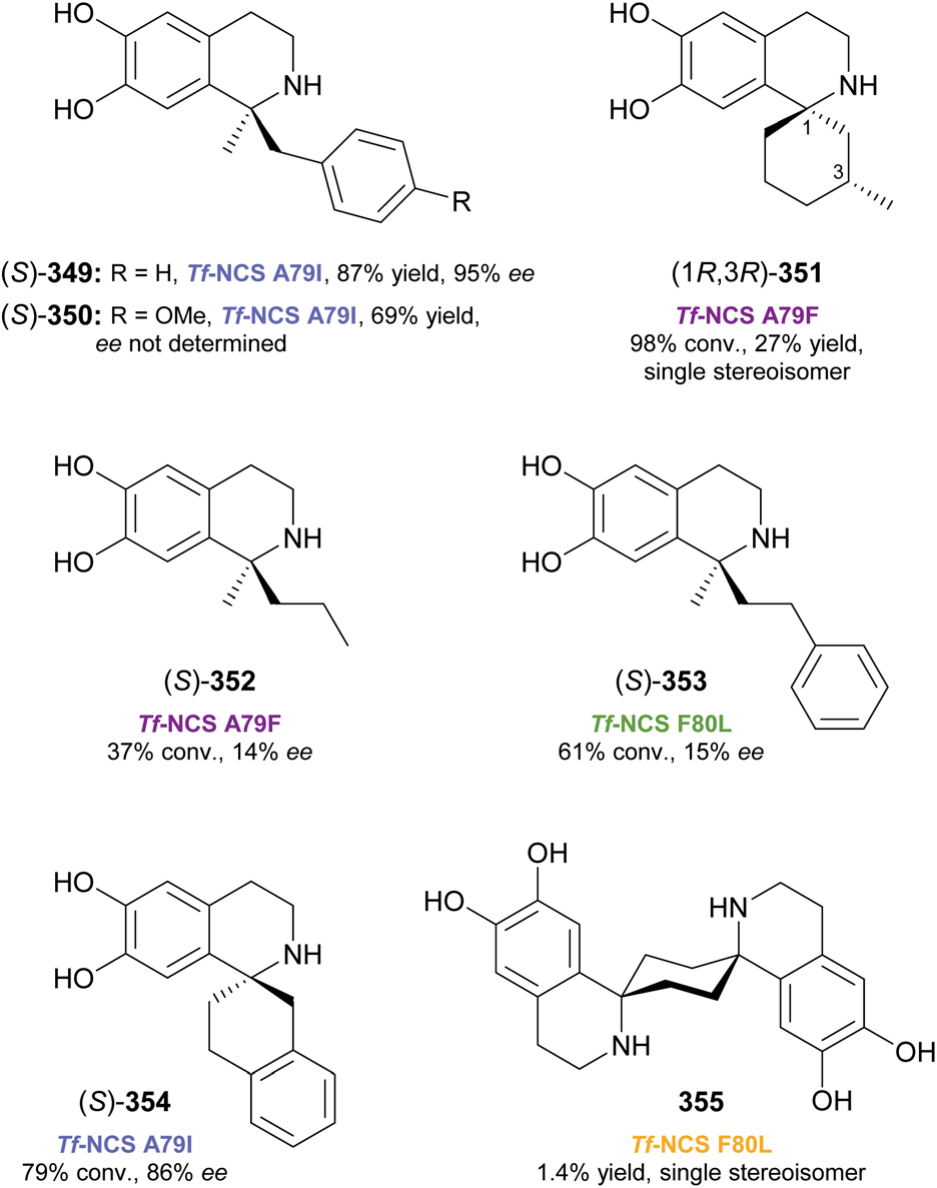
Examples of 1,1-disubstituted and spirocyclic tetrahydroisoquinolines obtained by NCS reactions of open-chain or cyclic ketones.

Perhaps even more surprising than the acceptance of α-branched aldehydes by norcoclaurine synthase is the finding that ketones are viable substrates for this enzyme class. This discovery was first made in 2017, when Ward, Hailes, and co-workers investigated the Pictet–Spengler reaction between dopamine and 18 structurally diverse ketones, using *Tf*-NCS and some of its variants as biocatalysts.^170^ Out of the diverse substrate panel, phenylacetone and its *para*-hydroxy and *para*-methoxy derivatives, cyclohexanone, and four monosubstituted cyclohexanone congeners were transformed by the enzymes, leading to 1,1-disubstituted and spirocyclic tetrahydroisoquinolines (Fig. 5). The products were formed with up to 99% conversion and could be isolated from preparative biotransformations (50 μmol scale) as their hydrochloride salts in fair to excellent yields. For instance, (*S*)-349 was obtained in 87% yield and 95% *ee*, and a reaction between dopamine and (*R*)-3-methylcyclohexanone gave (1*R*,3*R*)-351 in 27% yield and stereoisomerically pure form. Very recently, a broad and systematic investigation of the ketone substrate scope of *Tf*-NCS revealed many more interesting products to be accessible.^171^ Aliphatic ketones and 4-phenylbutan-2-one were found to be accepted, although the conversions to tetrahydroisoquinolines such as 352 and 353 were moderate (37%, 61%) and the stereoselectivities were poor (14%, 15%). Reactions with cyclic ketones gave generally good conversions (52–73%) and were also successful with non-natural amine substrates. The bicyclic 2-tetralone and its derivatives were transformed with surprising efficiency, as exemplified by the formation of (*S*)-354 with 79% conversion and 86% *ee*. The most impressive example, however, is probably the conversion of dopamine and cyclohexane-1,4-dione into the unusual dimeric spiro-tetrahydroisoquinoline 355, which was isolated in 1.4% yield and fully characterised by NMR, proving it to be stereoisomerically pure.

#### 5.3.2 Strictosidine synthase

**Scheme 44:**
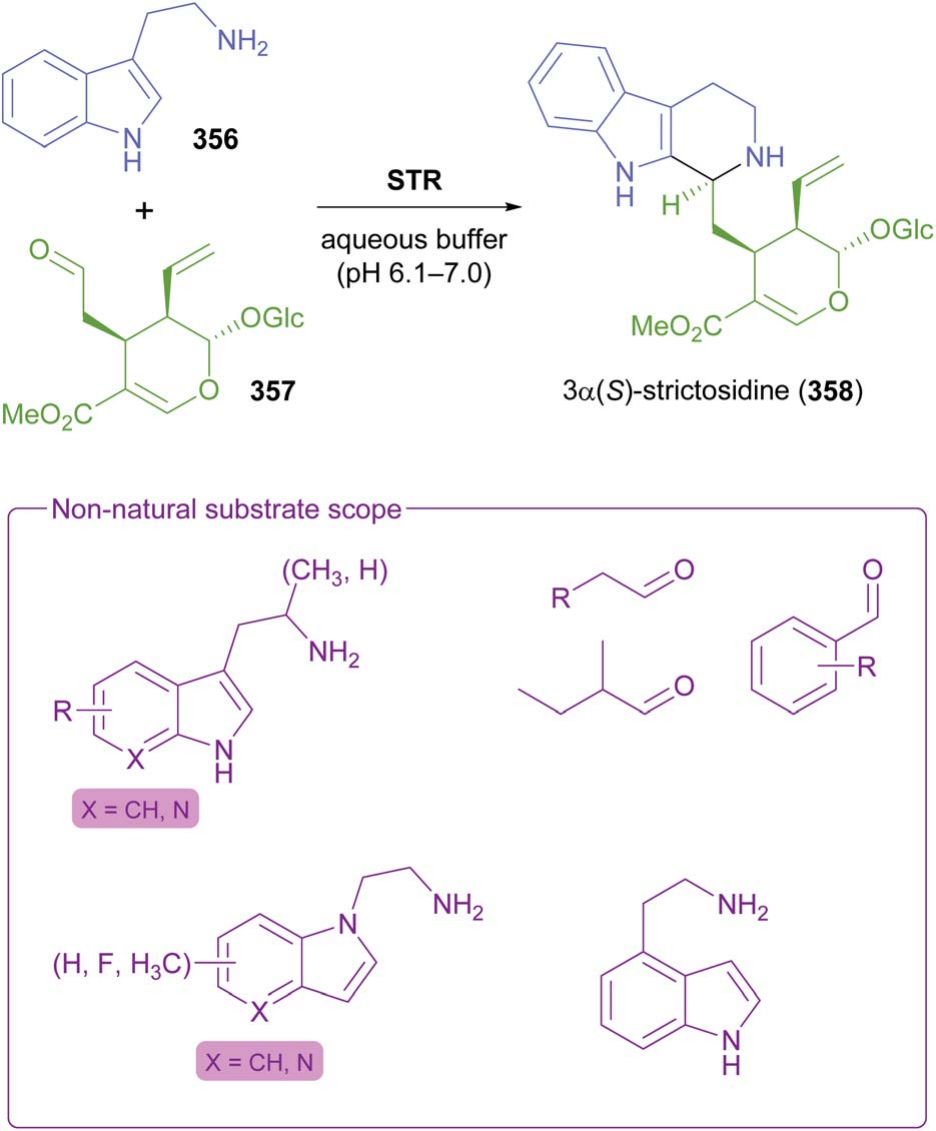
Natural reaction and non-natural substrate scope of strictosidine synthase (STR).

The second Pictet–Spenglerase that is of significance in biocatalysis is strictosidine synthase (STR). In nature, it catalyses the formation of 3α(*S*)-strictosidine (358) from tryptamine (356) and secologanin (357), giving access to a wide spectrum of monoterpenoid indole alkaloids (Scheme 44).^157*b*,158^

**Scheme 45:**
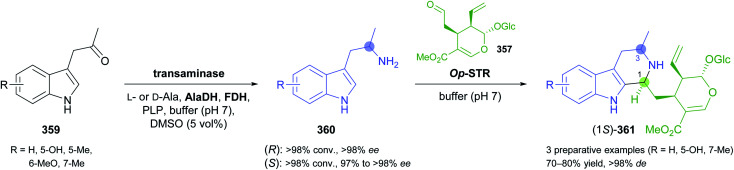
Biocatalytic one-pot synthesis of C3-methylated strictosidine derivatives 361 by the combination of transaminases and STR from *Ophiorrhiza pumila*. The stereogenic centres controlled by the transaminase and by STR are highlighted with blue and green circles, respectively.

In contrast to norcoclaurine synthase, early investigations into the substrate scope of STRs focused on variations of the amine partner, while largely relying on secologanin (357) and some of its close structural analogues as the aldehyde substrate. The same is true for a more recent study, in which (*R*)- or (*S*)-configured α-methyltryptamines 360, obtained from ketones 359 by transaminase reactions, were coupled with 357 by STR from *Ophiorrhiza pumila* (*Op*-STR) to give C3-methylated strictosidine derivatives 361 (Scheme 45).^172^ The amines 360 were prepared in 20–57% isolated yield and high optical purity (97% to >98% *ee*) for an initial activity screening with five STRs and detailed investigations of diastereoselectivity with *Op*-STR, the best-performing enzyme. The latter experiments revealed that *Op*-STR converts both amine enantiomers at comparable rates (*E* = 5–7 for 360, R = H) and that it controls the configuration at C1 of the tetrahydro-β-carboline product 361 to give the (1*S*)-diastereomer (>98% *de*) irrespective of the amine's absolute configuration. For preparative biotransformations, the transaminase and STR reactions were combined in a one-pot process, whereby a step-wise approach (addition of STR and 357 after completion of the transamination step) was slightly superior in terms of overall conversion compared to the simultaneous addition of all required enzymes and reagents. Three of the investigated indolylacetones (359, R = H, 5-OH, 7-Me) could thus be transformed into either diastereomer of (1*S*)-361 with high efficiency (56% to >99% overall conversion), and multi-milligram amounts of three products were isolated from pooled small-scale reactions in 70–80% yield and >98% *de*.

**Scheme 46:**
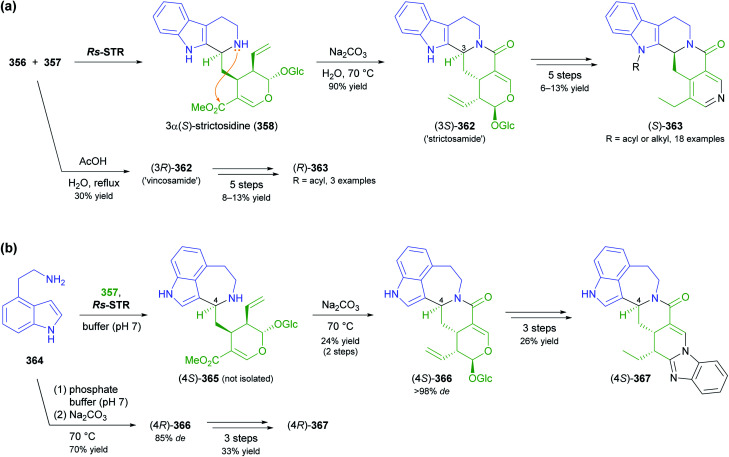
Applications of strictosidine synthase in medicinal chemistry: (a) chemo-enzymatic synthesis of *N*-substituted 3,14,18,19-tetrahydroangustines (363) as topoisomerase 1 inhibitors, (b) condensation of secologanin (357) with non-natural amine substrate 364 to give azepinoindole derivative (4*S*)-366, and its conversion into the antimalarial 367.

Two recent studies by Hongbin Zou and co-workers have focussed on the transformation of STR products into derivatives with potential medicinal application, while in one case also expanding the amine substrate scope of strictosidine synthase to a previously not investigated scaffold. In the first of these reports, the authors used the physiological reaction of STR from *Rauvolfia serpentina* to generate 3α(*S*)-strictosidine (358), which upon heating in aqueous Na_2_CO_3_ solution underwent cyclisation to (3*S*)-362, a known compound also referred to as ‘strictosamide’ (Scheme 46a).^173^ Its (3*R*)-epimer (‘vincosamide’), on the other hand, is directly accessible from tryptamine (356) and secologanin (357) through a non-enzymatic, acid-promoted Pictet–Spengler reaction. The lactams (3*S*)- and (3*R*)-362 were elaborated into a panel of 21 *N*-substituted tetrahydroangustines 363*via* a five-step sequence that involved an enzymatic deglycosylation, and the products were investigated as topoisomerase I inhibitors. In related work, the authors discovered that *Rs*-STR catalyses the coupling of secologanin (357) and 1*H*-indole-4-ethanamine (364) to azepinoindole (4*S*)-365 (Scheme 46b).^174^ Next to the natural tetrahydro-β-carbolines and piperazinoindoles generated in earlier studies,^175^ this represents the third distinct scaffold accessible through STR catalysis. The enzyme product (4*S*)-365 was not isolated but directly transformed into the more stable lactam (4*S*)-366, which was obtained in 24% isolated yield over the two steps and in >98% *de*. Catalytic hydrogenation, enzymatic deglycosylation, and condensation with benzene-1,2-diamine then furnished compound (4*S*)-367, which was shown to exhibit antimalarial activity. Similar to the case of ‘vincosamide’ discussed above, the (4*R*)-epimers (85% *de*) were accessible through a non-enzymatic Pictet–Spengler reaction, which in this work was performed using phosphate catalysis.

**Scheme 47:**
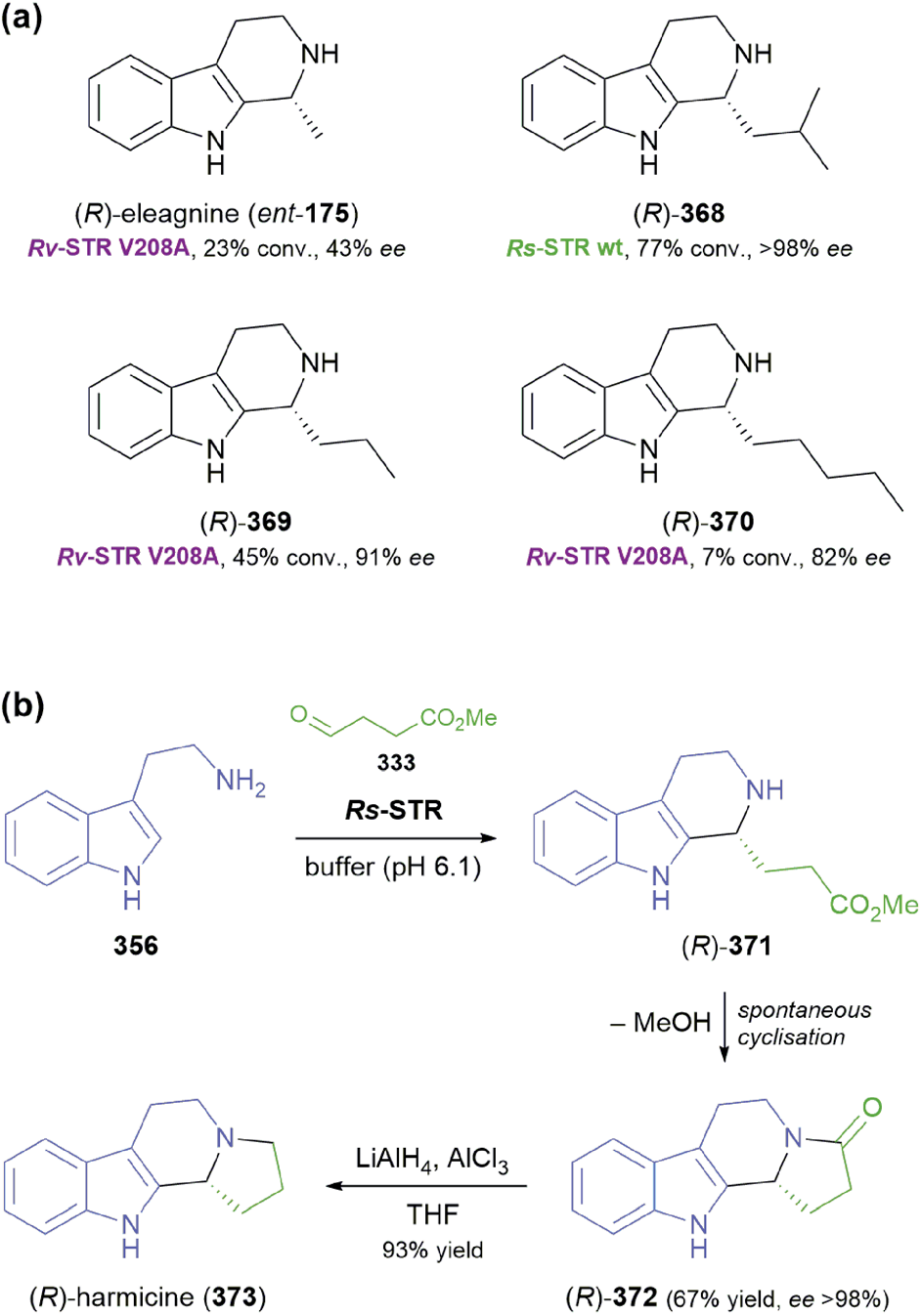
(*R*)-Selective Pictet–Spengler reactions of strictosidine synthases (STRs): (a) 1-alkyl-tetrahydro-β-carbolines obtained from tryptamine (356) and small, aliphatic aldehydes, (b) two-step chemo-enzymatic synthesis of (*R*)-harmicine (373).

Substantial variations of the aldehyde substrate of strictosidine synthase were first reported in 2018, when it was shown that the STRs from *Catharanthus roseus*, *Ophiorrhiza pumila*, and *Rauvolfia serpentina* (as well as the V208A variant of the latter) readily accept small aliphatic aldehydes.^176^ Surprisingly, the resulting tetrahydro-β-carbolines 175 and 368–370 (Scheme 47a) were formed with (*R*)-stereoconfiguration, in several cases even with >98% *ee*.^177^*Rs*-STR and its V208A variant gave the best conversions and stereoselectivities across the tested range of substrates, and the wild type was selected for a preparative application: the condensation of tryptamine (356) and aldehyde 333 catalysed by *Rs*-STR afforded – after spontaneous cyclisation of the immediate reaction product 371 – the lactam (*R*)-372 in 67% isolated yield and >98% *ee* (Scheme 47b; *cf*. Scheme 42, Section 5.3.1). Subsequent reduction of 372 by lithium aluminium hydride provided the alkaloid (*R*)-harmicine (373) and hence completed an asymmetric synthesis of this compound in only two steps (62% yield overall) from commercially available starting materials.

**Scheme 48:**
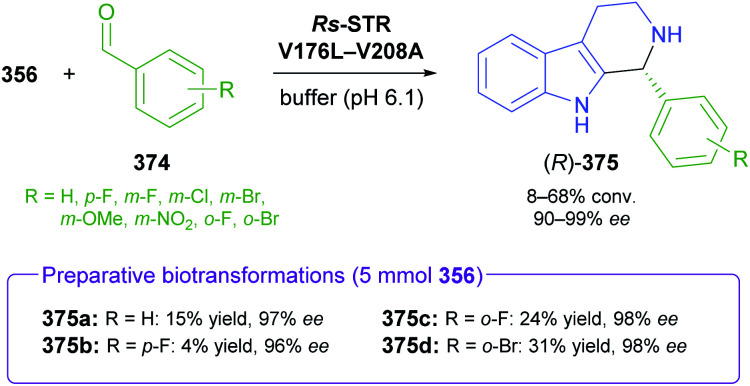
Synthesis of 1-aryl-tetrahydro-β-carbolines 375 using variant V176L–V208A of STR from *Rauvolfia serpentina*.

A further expansion of the carbonyl substrate scope of strictosidine synthase to benzaldehyde derivatives was recently achieved through protein engineering.^178^ After an initial screening of three wild-type STRs with benzaldehyde found no activity, and 2-methylbutanal gave barely detectable conversions (0.2–1.0%), the authors followed a ‘substrate walking’ approach:^179,180^ First, variants with increased activity towards 2-methylbutanal were identified and only the best of these were then tested with benzaldehyde as substrate. Moreover, the protein engineering was initially performed on STR from *Ophiorrhiza pumila* and beneficial mutations were later transferred to the *Rauvolfia serpentina* enzyme. The best variant, *Rs*-STR V176L–V208A, was active on nine benzaldehyde derivatives 374 and furnished the corresponding 1-aryltetrahydro-β-carbolines (*R*)-375 with 8–68% conversion and 90–99% *ee* (Scheme 48). In particular, *ortho*- and *meta*-substitution was well tolerated, while aldehydes with a *para*-substituent larger than fluorine were not converted. Four biotransformations were carried out on preparative scale (5 mmol of tryptamine) and afforded products (*R*)-375 in moderate yields (4–31%) but high optical purities (96–98%).

### 5.4 Other enzymes

**Scheme 49:**
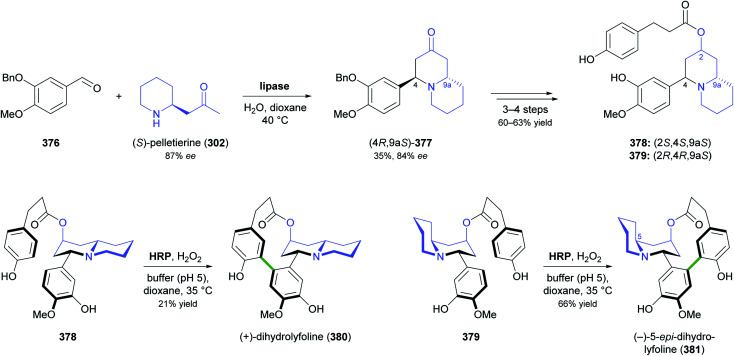
Synthesis of (+)-dihydrolyfoline (380) and (−)-5-*epi*-dihydrolyfoline (381) *via* lipase-catalysed Mannich reaction and horseradish peroxidase (HRP)-catalysed oxidative phenol coupling.

The biocatalysts discussed in the previous subsections – aldolases, transaminases, and Pictet–Spenglerases – have enabled the preparation of a wide range of alkaloids. More specialised enzymatic key bond formations can also be used in alkaloid synthesis with good success, as demonstrated by three recent studies. Interestingly, all three fall in the broad category of C–H functionalisation. In the first of these reports, the crape myrtle constituents (+)-dihydrolyfoline (380; non-natural enantiomer) and (−)-5-*epi*-dihydrolyfoline (381) were obtained *via* horseradish peroxidase-catalysed oxidative cyclisation of the bis-phenols 378 and 379, respectively (Scheme 49).^181^ Although a single coupling product was formed in both cases, indicating high regio- and diastereoselectivity, the reaction towards 381 proceeded with significantly higher yield (50–66%) than that leading to 380 (15–21%). The coupling precursors 378 and 379 were prepared in a 4–5 step sequence that involved a lipase-catalysed Mannich reaction between *O*-benzylated isovanillin (376) and (*S*)-pelletierine (302, itself an alkaloid) as another enzymatic transformation.

**Scheme 50:**
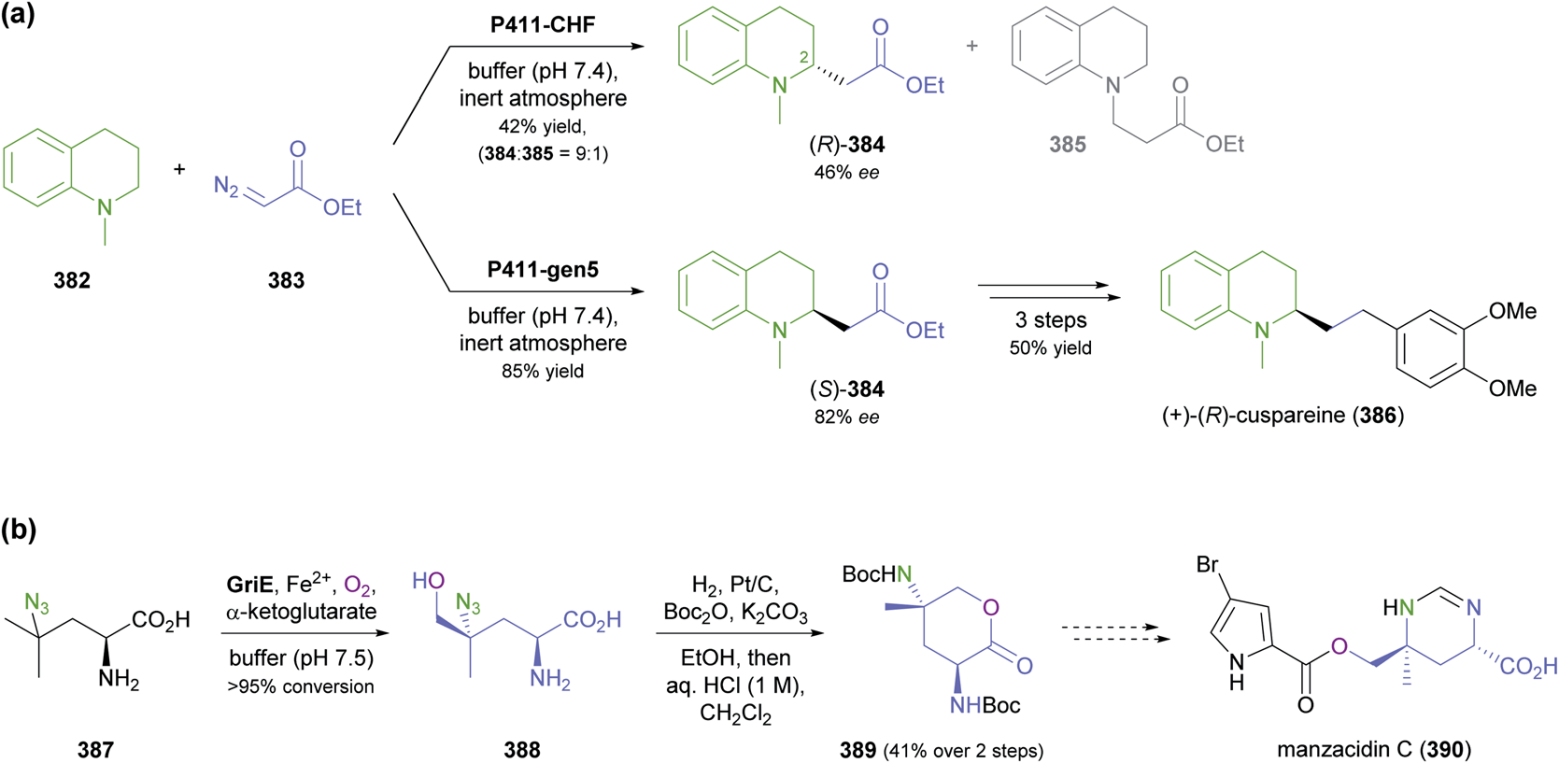
Recent applications of biocatalytic sp^3^ C–H functionalisation in alkaloid synthesis: (a) α-alkylation of *N*-methyl-1,2,3,4-tetrahydroquinoline (382) using cytochrome P411 variants, (b) terminal hydroxylation of amino acid 387 catalysed by an iron(ii)/α-ketoglutarate-dependent oxidase, GriE.

In recent years, the scope of biocatalytic carbon–carbon and carbon–heteroatom bond formation has been expanded impressively through non-natural reactions of engineered heme proteins that exploit the diverse reactivity of iron(ii) carbene or nitrene intermediates.^182^ In particular, by replacing the conserved axial cysteine ligand in cytochromes P450 with serine, Frances Arnold's research group has created a novel class of heme-dependent enzymes, termed cytochromes P411, which are capable of catalysing cyclopropanation, aziridination, and C–H amination reactions.^183^ In a recent study, Arnold and co-workers have demonstrated that cytochromes P411 also catalyse C–C bond formation *via* sp^3^ C–H activation.^184^ While this work mainly focused on benzylic and allylic substrates, alkylation of amines in α-position of the nitrogen atom was also achieved, and the preparative value of this transformation was demonstrated by using it as the key step in the asymmetric synthesis of the alkaloid (+)-(*R*)-cuspareine (386; non-natural enantiomer; Scheme 50a). The main P411 variant investigated in this study, P411-CHF, catalysed the coupling of *N*-methyl-1,2,3,4-tetrahydroquinoline (382) and ethyl diazoacetate (383) with good regioselectivity (9 : 1 for C2 over the *N*-methyl group) and moderate preference for the (*R*)-enantiomer (46% *ee*). An intermediate variant from the directed evolution campaign (P411-gen5) displayed better regio- (>50 : 1) and stereoselectivity (82% *ee*) with inverted stereopreference, affording (*S*)-384 in 85% yield on half-gram scale. Reduction of the ester to a primary alcohol, conversion of the latter into a bromide, and Suzuki coupling completed the synthesis of (+)-cuspareine (386).

Biocatalytic C–H oxidation has occasionally been used in alkaloid synthesis, usually for late-stage functionalisation.^185,186^ Zwick and Renata, however, have recently used C–H hydroxylation catalysed by an iron(ii)/α-ketoglutarate-dependent dioxygenase, GriE, as the asymmetric key step in the formal synthesis of manzacidin C (390), a sea sponge alkaloid (Scheme 50b).^187^ Compound 387, obtained by a photocatalytic C–H azidation of l-leucine, was locally desymmetrised by the biocatalytic terminal hydroxylation, giving alcohol 388 along with minor amounts of the bis-hydroxylation product. Directly subjecting this crude mixture to hydrogenation and Boc protection led to lactone 389 in 41% yield, a compound that can be converted into manzadicin C (390) *via* two literature-known^188^ steps. Hence, a remarkably concise formal synthesis of 390 was enabled by two consecutive C–H functionalisations of an unprotected amino acid.

## 6 Emerging biocatalysts from alkaloid biosynthetic pathways

A quick survey of the reactions discussed in the previous sections shows that the majority of chemo-enzymatic alkaloid syntheses (approx. 60%) make use of generalist enzymes with broad availability, such as lipases, transaminases, amine oxidases, or imine reductases. However, the secondary metabolic pathways through which alkaloids are assembled in nature hold in store a dazzling array of more specialised enzymes that could be applied in synthesis.^189^ Indeed, the successful examples of berberine bridge enzyme, norcoclaurine synthase, and strictosidine synthase demonstrate that once the protein expression issues often associated with plant enzymes are solved, these can become valuable biocatalysts with broad application potential.

Recent advances in genomics, proteomics, and metabolomics have accelerated the elucidation of biosynthetic pathways and the identification of enzymes on a sequence level.^190^ This development, combined with the ever-decreasing costs of DNA synthesis,^191^ makes a wealth of prospective biocatalysts accessible for experimental characterisation and synthetic application, which we believe will further expand the scope of biocatalysis in alkaloid synthesis.

One area with considerable potential for diversification are enzymatic Pictet–Spengler reactions: As discussed in Section 5.3.1, norcoclaurine synthase (NCS) from *Thalictrum flavum* has proven outstandingly flexible in terms of its carbonyl substrate acceptance. NCS sequences have been identified in many other organisms, and some have recently been heterologously expressed and studied in their natural reaction,^192^ but their non-natural substrate scope remains to be explored. Moreover, Pictet–Spenglerases other than NCS and STR are known, but most of them have not yet been evaluated in a biocatalytic context.^157*a*,193^

**Scheme 51:**
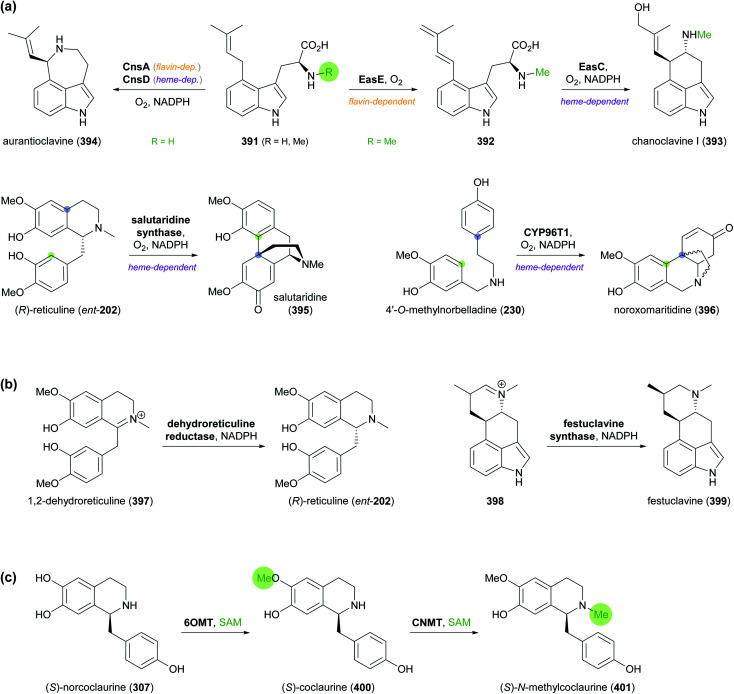
Examples of enzymatic transformations from alkaloid biosynthetic pathways that have potential for biocatalytic application: (a) oxidative cyclisations, (b) reduction of iminium ions, and (c) regioselective methylations.

Oxidative cyclisations are common in alkaloid biosynthetic pathways, where they usually proceed with levels of regio- and stereoselectivity that are unmatched by chemical catalysts. Some of the responsible enzymes are close structural homologues of BBE,^194^ a flavin-dependent oxidase that has proven its preparative applicability (*cf*. Section 3.3), while another large group are heme-dependent oxidoreductases. The recently elucidated transformations of *N*-methyl-4-(dimethylallyl)tryptophan (391, R = Me) to chanoclavine I (393) and of 4-(dimethylallyl)tryptophan (391, R = H) to aurantioclavine (394) both require one enzyme from each of these groups and involve not only a cyclisation but also a decarboxylation (Scheme 51a).^195^ Oxidative phenol coupling reactions catalysed by cytochromes P450 constitute key steps in the biosynthesis of morphinans (formation of salutaridine, 395)^196^ and *Amaryllidaceae* alkaloids (*e.g.*, noroxomaritidine, 396),^197^ among others. The enzymes responsible for these transformations are membrane-bound and their natural redox partners are not yet known, which complicates their functional heterologous expression and their use *in vitro*. However, if these hurdles could be overcome and these enzymatic cyclisations could be translated to preparatively useful conditions, they would unlock new synthetic approaches to the respective families of natural products.

Imine reduction has become an established part of the biocatalytic toolbox of reactions over the last ten years, but virtually all work in this area relies on enzymes from a single protein fold class. Alkaloid biosynthesis in plants and fungi involves C=N reduction by enzymes that are structurally unrelated to the common IREDs.^198^ Since these reductases have evolved to transform relatively large and complex substrates (*e.g.*, 397, 398, Scheme 51b), their use in preparative biotransformations could expand the scope of biocatalytic imine reduction.

Finally, regioselective methylations catalysed by *S*-adenosyl methionine (SAM)-dependent methyltransferases are commonplace in secondary metabolism (for two examples from benzylisoquinoline alkaloid biosynthesis, see Scheme 51c),^199^ and some of the involved enzymes have already found first applications in biocatalysis (*cf*. Scheme 28).^126,200^ Since recent studies have introduced practical options for an *in situ* regeneration of the SAM cofactor and some analogues with modified alkyl groups,^201^ the importance of biocatalytic alkylations can be expected to grow,^202^ and methyltransferases from alkaloid biosynthetic pathways might play a significant role in this context.

## 7 Conclusions and outlook

**Fig. 6:**
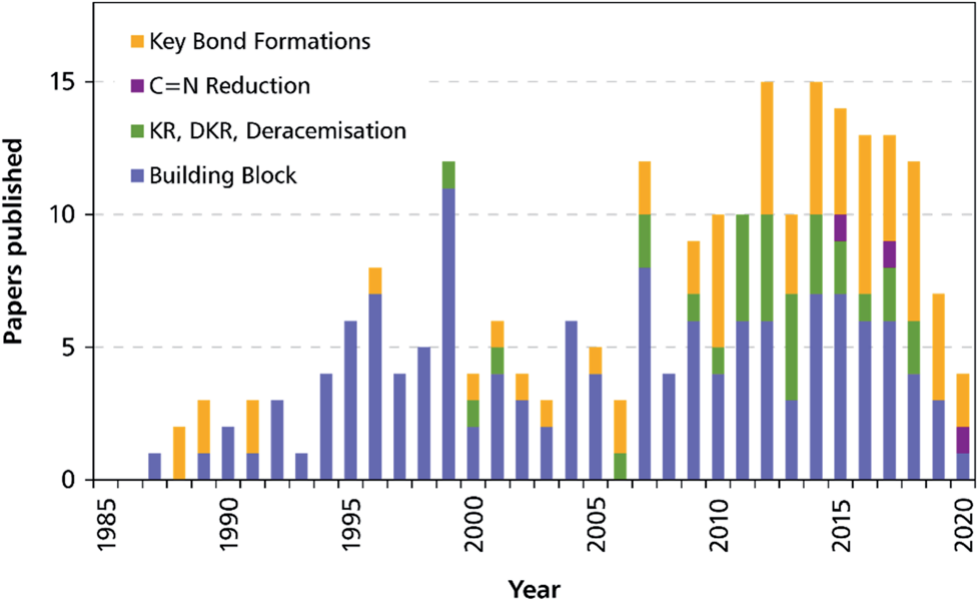
Chemo-enzymatic total syntheses of alkaloids published per year. The colours indicate the different chemo-enzymatic approaches as discussed in this review: (a) biocatalytic preparation of chiral building blocks (blue), (b) biocatalytic kinetic resolution, dynamic kinetic resolution, and deracemisation of alkaloids (green), (c) biocatalytic reduction of imines or iminium ions (purple), and (d) chemo-enzymatic syntheses that use enzymes for key asymmetric C–C and/or C–N bond formation (orange).

Over the last eight years, the role of biocatalysis in the asymmetric synthesis of alkaloids has continued to evolve. Chiral building block approaches relying on lipases, esterases, and toluene dioxygenase still account for the largest number and the greatest structural diversity of alkaloids prepared through chemo-enzymatic routes, but biocatalytic key C–C and C–N bond formations have steadily grown in importance and are now almost equally common (Fig. 6). Kinetic resolution, dynamic kinetic resolution, and deracemisation of alkaloids continue to be explored, but are largely restricted to target molecules with only a single centre of chirality, which severely limits their scope. The same is true for biocatalytic imine reduction, which has recently emerged as a novel option for alkaloid synthesis, but has not yet had a significant impact in a preparative context.

**Fig. 7:**
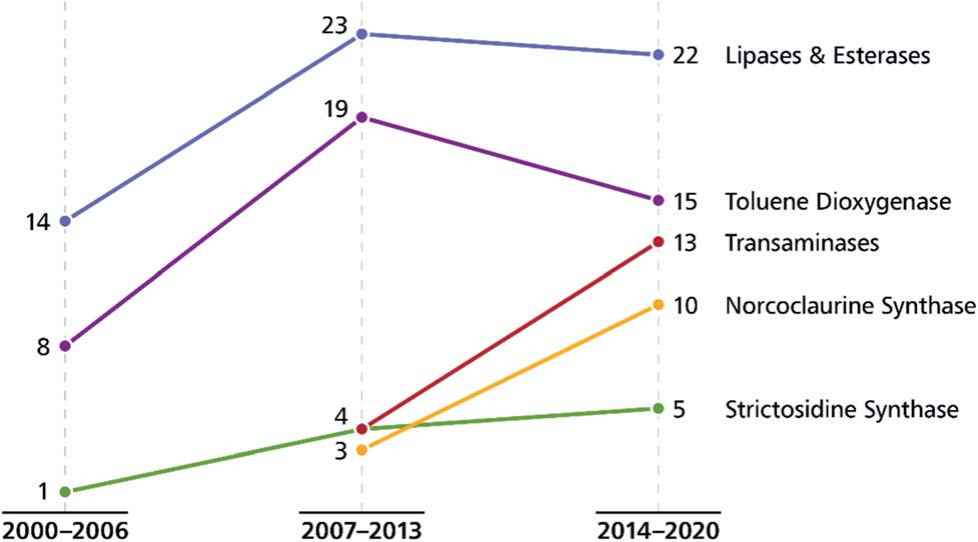
Use of selected enzyme classes in chemo-enzymatic alkaloid synthesis over the last 21 years, expressed as the number of published reports grouped into 7 year periods.

The observed shift towards key bond formations can mainly be attributed to the vastly increased use of two enzyme classes in alkaloid synthesis: transaminases and norcoclaurine synthases (Fig. 7). Neither of the two groups has found application in the chemo-enzymatic synthesis of alkaloids before 2010, but both have quickly risen in importance after that point. The broad substrate scope of both enzyme families is a significant factor in this development, which becomes particularly evident when comparing norcoclaurine synthase with strictosidine synthase: Both enzymes catalyse Pictet–Spengler reactions that directly lead to alkaloid scaffolds, but the former accepts a much more diverse range of carbonyl substrates and has hence found more widespread use than the latter. Irrespective of this difference, both Pictet–Spenglerases can be considered prime examples of plant enzymes that have become successful biocatalysts, and we anticipate that many more enzymes of alkaloid biosynthesis – in particular those forming carbon–carbon bonds – will find their way into preparative applications in the future.

The increasingly ‘central’ use of biocatalytic transformations in chemo-enzymatic routes towards alkaloids has given rise to many syntheses that are considerably shorter than purely chemical alternatives and that require little or no use of protective groups. These characteristics bode well for applications on larger scale, and the example of (−)-ephedrine, produced industrially *via* biocatalytic carboligation and transition metal-catalysed reductive amination since almost a century,^203^ shows that chemo-enzymatic alkaloid syntheses can indeed be economically viable. Compared to the traditional isolation of natural products, a significant benefit of synthetic methods is their independence of plant material, while their main advantages over fermentation are higher space-time yields and a broader product portfolio.

Perhaps the last step required for an increased transfer of the chemo-enzymatic approaches discussed herein to production scale is a more intense communication and collaboration between synthetic chemists and researchers in biocatalysis, biotechnology and enzymology. Recognising the often complementary strengths of chemo- and biocatalytic methods and strategically exploiting them in synthetic planning should enable concise and efficient routes towards many target compounds, including alkaloids of practical relevance.^204^ We hope that this review, by showcasing a broad spectrum of chemo-enzymatic strategies for alkaloid synthesis ranging from chemistry-dominated to primarily biocatalytic, makes a small contribution to achieving this goal.

## Abbreviations

9-BBN: 9-Borabicyclo[3.3.1]nonane
Ac: Acetyl
AIBN: Azobisisobutyronitrile
ADH: Alcohol dehydrogenase
Ala: Alanine
AlaDH: l-Alanine dehydrogenase
BBE: Berberine bridge enzyme
Bn: Benzyl
Boc: *tert*-Butoxycarbonyl
Bz: Benzoyl
CAL-B: Lipase B from *Candida antarctica*
Cbz: Carboxybenzyl (benzyloxycarbonyl)
CNMT: Coclaurine *N*-methyltransferase
CSA: Camphorsulfonic acid
DAIB: (Diacetoxyiodo)benzene
DBAD: Di-*tert*-butyl azodicarboxylate
DBU: 1,8-Diazabicyclo[5.4.0]undec-7-ene
DCE: 1,2-Dichloroethane
DEAD: Diethyl azodicarboxylate
DHAP: Dihydroxyacetone phosphate
DIAD: Diisopropyl azodicarboxylate
DIBAL: Diisobutylaluminium hydride
DKR: Dynamic kinetic resolution
DMAP: 4-Dimethylaminopyridine
DME: 1,2-Dimethoxyethane
DMF: *N*,*N*-Dimethylformamide
DMSO: Dimethyl sulfoxide
dba: Dibenzylideneacetone
dppe: 1,2-Bis(diphenylphosphino)ethane
dppf: 1,1′-Bis(diphenylphosphino)ferrocene
*de*
: Diastereomeric excess
*dr*
: Diastereomeric ratio
*E*
: Enantioselectivity
*E. coli*
: *Escherichia coli*
*ee*
: Enantiomeric excess
FDH: Formate dehydrogenase
FSA: d-Fructose-6-phosphate aldolase
FucA: l-Fuculose-1-phosphate aldolase
GDH: d-Glucose dehydrogenase
Glc: d-Glucopyranosyl
HBTU: 2-(1*H*-Benzotriazol-1-yl)-1,1,3,3-tetramethyluronium hexafluorophosphate
HLE: Horse liver esterase
HRP: Horseradish peroxidase
IP-NBSH: *N*′-Isopropylidene-2-nitrobenzenesulfonohydrazide
IRED: Imine reductase
*k*_cat_
: Turnover number (Michaelis–Menten kinetics)
LDA: Lithium diisopropylamide
LDH: Lactate dehydrogenase
Moc: Methoxycarbonyl
MOM: Methoxymethyl
MS: Molecular sieves
MTBE: Methyl *tert*-butyl ether
NADH: β-Nicotinamide adenine dinucleotide
NADPH: β-Nicotinamide adenine dinucleotide phosphate
NBS: *N*-Bromosuccinimide
NCS: Norcoclaurine synthase
NMR: Nuclear magnetic resonance
PAD: Potassium azodicarboxylate
PCC: Pyridinium chlorochromate
PDC: Pyruvate decarboxylase
PLE: Pig liver esterase
PLP: Pyridoxal 5′-phosphate
PMB: *para*-Methoxybenzyl
RhuA: l-Rhamnulose-1-phosphate aldolase
rt: Room temperature
SAM: *S*-Adenosyl methionine
STR: Strictosidine synthase
STY: Space-time yield
TA: Transaminase
TBD: 1,5,7-Triazabicyclo[4.4.0]dec-5-ene
TBDMS: *tert*-Butyldimethylsilyl
TBDPS: *tert*-Butyldiphenylsilyl
TDO: Toluene dioxygenase
TFA: Trifluoroacetic acid
TFAA: Trifluoroacetic anhydride
ThDP: Thiamine diphosphate
THF: Tetrahydrofuran
TIPS: Triisopropylsilyl
TMAD: Tetramethylazodicarboxamide
TMS: Trimethylsilyl
Ts: Tosyl (*p*-toluenesulfonyl)
TyrDC: l-Tyrosine decarboxylase
wt: Wild type

## Conflicts of interest

There are no conflicts to declare.

## Supplementary Material

RA-011-D1RA04181A-s001
