# Supplementary material for: The role of biocatalysis in the asymmetric synthesis of alkaloids – an update
Source: RSC Adv. 2021 Aug 20;11(45):28223–70. doi: 10.1039/d1ra04181a (PMC9038100; doi:10.1039/d1ra04181a)
Supplement: RA-011-D1RA04181A-s001 [file RA-011-D1RA04181A-s001.pdf]

Electronic Supplementary Material (ESI) for RSC Advances.
This journal is © The Royal Society of Chemistry 2022

Developments in Chemo-Enzymatic Alkaloid Synthesis via Biocatalytic Transformations

RSC Advances

Biocatalysts are a valuable tool for alkaloid synthesis

Biocatalysis

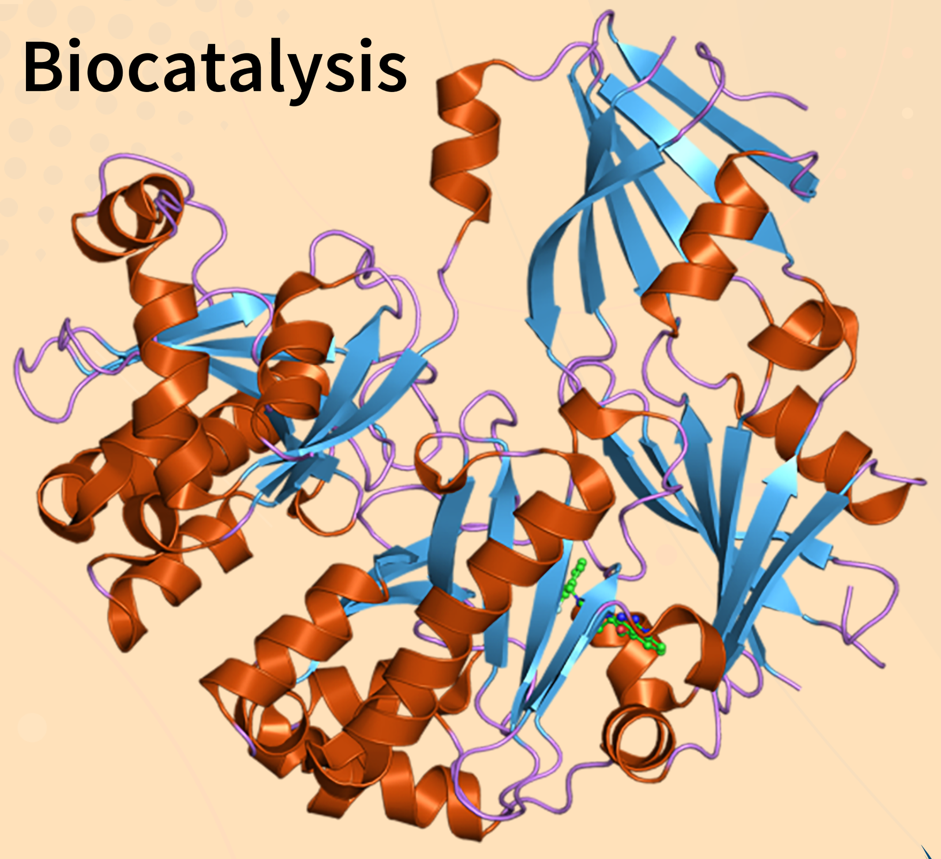

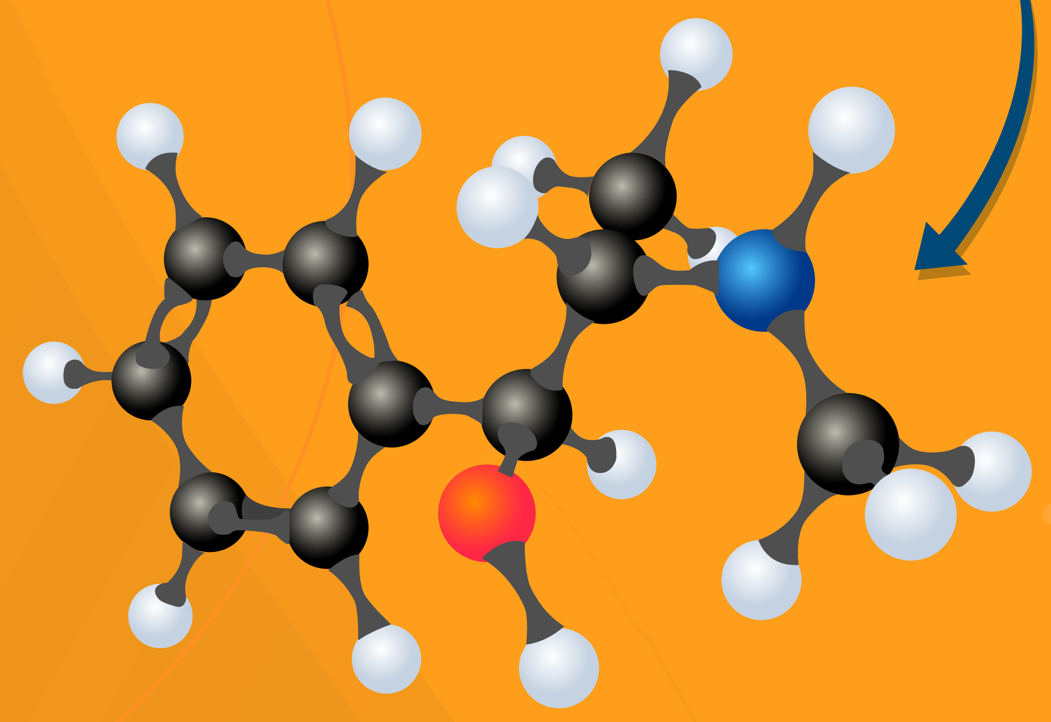

Alkaloid synthesis

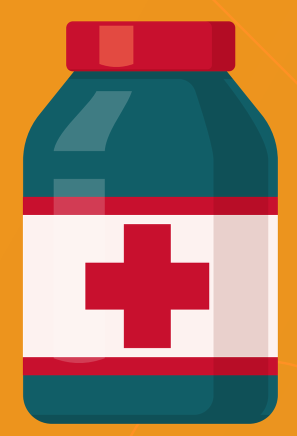

Medicinal use

Integration of biocatalysts for chemo-enzymatic alkaloid synthesis could solve large-scale production challenges

Modern developments in biocatalytic engineering and alkaloid synthesis

C-C
C-N

Biocatalytic key C-C and C-N bond formation

C=N

Biocatalytic imine reduction

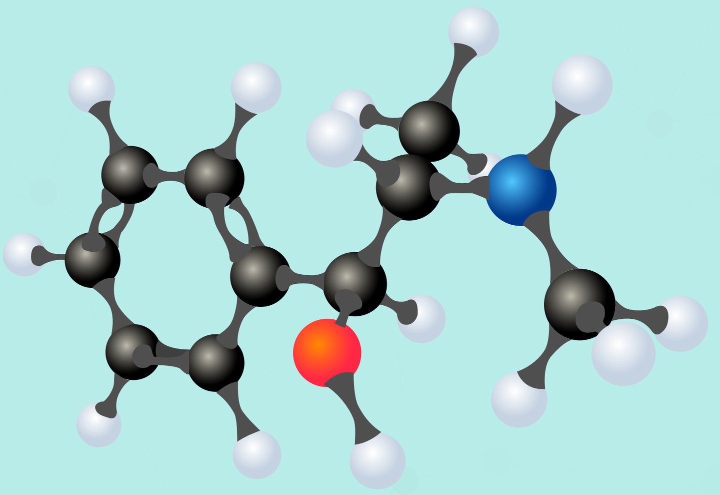

Alkaloid synthesis

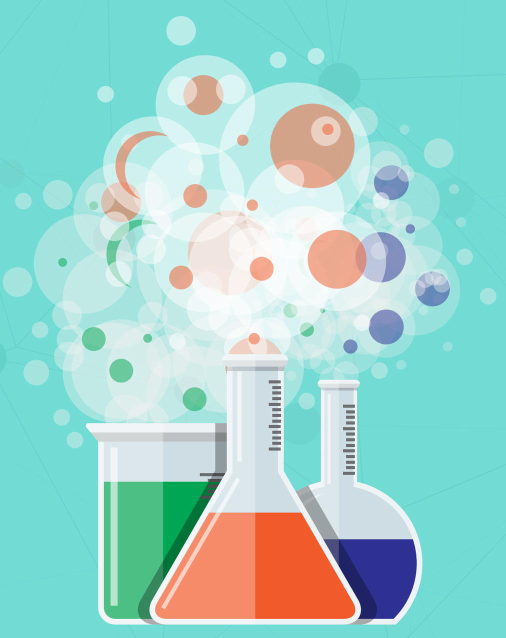

Chiral building block approach

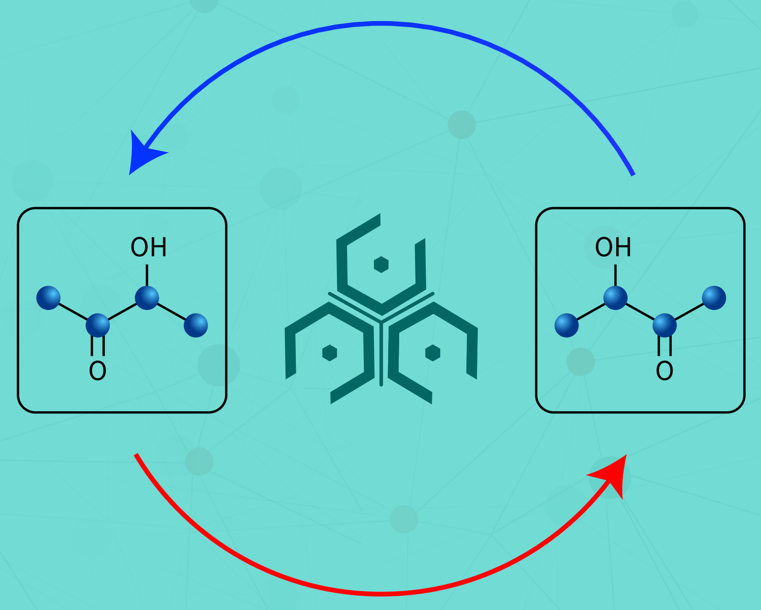

Kinetic resolution, dynamic kinetic resolution, and deracemisation

Shorter chemo-enzymatic syntheses are an economically viable option

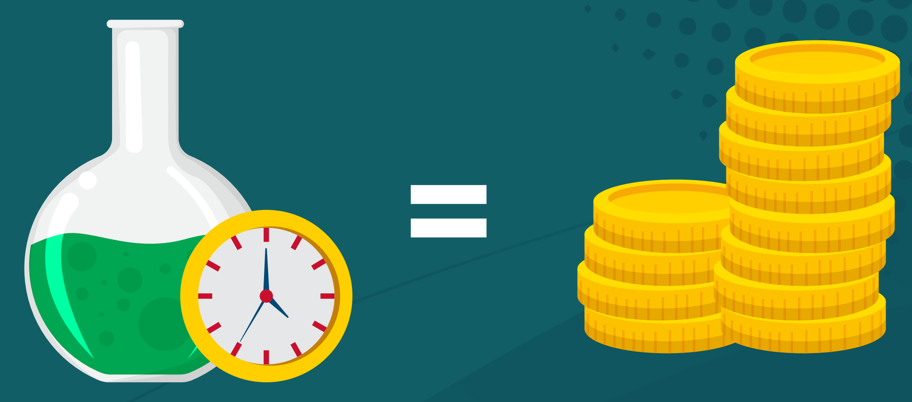

Collaborative efforts are essential to exploiting the new chemo-enzymatic approaches to alkaloid synthesis

The role of biocatalysis in the asymmetric synthesis of alkaloids – an update
Schrittwieser et al. (2021) | RSC Advances | DOI: 10.1039/D1RA04181A

ROYAL SOCIETY OF CHEMISTRY
